# Design of Nanomaterial-Based Sensors for Enhanced Halogen Bonding

**DOI:** 10.1021/acsomega.5c07542

**Published:** 2026-01-14

**Authors:** Ben H. Edelman, Charles W. Sheppard, Lucas A. Chuidian, Arielle Vinnikov, Felix Bevc, Lillian B. Hughes, Carol A. Parish, Kevin W. Kittredge, Michael C. Leopold

**Affiliations:** † Department of Chemistry and Biochemistry, Virginia Wesleyan University, Virginia Beach, Virginia 23455, United States; ‡ Department of Chemistry, Gottwald Center for the Sciences, 6888 University of Richmond, Richmond, Virginia 23173, United States

## Abstract

Halogen bonding is a highly directional, noncovalent, intermolecular interaction which has been harnessed for a variety of applications, including sensor design. A halogen bond (XB) is formed between a region of positive electrostatic potential on a halogen atom (X) and electron rich portions of target molecules. The strength of XB interactions relies on shorter XB bond distances and more linear R–X···B bond angles, which facilitate stronger, more negative binding energies. While prior studies have sought to maximize interactions, few have explored or experimentally demonstrated how geometries and bond angles can enhance XB interactions. Herein, fundamental studies are conducted at self-assembled monolayers (SAMs) and gold nanoparticle (Au-NP) interfaces that are functionalized to engage in XB interactions. Alkanethiolate-stabilized Au-NPs, known as monolayer-protected gold clusters (MPCs), were enhanced with XB-donor capability by incorporating specialized XB donor thiol ligands including halogen terminated perfluorinated straight chain and rigid perfluoro-aromatic amide ligands—both of which were used within nanomaterial composite films of single-walled carbon nanotubes (SWCNTs) as a sensing interface in both solution and the gas phase. DFT and vapor studies targeting cyclohexanone (CH), a known byproduct of hard-to-detect, nonvolatile explosives (e.g., RDX), produced a sensing interface that achieved detection limits of CH (<10 ppm) that markedly outperform similar systems. The materials and methods presented in this study further demonstrate the potential of XB systems as a rapid and sensitive step toward developing field sensors for explosives.

## Graphical abstract

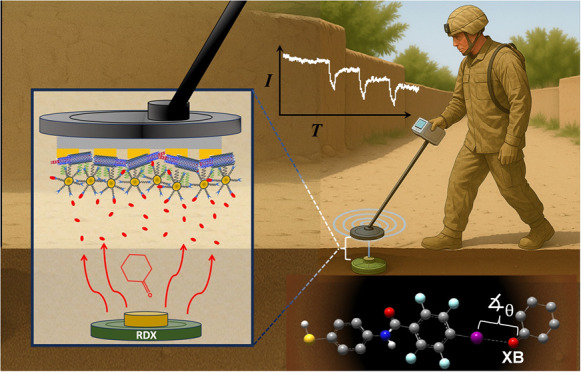

## 1 Introduction and Background

Halogen bonding (XB) is a highly directional, noncovalent interaction between an XB donor molecule containing a region of positive electrostatic potential on a halogen atom (X) and an electron-rich XB acceptor molecule or Lewis base.^1–3^ The angular dependence of XB is more restricted than hydrogen bonding (HB) because the halogen-based σ-hole must align optimally with the lone pairs on the XB acceptor.^3^ The size of the σ-hole is highly tunable and depends on the electronegativity and polarizability of the halogen atom as well as the strength and proximity of the e^–^ withdrawing groups (EWGs) that pull electron density away from the halogen atom to generate the σ-hole.^3,4^

XB has been explored with computation tools in small inorganic and organic molecular systems^5^ as well as within DNA base pairs.^6^ Experimental evidence and/or application of XB appear less frequently in the literature.^7^ We are particularly interested in probing the angular dependence of XB interactions. A survey of the literature specifically for experimental evidence for the directional nature of XB interactions shows that it has been used in crystal engineering^8^ and that XB can be preferred to hydrogen-bonding (HB) in various solutions and crystal structures.^9,10^ In addition to X-ray crystallography,^11,12^ X-ray assisted charge density techniques^11,12^ and microwave spectroscopy^13,14^ have been used to measure R–X···B bond lengths as evidence for XB interaction, though they were mostly focused on gas-phase model molecules of relatively simple structure. In 2022, Raman spectroscopy was used to demonstrate that XB can drive temporary conformational changes in polymer networks.^15^ The linearity of XB has been utilized in anion binding schemes utilizing titration calorimetry or spectroscopic measurements, though neither study exclusively measured the Θ in XB interactions.^16,17^

XB has been employed as a component of sensor design, and excellent reviews on the topic are available.^10,18,19^ One common XB sensor application is the detection of explosives such as trinitrotoluene (TNT) or 1,3,5-trinitro-1,3,5-triazinane (RDX).^20–19,20^

These materials contain e^–^ rich nitro groups as a major structural component (Scheme 1). The nonaromatic explosive compounds such as RDX are more challenging to detect due to their low vapor pressures. For example, the vapor pressure of RDX (∼8.3 × 10^–10^ Torr) is 4 orders of magnitude lower than that of TNT (∼4.8 × 10^–6^ Torr).^20^ While direct detection of these compounds would be ideal, there are other molecules that can be present with explosives that offer indirect detection. These molecules include byproducts from the explosive production process itself along with more volatile molecules, known as taggants, purposely added by manufacturers to enable detectability of the material. Headspace GC-MS analysis shows that volatile *byproducts*, such as cyclohexanone (CH) (∼1 × 10° Torr), are emitted during the synthesis/recrystallization of RDX.^23^ Similarly, production of explosives in the United States involves intentional doping of material with a semivolatile taggant additive, dimethyl-dinitrobutane or DMDNB (Scheme 1) (∼1 × 10^–3^ Torr).^23,24^

**Scheme 1:**
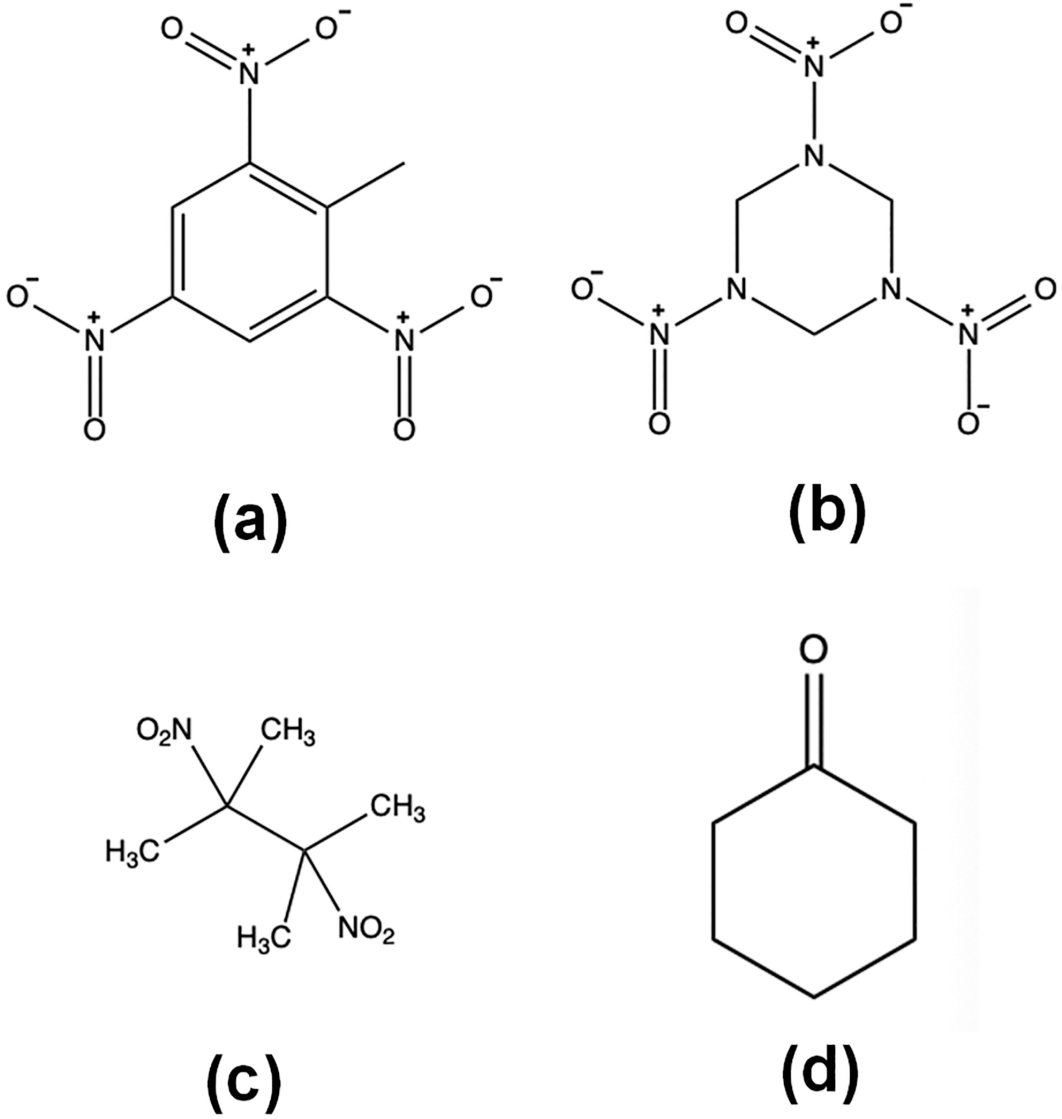
Examples of Explosive or Explosive Related Molecular Structures as Potential XB Acceptor Molecules Including (a) Aromatic TNT Explosive; (b) Non-Aromatic RDX Explosive; (c) 2,3-Dimethyl-2,3-dinitrobutane (DMDNB) Taggant and; (d) by-Product Cyclohexanone (CH)

The incorporation of nanomaterials (NMs) within sensing schemes improves sensitivity/performance and enables miniaturization of devices.^25–27^ NMs exhibit size-dependent electronic/spectroscopic properties that can be functionally harnessed in electrochemical/optical sensing schemes, in many cases enhancing signal-to-noise ratios. One of the most common NMs used in sensors is functionalized carbon nanotubes (CNTs)^28,29^ Swager and co-workers have employed functionalized single-walled CNTs (SWCNT), dispersed on interdigitated array (IDA) electrodes, as the basis for chemiresistive, gas-phase sensors that target cyclohexanone (CH) or taggants (e.g., nitromethane).^29–31^ That continuing body of work has utilized SWCNTs that have been covalently and noncovalently functionalized as well as polymer-wrapped SWCNTs that exploit HB intermolecular interactions in their sensor function.^32,33^ Swager’s lab has also designed SWCNTs modified with dihalogen XB donors that show detectable changes in conductance upon interaction with pyridine.^22^ Most recently, Beer and co-workers used XB interactions in cyclodextrin-based host–guest chemistry to optically detect chemical warfare agents in solution.^34–21^ Previously, we have shown that IDAs modified with SWCNTs and noncovalently attached dihalo-perfluorinated aromatics (e.g., di-iodo-tetrafluorobenzene), produced conductivity changes in the presence of CH (XB acceptor) that aligned with theoretical considerations of XB strength including halogen-electronegativity and polarizability along with the impact of EWGs on the size of the σ-hole.^21^

Computational methods can assist in the understanding of XB interaction by providing atomistic information. Three metrics are typically used to evaluate the strength of a XB interaction: binding energies (*E*_int_) between the XB donor and acceptor molecules, XB bond length (R), and R–X···B bond angle (Θ). Because the σ-hole is generated along the bonding axis, more linear Θ with shorter R values indicate stronger XB interaction and can be correlated with more negative *E*_int_ values.^4–6,37,38^ In general, most experimental studies tend to emphasize *E*_int_ and R and are less focused on the implications of R–X···B bond angles (Θ) or interaction geometries.

In the research presented here, we pair unique XB donor ligands with model XB acceptor molecules to assess how the chemical structure and sterics, likely altering XB bond angle may influence XB strength. Electrochemical solution experiments combined with computational modeling were used to develop a sensor that outperforms previous results.^21,31^ These results provide further understanding of XB behavior that will lead to more effective harnessing of this important interaction in future sensing designs.

## 2 Materials and Methods

### 2.1 General Chemicals and Instrumentation

Unless otherwise stated, all chemicals were purchased commercially and used without further purification while all aqueous solutions were prepared with 18.02 MΩ ultrapurified (UP) water. Bruker Avance NMR spectrometers (400 or 500 MHz) were used for chemical structure and nanoparticle (NP) characterizations with Mestrelab’s MestreNova (v15.0.1) used to analyze chemical shifts relative to tetramethylsilane (TMS). A photodiode array spectrophotometer (Agilent 8453) was used to collect UV–vis spectra of NP solutions. Transmission electron microscopy imaging was performed on a JEOL 1010 with Advanced Microscopy Techniques XR-100 CCD image collection (80–100 kV) after samples were dispersed on 400 mesh Formvar-coated copper grids (Electron Microscopy Sciences) with images analyzed as described previously^36^ to estimate average NP diameter (*n* ≥ 100/sample). Controlled chemical vapor concentrations delivered by permeation tube technology from Kin-tek Analytical Inc. (FlexStream Base Module) with heated-traced output lines.

### 2.2 Computational Methods

Density functional theory (DFT) was used to determine geometries and energies for individual molecules, as well as complexes formed between L2 and each of the Lewis base analytes: 1-benzlypiperidine (1-BP), cyclohexanone, 1–4-diazabicyclo [2,2,2] octane (DABCO), 2,3-dimethyl-2,3-dinitrobutane (DMDNB), 1,3,5-trinitroperhydro-1,3,5-triazine (RDX), and 2-methyl-1,3,5-trinitrobenzene (TNT). While molecular geometries of L1 and L2 were characterized, the majority of the computational efforts on XB complexes involved the L2 XB donor ligand because it showed the strongest experimental sensitivity to the various analytes (*vide infra*). The B3LYP,^39–41^ M06–2X^42^ and ωB97X-D^43^ functionals were utilized along with the def2-TZVDP,^44^ and correlation consistent double and triple-ζ basis sets cc-pVDZ and cc-pVTZ.^45^ To characterize iodine on L2, we used the ECP28MDF effective core pseudopotential^46^ along with the corresponding cc-pVDZ-PP and cc-pVTZ-PP basis sets,^47^ obtained from the Stuttgart/Cologne group library.^47,48^ To determine the methodological dependence of our results, we performed geometry optimizations using B3LYP/cc-pVDZ, M06–2X/cc-pVDZ, ωB97X-D/cc-pVDZ and ωB97X-D/def2-TZVDP, along with energy refinement using single point calculations on the M06–2X/cc-pVDZ and ωB97X-D/cc-pVDZ geometries with the cc-pVTZ basis set. We performed all calculations in the gas phase, and the M06–2X calculations in both the gas phase and using Truhlar’s SMD implicit solvent model for water.^49^ Harmonic vibrational frequency analysis was used to ensure all structures were local minima on their potential energy surfaces (PES). The energy of interaction between L2 and each of the five analytes was used to evaluate the strength of the halogen bonds. Those energies were calculated as

1 $$E_{\mathrm{interaction}}=E_{\mathrm{complex}}-\left(E_{\mathrm{analyte}}+E_{L2\mathrm{sensor}}\right)$$

Optimized X···B bond distances and R–X···B angles were analyzed to compare bonding strengths across analytes. Stronger halogen bonding complexes were generally characterized by shorter X···B distances and more linear R–X···B angles.^6,21,37,38,50,51^ Calculations utilized the Gaussian 16 ^52^ or Q-Chem V5.4.1 ^53^ suite of software. All Gaussian generated outputs were visualized using Gaussview6, while Q-Chem outputs were visualized with the open source interface IQMol. Electrostatic potential (ESP) maps were visualized on a 0.01 au electron density isosurface.

### 2.3 Cyclic Voltammetry Measurements

Cyclic voltammetry was performed by CH Instruments potentiostats (Models 650A, 630B, 610B and/or 420A) in glass electrochemical “sandwich” cells that feature an Ag/AgCl (saturated KCl) reference electrode (Microelectrodes, Inc.), a platinum wire (Sigma-Aldrich) counter electrode, and an evaporated gold substrate (EMF Corp., Ithaca, NY) as a working electrode defined by a Viton O-ring (0.32 cm^2^)—schematic representation provided in Supporting Information: Figure SI-1.^54^ Two types of cyclic voltammetry (CV) experiments, scanning from initial toward positive potentials in all cases, have been used in concert to measure XB interactions at film modified electrodes.^55^ Voltammetry was run for a minimum of three complete cycles to ensure reproducibility of behavior with last scans usually depicted in result comparisons. First, double-layer capacitance (*C*_dl_) measurements can be used to show the presence/absence of molecules interacting at an interface via electrostatic, covalent or other intermolecular interactions like XB. Experimentally, *C*_dl_ can be quantified by running CV in the absence of a redox species (i.e., only supporting electrolyte) to measure the non-Faradaic background or charging current at 0.250 V (vs Ag/AgCl, satr. KCl) reference and applying the following equation

2 $$C_{\mathrm{dl}}\left(\mu F/{cm}^{2}\right)=\frac{\left|i_{cathodic}+i_{anodic}\right|\left(amps\right)}{2\cdot \nu_{\left(V/\sec\right)}\cdot A_{\left({cm}^{2}\right)}\cdot 10^{-6}}$$

where the numerator is the absolute value of total current or the anodic and cathodic currents combined (amps), ν is the scan rate, and *A* is the area of the working electrode.

*C*_dl_ measurements can be coupled with analyzing the Faradaic current during CV of a diffusional redox species or probe, such as potassium ferricyanide (K_3_Fe­(CN)_6_), denoted as FeCN throughout the text herein. The peak shape of FeCN voltammetry shifts from a reversible diffusional peak shape to a quasi- or irreversible peak shape as the FeCN becomes increasing blocked from accessing the electrode interface for oxidation/reduction.^55^ Prior to making these measurements, strict rinsing patterns were used in between voltammetry in different solvents, with water rinses always followed by rinses of the solution in which the measurement was to be made. SAMs from widely available thiol solutions (5 mM in ethanol) were allowed to sit overnight. XB donor ligands, either used to modify electrodes with SAMs or to functionalize C6MPCs through exchange reactions, included hexadecaperfluoro-8-iodooctane-1-thiol (**L1**) and 2,3,5,6-tetrafluoro-4-iodo-*N*-(4-mercaptophenyl)­benzamide or tetrafluoro-4-bromo-*N*-(4-mercaptophenyl)­benzamide (**L2** with I or Br termination). Both these ligands were synthesized and characterized as described in previous studies.^36,56^ SAMs of these thiol ligands were formed overnight by immersing the gold electrodes in 1 mg/mL solutions of L1 (ethanol) or L2 (methanol). After SAM formation, solutions of DABCO and 1-BP solutions (1 mM in toluene or cyclohexane, respectively) as XB acceptors, were allowed to equilibrate at the SAM interfaces in the electrochemical cells for 12 h prior to subsequent measurements. Importantly, XB acceptor solutions were removed from the electrochemical cell with careful attention not to directly impact films at 90° unless it was a polar solvent rinse being used to intentionally disrupt and diminish XB interactions. Strict rinsing protocols were used for each transition to a new solvent, including cell rinsing 5× with the existing solvent immediately followed by rinsing 5× with the solvent to be immediately used (no solutes in both cases) prior to exposure to a solution with a solute.

### 2.4 Gold Nanoparticle Synthesis, Functionalization, and Characterization

Hexanethiolate-protected monolayer-protected clusters (C6-MPCs) were synthesized from HAuCl_4_·3H_2_O as described previously, using a modified version of the two-phase Brust-Schriffin procedure that has been extensively reported in the literature.^57^ The resulting C6-MPCs were characterized with NMR, UV–vis and TEM (Supporting Information, Figures SI-2–SI-5) and, consistent with prior reports of a polydisperse sample showing an initial *average* diameter and composition of 2.54 (±0.64) nm and Au_140_(C6)_53_, respectively.^58^ For this study, that sample was further treated with established fractionation procedures to limit the polydispersity based on solubility in more polar solvent environments,^59^ eliminating the larger particles present in the sample through the process, resulting in a smaller average diameter and more narrow distribution of C6-MPCs of 2.15 (±0.47) nm (Supporting Information, Figures SI-6–SI-8). This material represents unfunctionalized MPCs (unf-MPCs) lacking the ability to engage in XB interactions. Unf-MPCs were converted to XB capable or functional-MPCs (f-MPCs) by vigorously stirring them in a THF solution with a stoichiometric amount of thiolated ligands for 5 days to promote well-established ligand exchange reactions.^60^ To preserve as much f-MPC material as possible, exchange reaction solutions were rotary-evaporated to dryness (Buchi, R-300), precipitated in acetonitrile, and centrifuged to decant the supernatant portion. The procedure was repeated twice to “wash” samples (i.e., remove unbound free thiol or disulfides). ^1^H NMR and ^19^F NMR both before and after I_2_-decomposition to liberate NP-bound ligands as disulfides was performed to characterize the chemical composition of the f-MPCs. In particular, ^19^F NMR was useful to confirm the presence of XB donor ligands on the f-MPCs (Supporting Information, Figures SI-9 and SI-10).^36^

MPC film assemblies at gold electrodes were formed using previously reported procedures.^54^ Briefly, clean gold film electrodes were modified with C6 thiol (5 mM in ethanol) base SAMs that were subsequently exchanged (hours) by adding an ethanolic solution of 5 mM undecanedithiol (UDDT) linking ligands for 15 min before rinsing and exposing the interface to a 1–2 mg/mL solution of unf-MPCs or f-MPCs (L1) or f-MPCs (L2- Br or I) in toluene. This cycle, which attaches MPC material to the SAMs via the dithiol linker, was then repeated at least twice to ensure high coverage of the interface with MPC material. Film assembly was followed as in previous studies by monitoring the *C*_dl_ during the formation of the films as described in the text and in prior studies.^54^

### 2.5 Conductivity Measurements on Interdigitated Array (IDA) Electrodes

Conductivity measurements were performed as previously by our lab^21^ and others.^22^ SWCNT (Nano Lab, Inc.) were mixed with f-MPC, unf-MPC, or dihalo-perfluorinated XB donor molecules (e.g., di-iodotetrafluorobenzene) in specific mass ratios and ball-milled or grinded at 1750 rpm for 5 min (SPEX SamplePrep 2010 Geno/Grinder). This material was then mechanically compressed (Carver Laboratory Press—Model C, Fred S. Carver INC; 5.5 tons for 1 min) into a pellet identified as a PENCIL (Process Enhanced NanoCarbon for Integrated Logic).^61^ Prior to being modified with the PENCIL, gold IDA electrodes (Metrohm, DRP-IDEAU200) were cleaned with immersion in 0.1 M H_2_SO_4_, rinsed with UP H_2_O, and dried in a stream of N_2_. The PENCIL material was then mechanically abraded across the IDA until a targeted film resistance in the range 0.36–1.36 kΩ (avg. = 0.925 kΩ) was achieved as measured via potentiostat-generated *I*–*V* curves (+0.1 to −0.1 V) connected to an IDA sample holder (Metrohm, DRP-CACIDE). Prior to vapor measurements the modified-IDAs were equilibrated in a low-level stream of N_2_ (overnight).

After overnight equilibration with N_2_, modified IDAs were mounted in a previously demonstrated, machine-shopped Teflon vapor cell mounted with IDA holder that allowed for potentiostatic control.^21^ The vapor cell is in line with a gas generating system, either the rudimentary system that bubbles N_2_ gas through solvent, previously demonstrated to deliver different ratios of chemical vapor (Supporting Information, Figure SI-11)^21^ or as part of a permeation tube system that delivers specific concentrations of chemical vapor across the sensing interface. A complete schematic of the latest, concentration-controlled instrumental apparatus for vapor measurements is shown in Supporting Information (Figure SI-12). Once installed in the vapor cell, amperometric (+0.1 V) current–time (*I*–*t*) measurements across the modified IDAs were collected. Prior to introducing chemical vapor, the system was allowed to re-equilibrate in N_2_ for ≥ 500 s to establish an initial, stable baseline. *I*–*t* curves were then monitored during successive cycles (4) of a specific concentration of chemical vapor (e.g., cyclohexanone), each exposure followed by a return to 100% N_2_. After chemical vapor exposure, the system was allowed to purge with 100% N_2_ (15 min). Amperometric *I*–*t* curves were generated with four successive exposures to chemical vapor of a particular vapor concentration, thereby giving four responses and four recovery phases. As in prior reports of this nature,^21,29^ baseline corrections were conducted using the average of three chemical vapor exposures at each type of film. Current responses were normalized using a conversion to conductance change (i.e., Δ*G*/*G*_o_%), calculated from the following equation applied to a response pulse in the *I*–*t* curve

3 $$\%\frac{\Delta G}{G_{0}}\;\mathrm{at}\;\mathrm{time}\;t=\frac{current\;at\;t-baseline\;i}{baseline}\times 100$$

## 3 Results and Discussion

### 3.1 Studying Interfacial Interactions Using Electrochemistry and Self-Assembled Monolayers

Self-assembled monolayers (SAMs) can be used to form an ordered interface with relatively knowable and controllable surface chemistry. As such, SAM-modified electrodes are often used in sensor development to study surface interactions.^62,63^ Two types of electrochemical measurements, double-layer capacitance (*C*_dl_) and redox probe cyclic voltammetry (CV), when used in conjunction with each other, have proven to be particularly instructive for studying interactions at SAM-modified electrode interfaces.^55,64^ *C*_dl_ measurements take advantage of the sensitivity of the electric double layer, as described by the Helmholtz model of electrode under potential control in an aqueous solution, to probe the physical and chemical properties of the interface between an electrode and an electrolyte solution.^64^ As such, anything that increases the distance and/or decreases the dielectric between the charge plates, such as an adlayer of interacting molecules, will notably decrease *C*_dl_. A decrease in *C*_dl_ is a common phenomenon observed upon alkanethiol modification of electrodes form SAMs.^55^

*C*_dl_ behavior of a SAM-modified interface is easily observed when comparing freshly cleaned bare gold before and after SAM-modification (Figure 1A). The results clearly show that modification of the bare gold with a self-assembled C6-hexanthiol or ω-substituted alkanethiolate ligands (e.g., 11-mercaptoundeconoic acid or MUA) causes a significant change in *C*_dl_. Any interfacial chemistry that increases interplate distance (↑d) or lowers the dielectric (↓ε) will result in lower current flow across the capacitor, an effect that manifests as a smaller voltammetry response.

**Figure 1:**
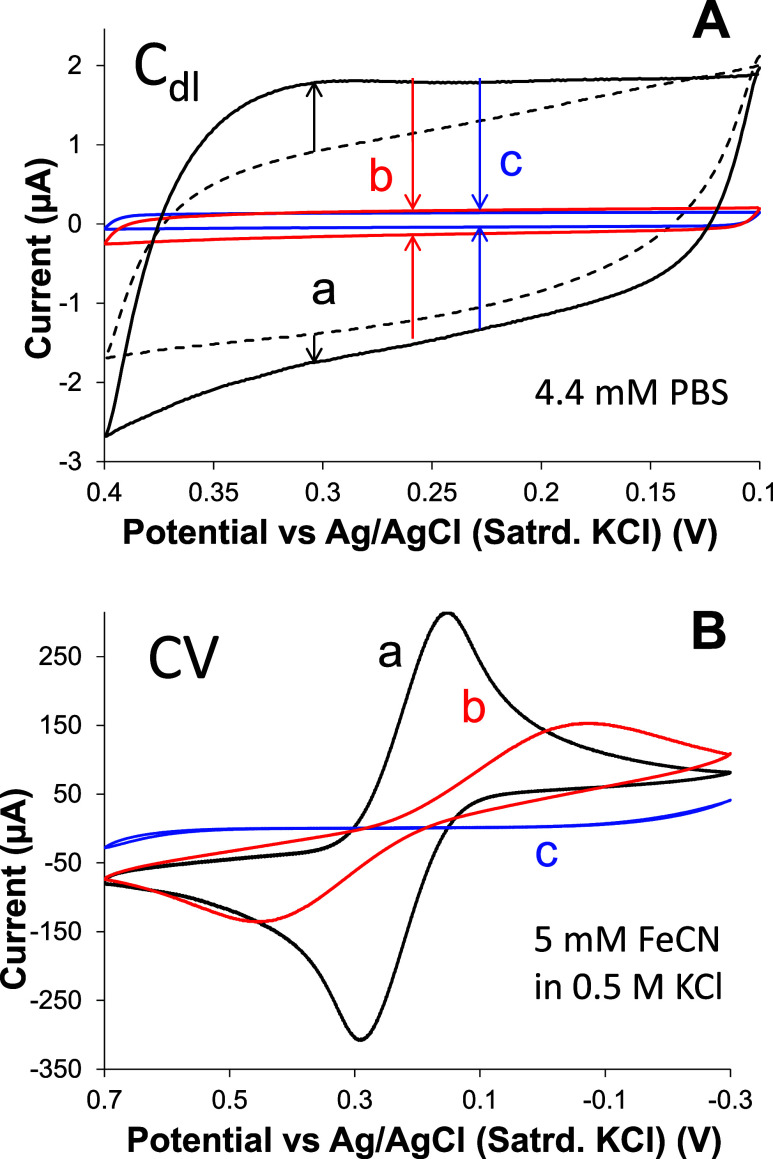
(A) Representative CV measuring change in *C*_dl_ (a) from as-received gold substrates (*dashed*) to electrochemically cleaned bare gold (*solid*); (b) clean bare gold to modified with a C6 SAM; and (c) clean bare gold to MUA SAM; (B) representative CV of FeCN at (a) clean/unmodified, (b) C6 SAM, and (c) MUA SAM modified gold. Note: 100 mV/s.

Another classic electrochemical method available to indicate the presence of an interfacial adsorbate layer, such as a SAM or an adsorbed species at a SAM, has been to observe the diffusional behavior (i.e., the Faradaic current) of a well-established solution redox probe molecule during cyclic voltammetry (CV). More specifically, the peak shape of the CV for a freely diffusing solution species with well-defined electrochemical behavior (e.g., potassium ferricyanide (FeCN), ruthenium hexamine, or hydroxymethyl ferrocene) measured at both bare and modified gold substrates is often coupled with *C*_dl_ measurements to confirm changes to electrode surfaces. As an electrode becomes more blocked, either with modification with a SAM or because of a layer of adsorbates forming at an established SAM interface, the normally reversible CV of such molecules will exhibit more quasi- to irreversible ET kinetics and attenuated peak currents.^55^ An example of such a result is shown in Figure 1B with FeCN voltammetry shown at a bare gold versus the MUA-modified gold substrate. In the experiments that follow, these two measurements will be used to investigate XB interactions at intentionally designed SAM interfaces.

### 3.2 Electrochemical Evidence of XB Interactions at SAM-modified Electrodes (Solution)

#### 3.2.1 Double Layer Capacitance Cyclic Voltammetry

As previously discussed, the orientation of a halogen bond is an important indicator of interaction strength as a linear XB (180°) angle optimally aligns the geometry of the interaction between the σ-hole of the halogen atom with the Lewis-base XB acceptor. Scheme 2A (*top*) shows two model molecules, established in prior studies as either moderate (1-BP)^56^ or strong (DABCO)^36,65,66^ XB acceptors, respectively. Similarly, Scheme 2A (*bottom*) shows two thiolated ligands that will serve as sensors, or XB donors. The perfluoro-alkanethiol and the perfluoro-aromatic amide thiol will be denoted as **L1** and **L2**, respectively. Both ligands were synthesized and characterized according to procedures reported elsewhere.^36,56^ As a straight chain perfluoro hydrocarbon, **L1** exhibits a significant σ-hole leading to strong XB interactions.^66^ Similarly, while **L2** represents a novel sensor, its design is based on previous research demonstrating that the incorporation of a tetrafluoro-diiodo substituted benzene moiety results in a responsive XB sensor.^21^ In **L2**, one of the iodine atoms has been replaced with an amide bond connecting to an aromatic thiol unit, which is used to anchor the **L2** sensor to gold substrates. These additions are expected to impart conformational rigidity while also encouraging electron flow/delocalization from the iodine to the surface. **L1** and **L2** have been confirmed to form SAMs on clean gold surfaces where the film formation subsequently lowers *C*_dl_ and exhibits blocking behavior toward solution FeCN voltammetry (Supporting Information, Figures SI-13– SI-15). As a first step toward SAM-modification of gold using these ligands, density functional theory (DFT) was used to determine the energetics of various conformations of **L1** and **L2** both in gas and liquid phases.

**Scheme 2:**
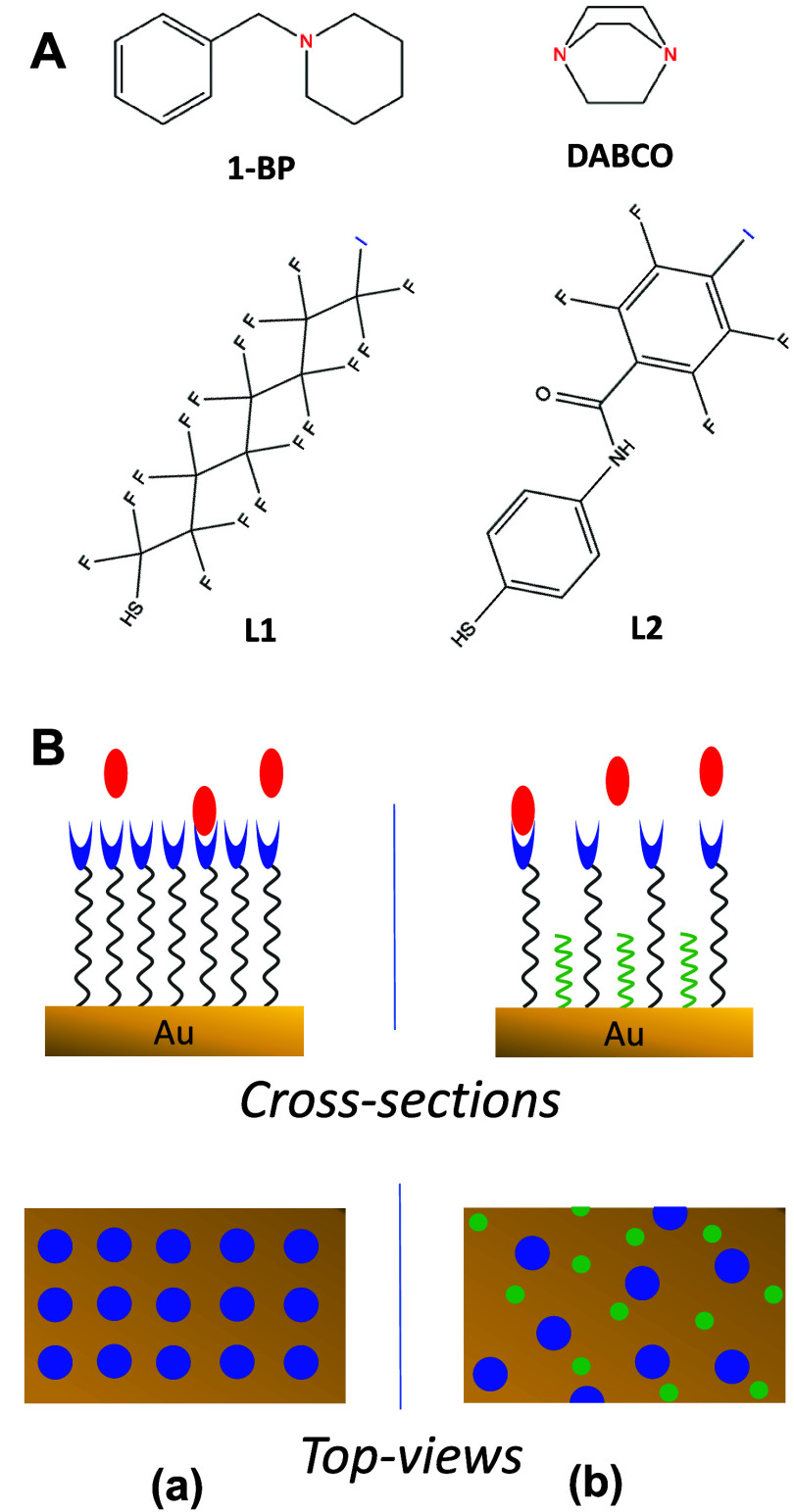
(A) 1-Benzylpiperidine (1-BP) and 1,4-Diazabicyclo[2.2.2] Octane (DABCO) Used as Moderate and Strong Model XB Acceptor Molecules, Respectively, and XB Donor Thiol Ligands to Be Studied (**L1, L2)**; (B) Cross-Sectional and Top-Views of (a) Uniform SAMs Comprised of L1 or L2 with XB Donor Moieties (Blue) and (b) Mixed SAMs of Either L1 or L2 with Hexanethiolate (C6) as a Diluent Interacting with XB Acceptors (red)^a^

a Some experiments featuring a brominated L2 molecule were also conducted.

#### 3.2.2 Conformational Behavior of **L1** and **L2** Determined by DFT

Conformational analysis of **L1** and **L2** using DFT was employed to help understand their behavior in solution, and as a rough approximation of how they might assemble on a surface, albeit in a low-density fashion. The analysis is not meant to probe **L1/L2** adsorption onto substrate, or even interaction between **L1/L2** molecules, but rather to determine if they are predisposed to a linear arrangement before incorporation into the SAM. Manual conformational analysis (Supporting Information, Figure SI-16) shows that **L1** consistently optimized to a linear or bent conformation regardless of the initial input structure, with the linear structure lying 1.61 and 1.35 kcal/mol lower in energy than the bent structure at the B3LYP/cc-pVDZ level of theory in the gas and solvent (water) phase, respectively. This suggests a natural tendency for the fluorine atoms to sterically repel each other, leading to a favoring of the extended, linear structure. **L2** adopted a single, extended conformation in both gas and solvent (water) environments regardless of the input structure. This is due to the trans orientation of the amide bond in the center of the molecule, along with the planarity of the two aromatic rings. Given the difference in energy for the **L1** extended and bent structures, we would expect that the percent composition of an ensemble of **L1** at room temperature would be roughly 94:6 extended versus bent. These results are consistent with experimental evidence that SAM-based ligands typically adopt extended, linear conformations, especially when there are multiple ligands bound to a gold substrate.^67,68^

#### 3.2.3 SAM Formation Using **L1** and **L2**

Based on our DFT results, we are confident that SAM interfaces could be formed of either uniform **L1** or **L2** (Scheme 2B, *left*) as well as mixed SAMs comprised of a hexanethiolate (C6) base (non-XB donor) subsequently modified with either ligand (**L1** or **L2**) (Scheme 2B, *right*). Importantly, while *C*_dl_ confirms the initial formation of these uniform SAMs, the creation of the mixed SAM could also be confirmed with small increases in *C*_dl_ as a function of time when exposed to solutions of the fluorinated ligands, a signal consistent with their exchange into the C6 SAM adlayer (Supporting Information, Figures SI-17 and SI-18). Additional evidence of a mixed SAM was gained from linear sweep voltammetric (LSV) desorption of films. While not quantitative, LSV of uniform SAMs showed a singular peak of reductive desorption while mixed SAMs exhibited multiple peaks (Figure SI-19). As depicted in the overhead views of these two different SAM structures (Scheme 2B, *bottom*), the extended ligand structure coupled with the repulsive and polarized nature of the fluorination works to increase the dielectric of the film and/or lower its ordered structure, thereby causing slight increases in the film’s *C*_dl_ as ligand-exchange occurs.^68^ For the purposes of our study, this observation simply serves as an effective indicator that fluorinated ligands, either **L1** or **L2**, are being incorporated into the film to form mixed SAM interfaces. These uniform and mixed SAMs, as well as non-XB control SAMs (e.g., MUA SAMs), represent our starting platforms or based systems for studying XB interactions with model XB acceptors in aqueous solutions.

Each SAM, both uniform (100%) **L1** or **L2** as well as mixed SAMs, either **C6/L1** or **C6/L2**, were all exposed to solutions of 0.5 mM DABCO, an established, strong XB acceptor molecule. Figure 2 shows CV-measured *C*_dl_ measurements on all four types of SAM films before and after DABCO exposure. Given the previously published experimental and computational data suggesting the strength of DABCO as an XB acceptor as well as the strength of iodine substituted perfluoro-aromatics (e.g., iodopentafluorobenzene or IPFB), it is somewhat surprising that uniform SAMs comprised of either **L1** (Figure 2A) and **L2** (Figure 2B), after significant exposure to DABCO in solution, did not display a more robust decrease in film *C*_dl_. On the other hand, after the same DABCO exposure at the mixed SAMs (i.e., C6/L1 and C6/L2), the effect is significantly more pronounced (Figure 2C,D) with *C*_dl_ decreasing upon exposure to the strong XB acceptor even though the number of XB donor sites is diluted with C6-alkanethiolate spacers (Scheme 2B). In the case of the mixed SAMs (C6/L1 and C6/L2), it was established that the interfacial XB interactions increased with time as *C*_dl_ systematically decreases after 2–3, 6, and 12 h of DABCO exposure (Supporting Information, Figures SI-20 and SI-21) before the effect plateaus.

**Figure 2:**
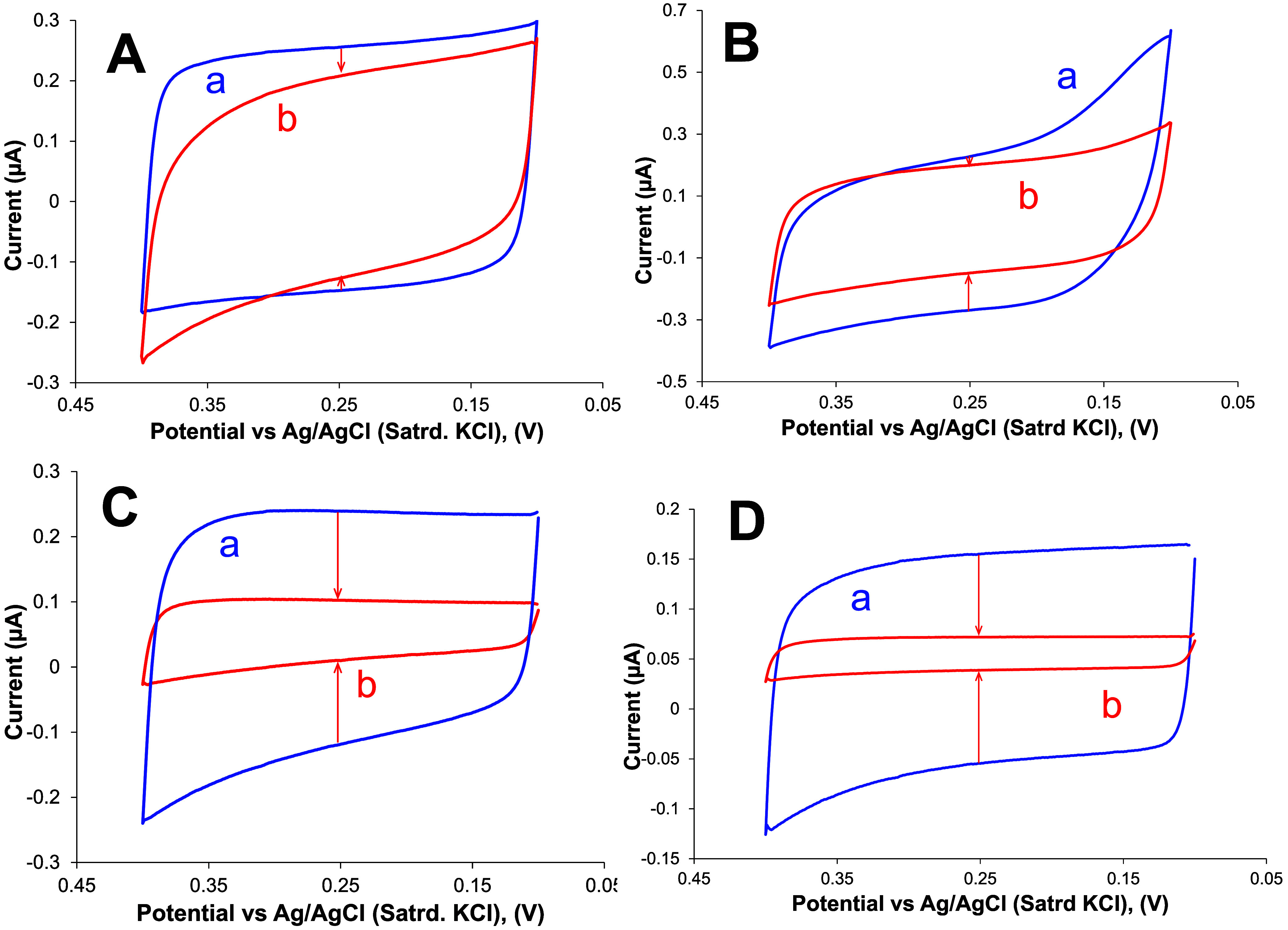
Representative CV in 4.4 mM PBS reflecting *C*_dl_ of gold electrodes modified with (A) a uniform L1 SAM, (B) a uniform L2 SAM, (C) a mixed C6/L1 SAM, and (D) a mixed C6/L2 SAM both (a) before and (b) after exposure to the XB acceptor DABCO (12 h).

Several control experiments were conducted to increase confidence that the observed *C*_dl_ decreases were because of XB interactions at the SAM interfaces. Specifically, a control film without XB donor capability was formed using 11-mercaptundecanoic acid (MUA)-exchanged into a C6 SAM (i.e., MUA/C6 mixed SAM). In that case, regardless of the length of exposure to DABCO, the voltammetry showed the *C*_dl_ of the films largely unchanged before and after exposure (Supporting Information, Figure SI-22). Additionally, given that XB is largely electrostatic in nature and its strength often solvent dependent,^38,66,69,70^ it was hypothesized that vigorous washings of the DABCO adsorbate layer with highly polar solvents (e.g., ethanol, isopropyl alcohol) followed by water would easily disrupt and remove any XB molecules at the interface. In all such experiments, with some example results provided in Supporting Information (Figures SI-20 and SI-21), the voltammetry after that rinse was nearly identical to that prior to DABCO exposure suggesting that the adsorbate layer is effectively removed.

A large set of analogous experiments to those just described were conducted on the same SAMs (uniform and mixed) with exposure to 1-BP, a more moderate strength XB acceptor.^56^ All of the results from these experiments (Figure SI-23) showed the same trends as observed with DABCO shown in Figure 2 (above) where exposure to the XB acceptor resulted in larger *C*_dl_ decreases on the mixed SAMs versus the changes observed on uniform SAMs. There was also a similar time-dependence observed and, as before, the system returned to its pre-exposure voltammetry with the polar solvents. Control experiments on non-XB capable mixed SAMs showed virtually no change in *C*_dl_ with any 1-BP exposures (Figure SI-24). From the collective *C*_dl_ results across all these SAM interfaces interacting with these two XB acceptors, it was evident that 1-BP consistently behaved as a less potent XB acceptor molecule, an observation consistent with other literature reports.^56^ Numerical measurements of *C*_dl_ for the various systems, are available in the Supporting Information (Table SI-0).

#### 3.2.4 Redox Probe Solution Voltammetry of Ferricyanide

As mentioned in Section 3.1, *C*_dl_ decreases accompanied by corresponding changes in FeCN diffusing voltammetry, represents strong evidence of adsorbates interacting with the sensor interface via intermolecular interactions such as XB. In our SAM interfaces, each *C*_dl_ decrease observed upon exposure to XB acceptors showed a corresponding increase in FeCN being blocked at the film interfaces. Figure 3 shows representative results of FeCN voltammetry at bare gold versus that of a uniform **L1** SAM and a **C6/L1** SAM, both before and after exposure to DABCO (Figure 3A). The interaction of DABCO with the sensor via XB results in increased blocking of the FeCN in solution and increases the irreversibility of peak shape in the observed voltammetry. Similarly, a comparison of FeCN voltammetry at bare gold versus a uniform **L2** SAM and a mixed **C6/L2** SAM after exposure to DABCO (Figure 3B) reinforces *C*_dl_ findings where there seems to be a greater XB interaction at the mixed SAM interfaces. Here again, the collective FeCN voltammetry, coupled with the *C*_dl_ voltammetry, suggested that DABCO behaves as a strong XB acceptor at the mixed SAM interfaces.

**Figure 3:**
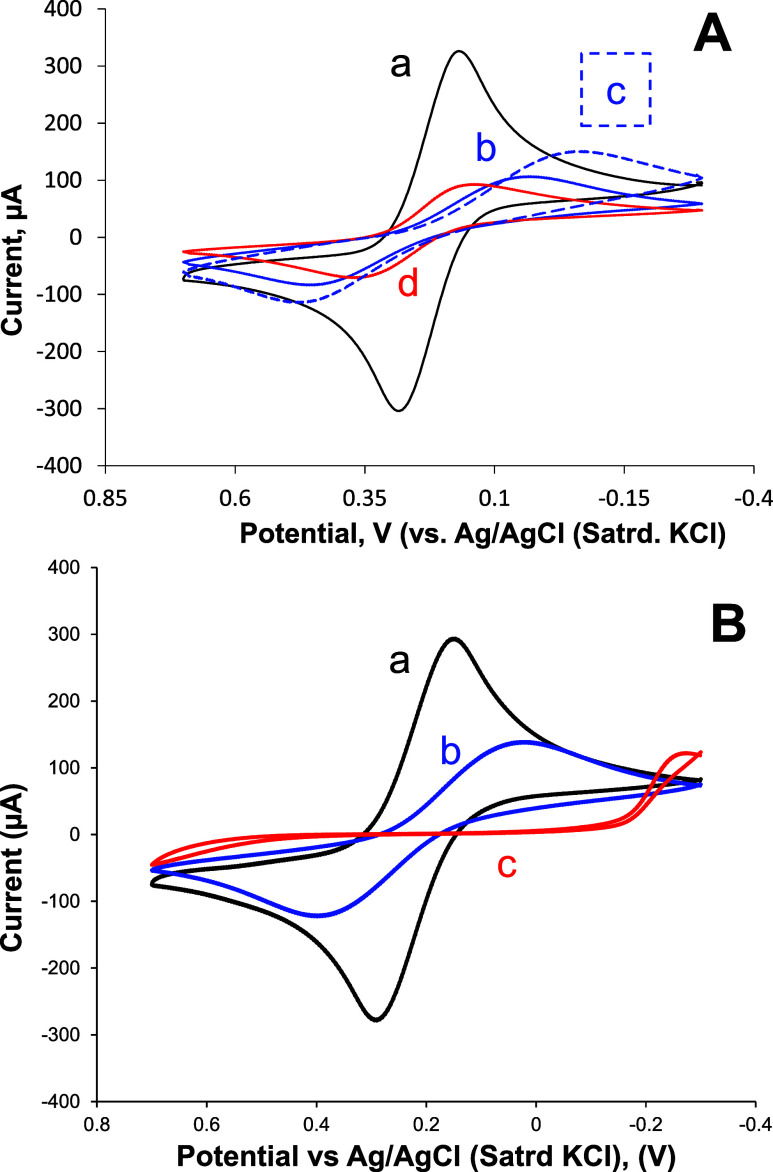
Representative FeCN CVs with overlaying scans: (A) (a) bare/clean gold electrode, (b) L1 SAM and (c) C6/L1 mixed SAM before and (d) after exposure to DABCO and; (B) (a) bare/clean gold and (b) L2 SAM and (c) C6/L2 mixed SAM after both films are exposed to DABCO. Note: CV is in 5 mM K_3_Fe­(CN)_6_ in 0.5 M KCl (100 mV/s).

Figure 4 illustrates similar results showing that FeCN voltammetry is increasingly blocked at both **L1** uniform and **C6/L1** mixed SAMs after exposure to the more moderate XB acceptor 1-BP (Figure 4A). If that result is contrasted with the same experiments of 1-BP at either a uniform **L2** SAM or a mixed **C6/L2** SAM (Figure 4B), it suggests that **L2** is the more potent XB donor ligand and can interact better even with a moderate strength XB acceptor molecule like 1-BP. The seemingly stronger XB interactions of both acceptors at either mixed SAM (**C6/L1** or **C6/L2**) versus their uniform, single-ligand SAM, suggests that the mixed SAM interface allows for more optimal XB donor functionality. As such, it was hypothesized that the mixed SAMs, by presenting protruding moieties offering XB donor functionality out into solution allow for more favorable interaction geometries and stronger XB interactions. In contrast, within uniform SAMs that do not contain C6 diluent, the same XB donor moieties are more sterically constrained and, despite likely having more moieties present (vs mixed SAMs), align less favorably with the σ-holes. To test this hypothesis, film assemblies of metallic NPs were employed as surface modifiers to further optimize the presentation of XB donor ligands and allow for XB orientations consistent with strong intermolecular interactions.

**Figure 4:**
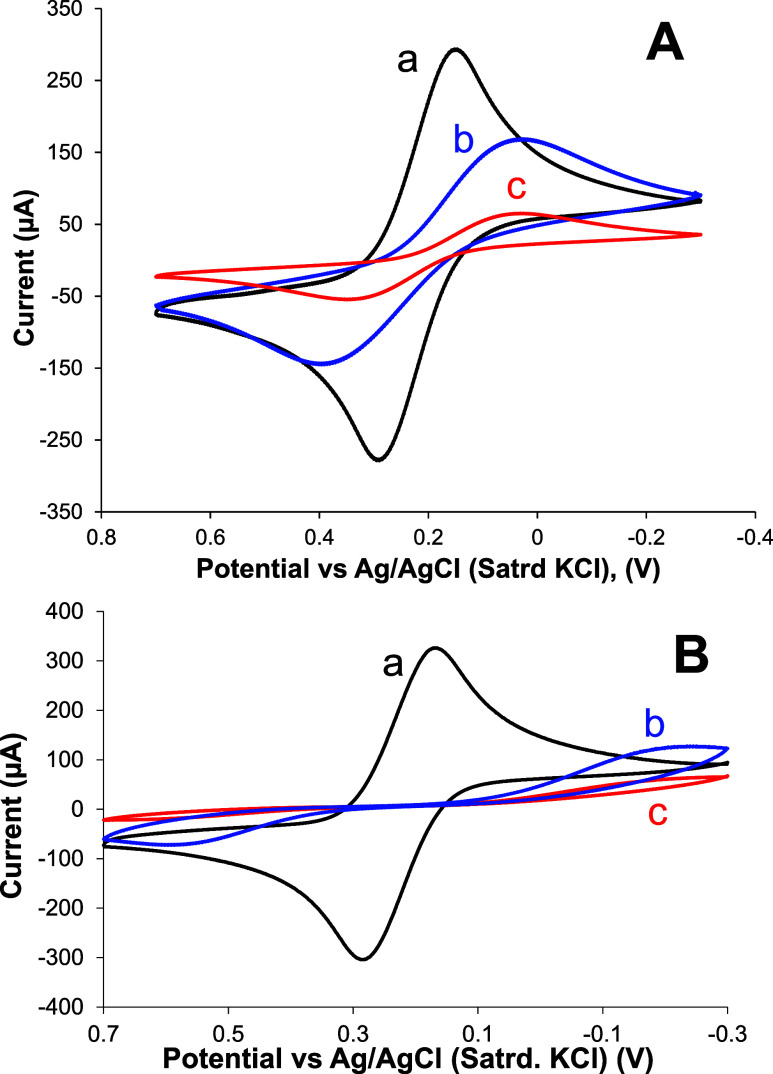
Representative FeCN CV scans at (A) (a) bare/clean gold, (b) L1 SAM and (c) C6/L1 mixed SAM after both films are exposed to 1-BP; and (B) (a) bare/clean gold and (b) L2 SAM and (c) C6/L2 mixed SAM after both films are exposed to 1-BP. Notes: 5 mM K_3_Fe­(CN)_6_ in 0.5 M KCl (100 mV/s).

### 3.3 Electrochemical Evidence of XB Interactions at NP Film-Modified Electrodes in Solution

For over two decades, alkanethiolate-protected gold NPs have been explored in both solution and as assembled films.^71^ Appropriately called monolayer-protected clusters (MPCs), these NPs are essentially 3-D SAMs that feature high stability, controllable core sizes, and the ability to systematically alter the peripheral ligand length and functionality via well-established ligand exchange reactions (Scheme 3A) to form functionalized MPCs (f-MPCs).^36^ For this study, C6-MPCs were synthesized and subsequently functionalized with the XB donor ligands (Scheme 2A) forming functionalized MPCs (f-MPCs) that were capable of more optimal interactions with XB acceptors. NMR analysis was used to confirm the presence of XB-donor ligands in the f-MPCs (see Section 2). These NPs were assembled into a film at a C6 SAM interface (Scheme 3B), where successful layering of MPC material at the SAM base resulted in small but observable increases in C_dl_ with each exposure to MPCs, and simultaneous increased FeCN blocking compared to the base C6 SAM prior to attaching the MPC film (Supporting Information, Figures SI-25–SI-27).^54^ Additionally, the formation of the MPC film assemblies can be visually verified in that, once the gold substrates are removed from the electrochemical cells, the extremely high molar absorptivity of MPCs results in clearly visible thin films (Scheme 3C). These MPC thin films essentially present a mixed SAM interface with greater surface area that projects more optimal XB donor sites for incoming XB acceptors (Scheme 3B). As such, significant and electrochemically detectable XB interactions were hypothesized at those interfaces.

**Scheme 3:**
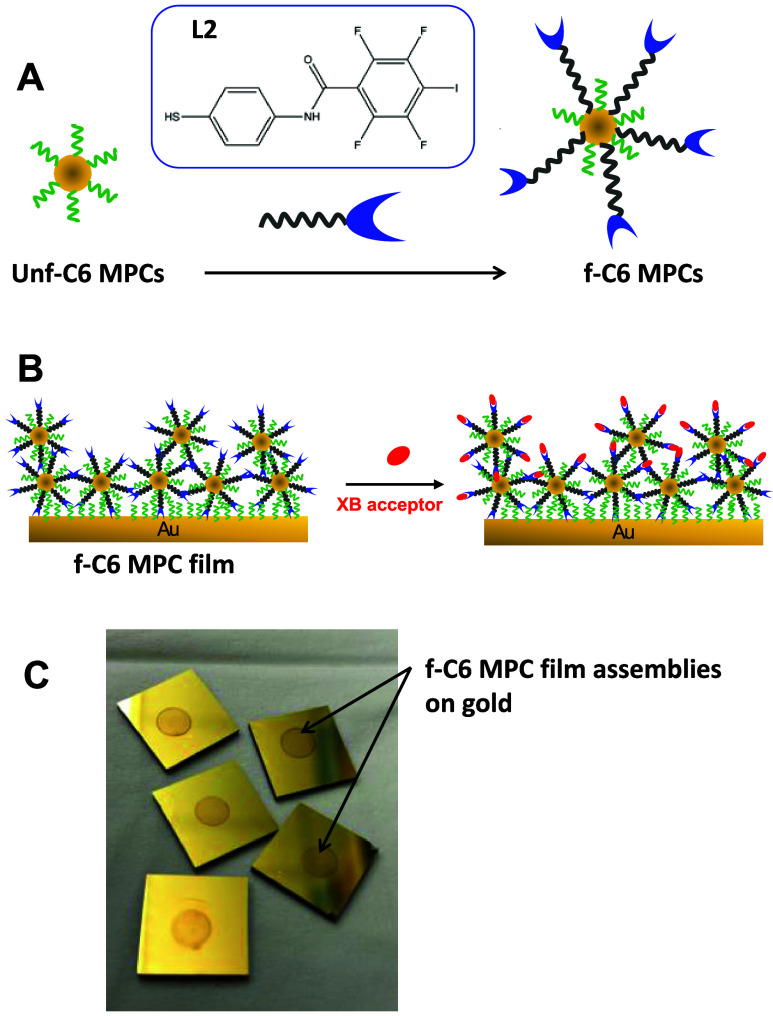
Schematic Representations of (A) MPC Exchange Reaction That Forms f-MPCs That Then are Used for (B) Constructing a f-MPC Film Assembly That Can Then Be Exposed to XB Acceptors; and (C) Photograph of MPC Films after Use in Electrochemical Cells^a^

a Photograph courtesy of Michael C. Leopold. Copyright 2025.

Films assembled from f-MPCs featuring **L2** XB donor ligands were grown on gold substrates and exposed to DABCO in the same manner as for the SAM-modified electrode experiments described earlier. Once formed, the *C*_dl_ and FeCN voltammetry of the films prior to and after exposure to XB acceptor molecules were key observations. Figure 5A shows representative results of the first set of experiments using films of f-MPCs featuring the **L2** ligand. The FeCN voltammetry at bare gold, a reversible diffusional wave-shape, is clearly diminished with the subsequent modifications of C6 SAM followed by f-MPC film assembly. With exposure to DABCO, the voltammetry then becomes even more irreversible and blocked with a corresponding decrease in *C*_dl_ (Figure 5A, inset). These coupled results are indicative of a strongly interacting adlayer present at the f-MPC interface. As a control, analogous experiments with electrode modified with unfunctionalized C6MPCs (unf-MPCs), incapable of engaging in XB interactions as they lack any XB donor moieties, were conducted. Here again, the results (Figure 5B) at these films show the same blocking behavior after C6 SAM and unf-MPC film modifications. In this case, however, both the FeCN and *C*_dl_ voltammetry (Figure 5B, inset) show no significant change before and after exposure to DABCO. This result is in stark contrast to that of the f-MPC film response to DABCO. As with the SAM systems, f-MPC films exhibited the largest changes with DABCO exposure followed by more moderate changes with 1-BP and insignificant changes with control films of unf-MPCs exposed to those compounds (Supporting Information, Figures SI-28–SI-30).

**Figure 5:**
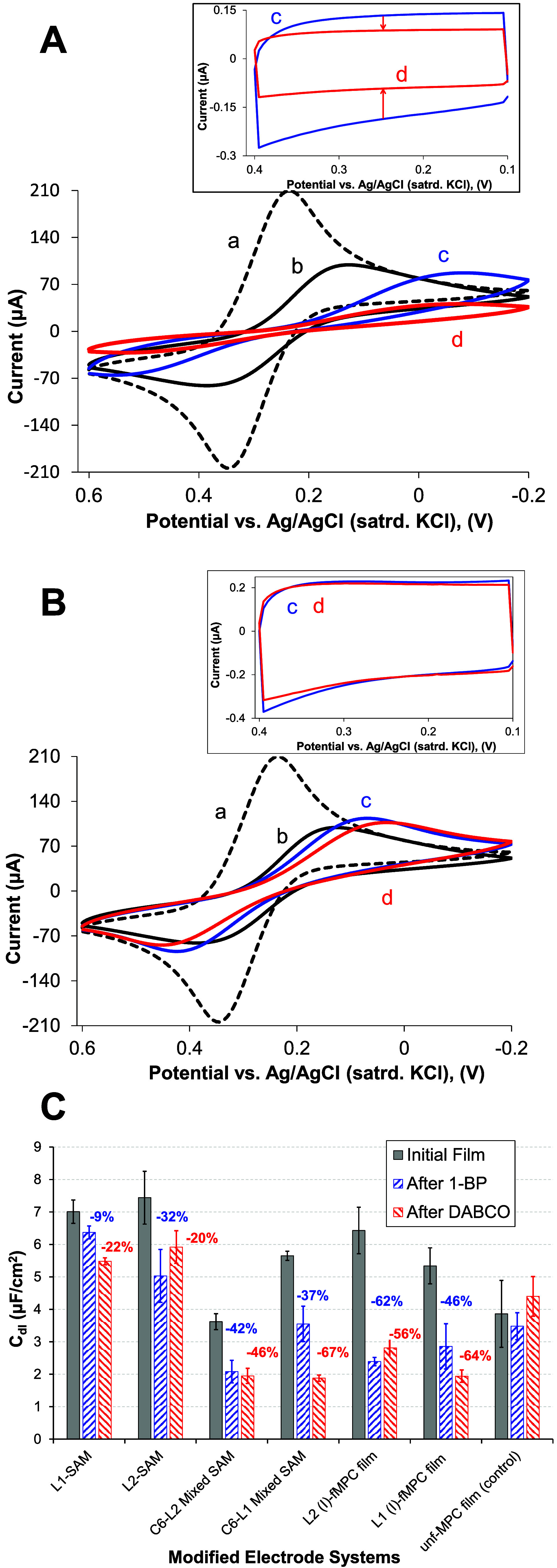
Representative FeCN CV for (A) f-MPC (L2-I) and (B) unf-MPC film systems including scans at (a) bare/clean gold (b) C6 SAM and MPC films (c) before and (d) after exposure to DABCO with corresponding *C*_dl_ voltammetry (insets: c and d) included for both systems; (C) summary of *C*_dl_ measurements across all interfaces exposed to both XB acceptors with the % decrease in capacitance shown (*n* = 3–7 with uncertainty is represented by standard error). Notes: 5 mM K_4_Fe­(CN)_6_ in 0.5 M KCl (100 mV/s); capacitance scans in 4.4 mM PBS; pH 7 (100 mV/s); *C*_dl_ scans of unmodified gold and C6 SAM not included to highlight *C*_dl_ changes due to DABCO. A numerical comparison of *C*_dl_ values from Figure 6C in tabular form is provided in Supporting Information (Table SI-1).

A summary of our *C*_dl_ results across various modified electrodes is shown in Figure 5C, including the *average* percent decrease in *C*_dl_ after exposure to each of the XB acceptor molecules (DABCO and 1-BP). Several interesting trends emerge from this data. When XB interactions occur at the film interfaces (SAMs or f-MPC films), the more notable decreases in *C*_dl_, regardless of the interface, were observed after exposure to DABCO as compared to 1-BP. For example, DABCO exposure at uniform SAMs of **L1** or **L2** resulted in ∼20% decreases in *C*_dl_ while the same ligands incorporated into mixed SAMs more than doubled that decrease: 46% and 67% decreases at **C6/L2** and **C6/L1** mixed SAMs, respectively. When uniform SAMs of **L1** and **L2** as well as mixed SAMs (**C6/L2** and **C6/L1**), were exposed to 1-BP, the majority of the results suggested **L2** was more effective at XB interaction with 1-BP. More specifically, the **L2** SAM decreased *C*_dl_ upon 1-BP binding by a third, while the **L1** SAM yielded a < 10% decrease on average. These findings are consistent with prior work establishing DABCO as a very potent XB acceptor^65^ and 1-BP as a more moderate XB acceptor.^56^ Likewise, the potency of **L2** as a more effective XB donor moiety was supported by f-MPC films with **L2** exhibiting ∼60% decreases for both 1-BP and DABCO (Figure 5C). The use of mixed SAMs and f-MPC films seemingly enhanced the strength of the interactions. No such definitive trends were observed for the control films comprised of unf-MPC films which are incapable of specific XB interactions.

Experimental evidence of significant XB interaction in solution, especially measured via electrochemistry, is a rare finding as it is generally accepted that XB interaction will be diminished by the presence of solvent.^38,70,72^ The consistency and strength of our solution phase results led us to consider whether the same XB donor and acceptor moieties, incorporated into our optimized interface, could be used for solvent-free, gas-phase sensor development and detection schemes where, in theory, they should have an even more pronounced effect.

### 3.4 XB Interactions Determined by DFT (Gas and Solvent Phase)

To better understand the atomistic underpinnings of the interactions between our XB acceptors and donors, we used density functional theory (DFT) to obtain optimized geometries for complexes of **L2** with the analytes shown in Schemes 1 and 2. We focused on the **L2** sensor as it showed the most promise during experimental testing. More details regarding the computational methods can be found in the Supporting Information.

In all cases, the energy of interaction between **L2** and the various analytes is negative, indicating a favorable interaction, i.e., the dimer is more stable than the isolated monomers. The analytes bind to the iodine atom on **L2** with bond lengths that are shorter than the sum of the individual atom VDW radii, and with R–X···B angles close to linear (180 °). A representative dimer between **L2** and cyclohexanone is shown in Figure 6 along with the electrostatic surface map. The strongest XB acceptor is DABCO, followed by 1-BP, with *E*_int_ of −10.0 and −8.7 kcal/mol, respectively. Across all analytes, the *E*_int_ ranges from −3.8 (RDX) to −10.0 (DABCO) kcal/mol. This suggests that **L2** will be effective at forming XB complexes with these analytes (Table 1). In our computational analysis the cyclohexanone analyte demonstrated the third highest affinity for L2 (−6.7 kcal/mol) along with the shortest X···B distance and most linear R–X···B angle. The lone pairs of electrons and the sterically unhindered ketone moiety make cyclohexanone an attractive analyte for experimental work (Figure 6).

**Figure 6:**
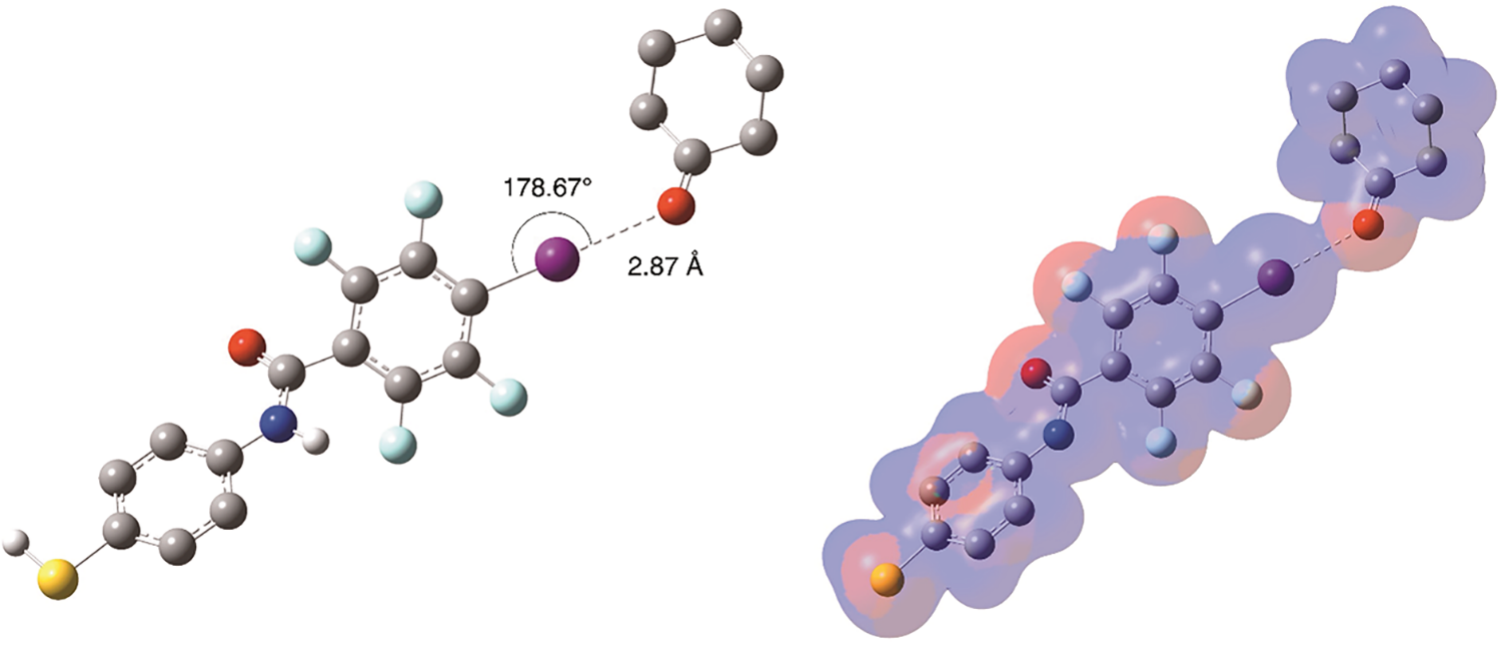
Left: M06–2X/cc-pVDZ geometry optimized halogen bonded dimer formed between L2 and cyclohexanone. Optimized structures, interaction energies, bond distances, and bond angles of XB adducts for all complexes can be found in the Supporting Information. (Right) ESP map of the L2 cyclohexanone dimer embedded on a 0.01 au electron density isosurface with values ranging ± 2.0 × 10^–5^ a.u. (red/blue). Structures and ESPs for all analytes can be found in Supporting Information (Figures SI-31–SI-38).

**Table 1:** Gas-Phase M06-2x/cc-pVTZ//M06-2x/cc-pVDZ Interaction Energies (Δ*E*_int_), Bond Distances (XBD), and Bond Angles of XB Adducts, for Comparison, the Sum of the van der Waal’s Radii for Iodine and Oxygen is 3.5 Å^a^ ^,^ ^c^

XB donors	Δ*E*_int_ (kcal/mol)^b^	X–B distance (Å)	R–X···B angle (θ)
1-BP	–8.72	2.84	179.09
DABCO	–10.00	2.77	179.77
DMDNB	–5.16	2.97	171.77
RDX	–3.76	3.02	174.79
TNT site1	–4.03	3.03	175.10
TNT Site2	–4.10	3.02	175.33
Cyclohexanone	–6.68	2.87	179.30

a (Iodine radius_VDW_ + Oxygen radius_VDW_).

b Δ*E*_int_ = E­(complex)–[E­(XB donor) + E­(XB acceptor)].

c For solvent-phase data see Table SI-2.

### 3.5 Gas Phase Measurements of XB Interactions with Optimized Interfaces

The DFT identification of CH as a moderately strong XB acceptor molecule that is also a target for explosive detection sensors was intriguing.^21,31^ The findings were reminiscent of the 2019 study by Jaini et al. that utilized interdigitated array (IDA) electrodes modified with single-walled carbon nanotubes (SWCNTs) that were functionalized with dihalo-tetrafluoro benzene selector molecules to detect CH (Scheme 4A,B).^21^ Results from studies of this nature,^21,29,33^ suggest that in that particular sensor design, the dihalo-tetrafluoro benzene selectors likely align parallel to the SWCNT. Such a configuration may be preventing the optimal orientation of the sensor with the XB acceptor (i.e., CH) and therefore not allowing for the strongest XB interactions. Given the current study’s results, it inspired a reexamination of that sensor design with the hypothesis that more optimal surface geometry for strong XB interactions may be achievable using a f-MPC film (Scheme 4C).

**Scheme 4:**
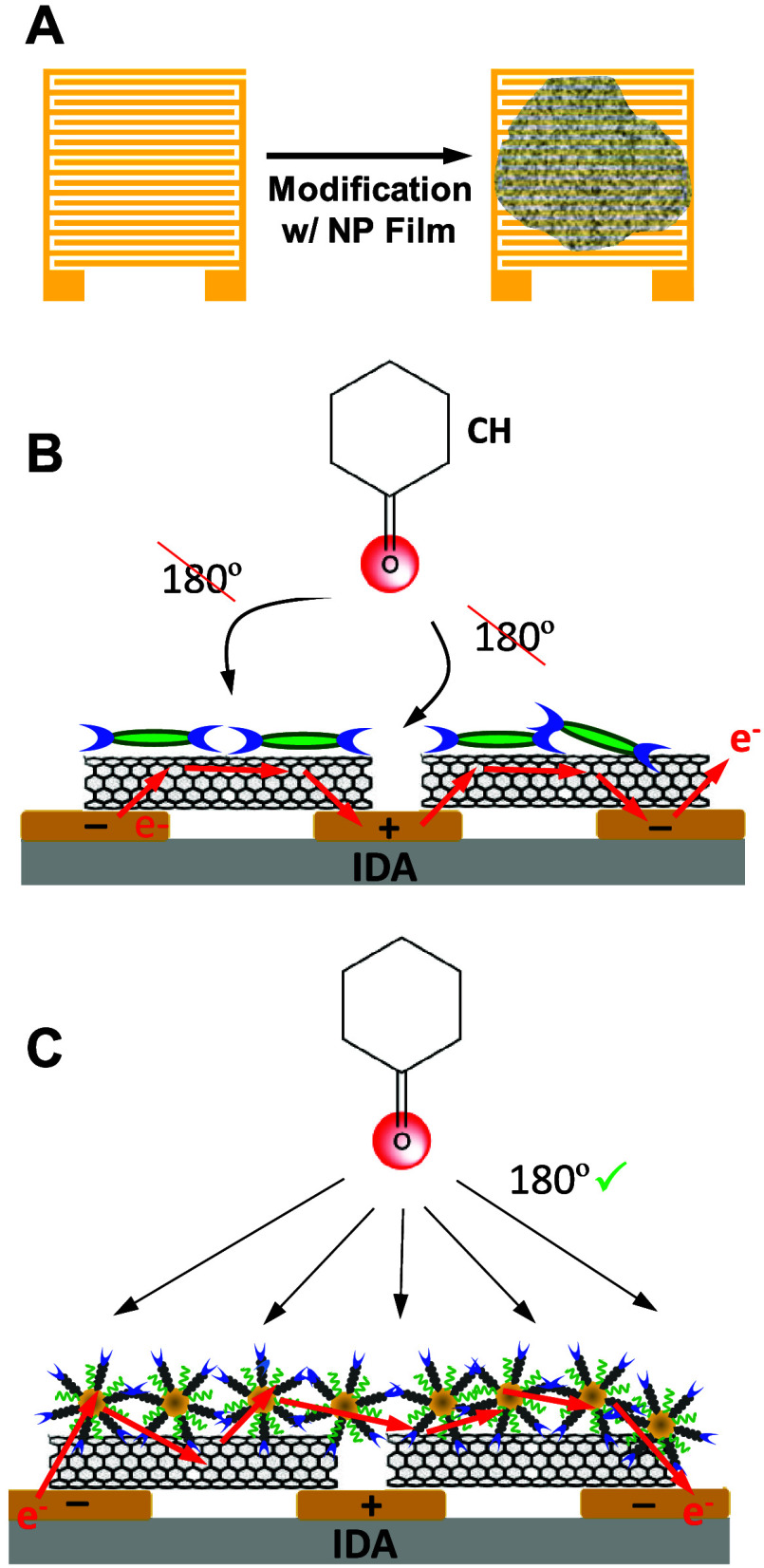
(A) Schematic Representations of Modifying IDA Electrodes with MPC Films for CH Vapor Experiments; (B) Illustration of Prior Work with IDAs Modified with SWCNTs and Di-halo-tetrafluoro Benzene Selector Molecules That May Have Restricted Angles for XB Interactions and; (C) Proposed Scheme of SWCNT Coupled with f-MPC for Improved Detection of CH Vapor via More Optimal XB Angles

To test the hypothesis, IDA electrodes were first modified with films comprised of SWCNTs and f-MPCs featuring the **L2** XB donor ligand terminated with Br, known to have a significant σ-hole. Examples of typical SEM imaging of the clean and modified IDAs are provided in Supporting Information (Figure SI-39). Control films of SWCNTs with unf-MPCs, lacking the ability to act as XB donors, were also created and both types of films were subjected to exposure to CH vapor (50%) in N_2_ while monitoring film conductivity. The films deposited onto the IDAs were optimized in terms of their MPCs to SWCNTs composition ratio by measuring the response of various combinations of materials to 50% CH vapor to determine that a 2:1 ratio yielded the most sensitivity toward CH (Supporting Information, Figure SI-40). Once optimized, the f-MPC:SWCNT and the control unf-MPC:SWCNT films were compared to the results of a prior study where SWCNT were modified with dihalo-tetrafluorobenzene XB donors with two halogens per molecule. The most sensitive response toward CH vapor in that study was SWCNTs with 1,4-di-iodotetrafluorobenzene.^21^ However, it was suspected that, because these molecules were likely aligned with the shaft of the SWCNT (π–π interactions), interacting XB acceptors like CH would be challenged to achieve optimal XB bond angle (Scheme 4B). Figure 7A illustrates the striking results of overlaying current response to 50% CH vapor at films of SWCNTs with f-MPCs (**L2**-Br), control films of unf-MPC:SWCNTs, films with 1,4-di-iodotetrafluorobenzene:SWCNT as well as SWCNT-only control films. From these results, the f-MPC (**L2**-Br)/SWCNT films produce nearly triple the response to CH vapor and notably outperform the most sensitive film identified in the prior study.^18^ The enhanced response cannot be attributed to increases in surface area provided by the MPCs because the control film incorporating unf-MPCs, incapable of XB interactions, yielded significantly lower responses. Additionally, conservative estimates of response/recovery time, where 95% of the signal is achieved and 95% of the signal returns to baseline, respectively, also suggest XB interactions are present. While all films showed relatively fast response to the 50% CH vapor, estimated to be hundreds of ppm, the f-MPC with the **L2**-Br, showed an extended recovery time. These recovery times are consistent with a significant concentration CH being adsorbed at the film via an intermolecular interaction.

**Figure 7:**
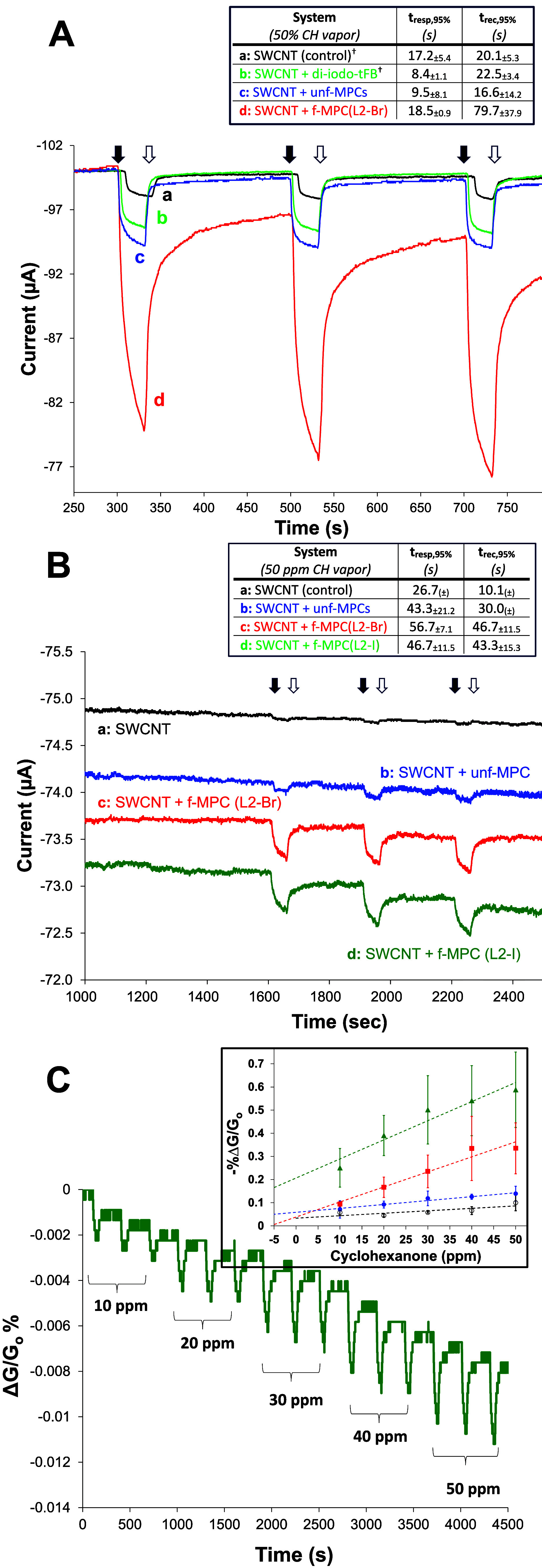
(A) Amperometric *I*–*t* curves showing current response of IDA electrodes modified with (a) SWCNTs films (control) and 2:1 mass ratio films of (b) 1,4-di-iodotetrafluorobenzene:SWCNT, (c) unf-MPCs:SWCNT,† and (d) f-MPC (L2-Br):SWCNT during three consecutive cycles of 50% CH vapor (black arrows) and N_2_ (white arrows) with measured film conductance and response/recovery time analysis (inset) for each type of film (*n* = 3–5); (B) *I*–*t* curves showing similar measurements at various modified IDAs during three successive exposures to 50 ppm of CH vapor (traces offset +0.5 μA for clarity) and response/recovery time analysis for each type of film (inset) and; (C) Example of sensing response of normalized conductance change [Δ*G*/*G*_o_ %] for CH vapor at various concentrations at f-MPCs (L2-I) and corresponding calibration curves (inset) for various films (*n* = 3–5). Note: †Results in (A), traces (a–c), from ref 23

The results prompted further experimentation with **L2**-modified MPCs and SWCNTs being exposed to controlled and known amounts of CH vapor delivered via permeation tube technology (see Section 2). Literature reports targeting CH detection suggest that its vapor range from 50 ppm to as high as 470 ppm for detection.^30,32^ An emergent goal of this work was to establish if the use of our f-MPC in this capacity could achieve a lower the limit of detection (LOD) by simply optimizing the geometry of the XB bond interactions using the NM composite materials. I-t curves, shown in Figure 7B, suggest that exposure of f-MPC:SWCNT featuring either **L2-Br** or **L2-I** functionalization to 50 ppm of CH vapor results in current responses significantly more pronounced than at control films (i.e., SWCNT only and unf-MPC:SWCNTs). Corresponding normalized conductance (Δ*G*/*G*_o_%) responses, provided in Supporting Information (Figure SI-41), show the same trends. Given that past studies suggest that the Br and I σ-holes on these ligands are comparable in size,^21,38^ it is not unexpected that their responses would be similar, especially if the bond angle is indeed being optimized. Additionally, while not as stark as the higher concentration results, response and recovery time analyses show an expected longer response time at low concentrations with sluggish recovery times consistent with an interfacial, intermolecular interaction being involved.^23^

The quantitative nature of the responses toward CH inspired examination of these films as sensing schemes. As such, the amperometric responses at both the control films and the f-MPC films assembled on IDA electrodes and exposed to increasing concentrations of CH vapor were collected. An example of this type of result is provided in Figure 7C showing current responses as a function of increasing CH vapor concentration at an IDA modified with SWCNTs and f-MPC­(L2-I). Typical examples of amperometric *I*–*t* curves and corresponding conductance (Δ*G*/*G*_o_%) responses for each type of film toward increasing amounts of CH are provided in Supporting Information (Figures SI-42–SI-46). These responses, averaged over several IDAs modified with each type of film, were translated into calibration curves for CH (Figure 7C, inset) and suggest, as with the higher concentration CH vapor testing (Figure 7A), that the films expected to engage in significant XB interactions systemically resulted in greater CH sensitivity (i.e., slopes of calibration curves). Specifically, films incorporating the f-MPCs featuring either **L2-Br** or **L2-I** functionalization exhibited enhanced CH sensitivity. The similarity in these results suggests that optimizing XB interaction geometries may negate the small theoretical difference in the σ-holes of these halogens.^38^ From the calibration curves, conservative estimates of limit of detection (LOD) and limit of quantification (LOQ) for films optimizing XB interactions with f-MPC­(L2) were estimated in the range of ∼5–6 ppm and ∼18 ppm, respectively (>90% confidence).^73^ These values (LOD/LOQ) were significantly lower than measured for the control films: SWCNT with unf-MPCs (∼25 ppm/∼75 ppm) and SWCNTs alone (∼40 ppm/120 ppm). In comparison, other notable studies targeting CH detection via *hydrogen bonding* using SWCNTs modified with polymer wraps,^32^ urea-based molecules,^30^ or trifunctional silanes,^31^ reported either similar or higher LODs and similar response times compared to this study.

## 4 Conclusions

Experimental analysis of geometric and/or steric features contributing to XB bonding within solution and/or complex materials involving NPs or CNTs remains a relatively unexplored topic.^11–17^ This study converged traditional DFT measurements of XB interaction strength and geometries with experimental evidence from both solution and gas phase measurements that all suggest XB can be manipulated for more targeted interfacial interactions. While prior work has established the effectiveness of XB donor moieties such as perfluorinated halo-aromatics,^23^ this work harnessed that functionality at surface interfaces involving NMs that allowed for a fundamental study of XB interactions and the importance of XB geometry. Computational data supported multiple experimental observations from both solution electrochemistry and vapor-phase conductivity measurements. In all cases, XB interaction strength was found to scale with the strength of the donor entity, the XB acceptor strength, and most notably from our work, the manipulation of XB-donor capable SAMs and NP films that impacted the orientation of XB interactions. This work demonstrated further exploitation of versatile and functional gold NPs incorporated in conductivity-based sensing schemes featuring CNTs, and their use as a detection system for CH, a low volatile taggant found in nonaromatic explosives.

## Supplementary Material



## Notes

Additional experimental details regarding electrochemical measurements and DFT analysis; Spectroscopic, electrochemical, and microscopy characterization of unf- and f-MPCs (TEM, NMR, UV–vis); Schematics of vapor sensing instrumental set-ups; additional CV, *C*_dl_, and DFT results, including control experiments; SEM imaging of unmodified and modified IDA electrodes and additional *I–t* curves showing vapor responses (PDF)

## References

[1] Cavallo G., Metrangolo P., Milani R., Pilati T., Priimagi A., Resnati G., Terraneo G. (2016). The Halogen Bond. Chem. Rev..

[2] Politzer P., Murray J. S., Clark T. (2010). Halogen bonding:
an electrostatically-driven
highly directional noncovalent interaction. Phys. Chem. Chem. Phys..

[3] Clark T., Hennemann M., Murray J. S., Politzer P. (2007). Halogen bonding: the
sigma-hole. J. Mol. Model.

[4] Donald K. J., Wittmaack B. K., Crigger C. (2010). Tuning sigma-Holes: Charge Redistribution
in the Heavy (Group 14) Analogues of Simple and Mixed Halomethanes
Can Impose Strong Propensities for Halogen Bonding. J. Phys. Chem. A.

[5] Tawfik M., Donald K. J. (2014). Halogen Bonding:
Unifying Perspectives on Organic and
Inorganic Cases. J. Phys. Chem. A.

[6] Parker A. J., Stewart J., Donald K. J., Parish C. A. (2012). Halogen Bonding
in DNA Base Pairs. J. Am. Chem. Soc..

[7] Guo N., Maurice R., Teze D., Graton J., Champion J., Montavon G., Galland N. (2018). Experimental
and computational evidence
of halogen bonds involving astatine. Nat. Chem..

[8] Metrangolo P., Neukirch H., Pilati T., Resnati G. (2005). Halogen bonding based
recognition processes: A world parallel to hydrogen bonding. Acc. Chem. Res..

[9] Zapata F., Benitez-Benitez S. J., Sabater P., Caballero A., Molina P. (2017). Modulation of the Selectivity
in Anions Recognition
Processes by Combining Hydrogen- and Halogen-Bonding Interactions. Molecules.

[10] Ahangar A. A., Elancheran R., Dar A. A. (2022). Influence of halogen substitution
on crystal packing, molecular properties and electrochemical sensing. J. Solid State Chem..

[11] Pavan M. S., Durga Prasad K., Guru Row T. N. (2013). Halogen bonding
in fluorine: experimental
charge density study on intermolecular F···F and F···S
donor–acceptor contacts. Chem. Commun..

[12] Wang R., Dols T. S., Lehmann C. W., Englert U. (2012). The halogen bond made
visible: experimental charge density of a very short intermolecular
Cl···Cl donor–acceptor contact. Chem. Commun..

[13] Stephens S. L., Walker N. R., Legon A. C. (2011). Rotational
spectra and properties
of complexes B···ICF3 (B = Kr or CO) and a comparison
of the efficacy of ICl and ICF3 as iodine donors in halogen bond formation. J. Chem. Phys..

[14] Legon A. C. (2010). The halogen
bond: an interim perspective. Phys. Chem. Chem.
Phys..

[15] Guo H., Puttreddy R., Salminen T., Lends A., Jaudzems K., Zeng H., Priimagi A. (2022). Halogen-bonded shape memory polymers. Nat. Commun..

[16] Borissov A., Marques I., Lim J. Y. C., Felix V., Smith M. D., Beer P. D. (2019). Anion Recognition in Water by Charge-Neutral Halogen
and Chalcogen Bonding Foldamer Receptors. J.
Am. Chem. Soc..

[17] Gale P. A., Caltagirone C. (2018). Fluorescent
and colorimetric sensors for anionic species. Coordin Chem. Rev..

[18] Hein R., Beer P. D. (2022). Halogen bonding and chalcogen bonding
mediated sensing. Chem. Sci..

[19] Berger G., Frangville P., Meyer F. (2020). Halogen bonding for molecular recognition:
new developments in materials and biological sciences. Chem. Commun..

[20] Senesac L., Thundat T. G. (2008). Nanosensors for
trace explosive detection. Mater. Today.

[21] Jaini A. K. A., Hughes L. B., Kitimet M. M., Ulep K. J., Leopold M. C., Parish C. A. (2019). Halogen Bonding Interactions for Aromatic and Nonaromatic
Explosive Detection. ACS Sens..

[22] Weis J. G., Ravnsbaek J. B., Mirica K. A., Swager T. M. (2016). Employing Halogen
Bonding Interactions in Chemiresistive Gas Sensors. ACS Sens..

[23] Lai H., Leung A., Magee M., Almirall J. R. (2010). Identification of
volatile chemical signatures from plastic explosives by SPME-GC/MS
and detection by ion mobility spectrometry. Anal Bioanal Chem..

[24] Grate J. W., Ewing R. G., Atkinson D. A. (2012). Vapor-generation
methods for explosives-detection
research. Trac-Trend Anal Chem..

[25] Liang M., Guo L.-H. (2009). Application of nanomaterials
in environmental analysis
and monitoring. J. Nanosci. Nanotech..

[26] Guo S. J., Dong S. J. (2009). Biomolecule-nanoparticle
hybrids for electrochemical
biosensors. Trac-Trend Anal Chem..

[27] Pandey P., Datta M., Malhotra B. D. (2008). Prospects
of nanomaterials in biosensors. Anal. Lett..

[28] Fennell J. F., Liu S. F., Azzarelli J. M., Weis J. G., Rochat S., Mirica K. A., Ravnsbaek J. B., Swager T. M. (2016). Nanowire Chemical/Biological
Sensors: Status and a Roadmap for the Future. Angew. Chem. Int. Edit.

[29] Schroeder V., Savagatrup S., He M., Ling S. B., Swager T. M. (2019). Carbon
Nanotube Chemical Sensors. Chem. Rev..

[30] Schnorr J. M., van der Zwaag D., Walish J. J., Weizmann Y., Swager T. M. (2013). Sensory
Arrays of Covalently Functionalized Single-Walled Carbon Nanotubes
for Explosive Detection. Adv. Funct Mater..

[31] Frazier K. M., Swager T. M. (2013). Robust Cyclohexanone Selective Chemiresistors Based
on Single-Walled Carbon Nanotubes. Anal. Chem..

[32] Yoon B., Choi S. J., Swager T. M., Walsh G. F. (2021). Flexible Chemiresistive
Cyclohexanone Sensors Based on Single-Walled Carbon Nanotube-Polymer
Composites. ACS Sens..

[33] Fennell J. E., Hamaguchi H., Yoon B., Swager T. M. (2017). Chemiresistor Devices
for Chemical Warfare Agent Detection Based on Polymer Wrapped Single-Walled
Carbon Nanotubes. Sensors.

[34] Taylor A.
J., Wilmore J. T., Beer P. D. (2024). Halogen bonding BODIPY-appended pillar[5]­arene
for the optical sensing of dicarboxylates and a chemical warfare agent
simulant. Chem. Commun..

[35] Aakeröy C. B., Beer P. D., Beyeh N. K., Brammer L., Branca M., Bryce D. L., Del Bene J. E., Edwards A. J., Erdelyi M., Esterhuysen C., Fourmigue M., Kennepohl P., Lee L. M., Mosquera M. E. G., Murray J. S., Mustoe C. L., Pennington W. T., Politzer P., Riley K. E., Rosokha S. V., Scheiner S., Taylor M. S., Tsuzuki S., Vargas-Baca I., Xu Y. (2017). The halogen bond in solution: general discussion. Faraday Discuss..

[36] Dang Q. M., Gilmore S. T., Lalwani K., Conk R. J., Simpson J. H., Leopold M. C. (2022). Monolayer-Protected Gold Nanoparticles Functionalized
with Halogen Bonding Capability—An Avenue for Molecular Detection
Schemes. Langmuir.

[37] Donald K. J., Tawfik M. (2013). The Weak Helps the
Strong: Sigma-Holes and the Stability
of MF4 center dot Base Complexes. J. Phys. Chem.
A.

[38] Dang Q. M., Simpson J. H., Parish C. A., Leopold M. C. (2021). Evaluating Halogen-Bond
Strength as a Function of Molecular Structure Using Nuclear Magnetic
Resonance Spectroscopy and Computational Analysis. J. Phys. Chem. A.

[39] Becke A. D. (1993). Density-functional
thermochemistry. III. The role of exact exchange. J. Chem. Phys..

[40] Lee C. T., Yang W. T., Parr R. G. (1988). Development
of the Colle-Salvetti
Correlation-Energy Formula into a Functional of the Electron-Density. Phys. Rev. B.

[41] Stephens P. J., Devlin F. J., Chabalowski C. F., Frisch M. J. (1994). Ab-Initio Calculation
of Vibrational Absorption and Circular-Dichroism Spectra Using Density-Functional
Force-Fields. J. Phys. Chem. A.

[42] Zhao Y., Truhlar D. G. (2008). The M06 suite of density functionals for main group
thermochemistry, thermochemical kinetics, noncovalent interactions,
excited states, and transition elements: two new functionals and systematic
testing of four M06-class functionals and 12 other functionals. Theor. Chem. Acc..

[43] Chai J. D., Head-Gordon M. (2008). Long-range
corrected hybrid density functionals with
damped atom-atom dispersion corrections. Phys.
Chem. Chem. Phys..

[44] Weigend F., Ahlrichs R. (2005). Balanced basis sets
of split valence, triple zeta valence
and quadruple zeta valence quality for H to Rn: Design and assessment
of accuracy. Phys. Chem. Chem. Phys..

[45] Dunning T.
H. (1989). Gaussian-Basis
Sets for Use in Correlated Molecular Calculations 1. The Atoms Boron
through Neon and Hydrogen. J. Chem. Phys..

[46] Peterson K.
A., Shepler B. C., Figgen D., Stoll H. (2006). On the spectroscopic
and thermochemical properties of ClO, BrO, IO, and their anions. J. Phys. Chem. A.

[47] Stoll H., Metz B., Dolg M. (2002). Relativistic
energy-consistent pseudopotentials--recent
developments. J. Comput. Chem..

[48] Energy-consistent Pseudopotentials of the Stuttgart/Cologne Group 2025 https://www.tc.uni-koeln.de/PP/clickpse.en.html. (accessed July, 17, 2025).

[49] Marenich A. V., Cramer C. J., Truhlar D. G. (2009). Universal Solvation Model Based on
Solute Electron Density and on a Continuum Model of the Solvent Defined
by the Bulk Dielectric Constant and Atomic Surface Tensions. J. Phys. Chem. B.

[50] Javaly N., McCormick T. M., Stuart D. R. (2024). A comparison of structure, bonding
and non-covalent interactions of aryl halide and diarylhalonium halogen-bond
donors. Beilstein J. Org. Chem..

[51] Murray J. S., Riley K. E., Politzer P., Clark T. (2010). Directional Weak Intermolecular
Interactions: σ-Hole Bonding. Aust. J.
Chem..

[52] Frisch, M. J. ; Trucks, G. W. ; Schlegel, H. B. ; Scuseria, G. E. ; Robb, M. A. ; Cheeseman, J. R. ; Scalmani, G. ; Barone, V. ; Petersson, G. A. ; Nakatsuji, H. Gaussian 16; Gaussian Inc: Wallington, CT, 2016.

[53] Shao Y. H., Gan Z. T., Epifanovsky E., Gilbert A. T. B., Wormit M., Kussmann J., Lange A. W., Behn A., Deng J., Feng X. T., Ghosh D., Goldey M., Horn P. R., Jacobson L. D., Kaliman I., Khaliullin R. Z., Kus T., Landau A., Liu J., Proynov E. I., Rhee Y. M., Richard R. M., Rohrdanz M. A., Steele R. P., Sundstrom E. J., Woodcock H., Zimmerman P. M., Zuev D., Albrecht B., Alguire E., Austin B., Beran G. J. O., Bernard Y. A., Berquist E., Brandhorst K., Bravaya K. B., Brown S. T., Casanova D., Chang C. M., Chen Y. Q., Chien S. H., Closser K. D., Crittenden D. L., Diedenhofen M., DiStasio R., Do H., Dutoi A. D., Edgar R. G., Fatehi S., Fusti-Molnar L., Ghysels A., Golubeva-Zadorozhnaya A., Gomes J., Hanson-Heine M. W. D., Harbach P. H. P., Hauser A. W., Hohenstein E. G., Holden Z. C., Jagau T. C., Ji H. J., Kaduk B., Khistyaev K., Kim J., Kim J., King R. A., Klunzinger P., Kosenkov D., Kowalczyk T., Krauter C. M., Lao K. U., Laurent A. D., Lawler K. V., Levchenko S. V., Lin C. Y., Liu F., Livshits E., Lochan R. C., Luenser A., Manohar P., Manzer S. F., Mao S. P., Mardirossian N., Marenich A. V., Maurer S. A., Mayhall N. J., Neuscamman E., Oana C. M., Olivares-Amaya R., O’Neill D. P., Parkhill J. A., Perrine T. M., Peverati R., Prociuk A., Rehn D. R., Rosta E., Russ N. J., Sharada S. M., Sharma S., Small D. W., Sodt A., Stein T., Stück D., Su Y. C., Thom A. J. W., Tsuchimochi T., Vanovschi V., Vogt L., Vydrov O., Wang T., Watson M. A., Wenzel J., White A., Williams C. F., Yang J., Yeganeh S., Yost S. R., You Z. Q., Zhang I. Y., Zhang X., Zhao Y., Brooks B. R., Chan G. K. L., Chipman D. M., Cramer C. J., Goddard W., Gordon M. S., Hehre W. J., Klamt A., Schaefer H., Schmidt M. W., Sherrill C. D., Truhlar D. G., Warshel A., Xu X., Aspuru-Guzik A., Baer R., Bell A. T., Besley N. A., Chai J. D., Dreuw A., Dunietz B. D., Furlani T. R., Gwaltney S. R., Hsu C. P., Jung Y. S., Kong J., Lambrecht D. S., Liang W. Z., Ochsenfeld C., Rassolov V. A., Slipchenko L. V., Subotnik J. E., Van Voorhis T., Herbert J. M., Krylov A. I., Gill P. M. W., Head-Gordon M. (2015). Advances in molecular quantum chemistry
contained in the Q-Chem 4 program package. Mol.
Phys..

[54] Loftus A. F., Reighard K. P., Kapourales S. A., Leopold M. C. (2008). Monolayer-Protected
Nanoparticle Film Assemblies as Platforms for Controlling Interfacial
and Adsorption Properties in Protein Monolayer Electrochemistry. J. Am. Chem. Soc..

[55] Leopold M. C., Doan T. T., Mullaney M. J., Loftus A. F., Kidd C. M. (2015). Electrochemical
characterization of self-assembled monolayers on gold substrates derived
from thermal decomposition of monolayer-protected cluster films. J. Appl. Electrochem..

[56] Sherard M. M., Kaplan J. S., Simpson J. H., Kittredge K. W., Leopold M. C. (2024). Functionalized Gold Nanoparticles
and Halogen Bonding
Interactions Involving Fentanyl and Fentanyl Derivatives. Nanomaterials.

[57] Brust M., Walker M., Bethell D., Schriffrin D. J., Whyman R. J. (1994). Synthesis of thiol-derivatised gold
nanoparticles in
a two-phase Liquid–Liquid system. J.
Chem. Soc., Chem. Commun..

[58] Hostetler M. J., Wingate J. E., Zhong C. J., Harris J. E., Vachet R. W., Clark M. R., Londono J. D., Green S. J., Stokes J. J., Wignall G. D., Glish G. L., Porter M. D., Evans N. D., Murray R. W. (1998). Alkanethiolate Gold
Cluster Molecules with Core Diameters
from 1.5 to 5.2 nm: Core and Monolayer Properties as a Function of
Core Size. Langmuir.

[59] Hicks J. F., Templeton A. C., Chen S. W., Sheran K. M., Jasti R., Murray R. W., Debord J., Schaaf T. G., Whetten R. L. (1999). The monolayer
thickness dependence of quantized double-layer capacitances of monolayer-protected
gold clusters. Anal. Chem..

[60] Templeton A. C., Hosteler M. J., Murray R. W. (1999). DYnamics of Place Exchange Reactions
on Monolayer-Protected Gold Cluster Molecules. Langmuir.

[61] Mirica K. A., Weis J. G., Schnorr J. M., Esser B., Swager T. M. (2012). Mechanical
Drawing of Gas Sensors on Paper. Angew. Chem.
Int. Ed.

[62] Nakano K., Yoshitake T., Yamashita Y., Bowden E. F. (2007). Cytochrome self-assembly
on alkanethiol monolayer electrodes as characterized by AFM, IR, QCM,
and direct electrochemistry. Langmuir.

[63] Petrović J., Clark R. A., Yue H., Waldeck D. H., Bowden E. F. (2005). Impact
of Surface Immobilization and Solution Ionic Strength on the Formal
Potential of Immobilized Cytochrome c. Langmuir.

[64] Finklea, H. O. Electrochemistry of Organized Monolayers of Thiols and Related Molecules on Electrodes. In Electroanal. Chem.: A Series of Advances; Bard, A. J. a. ; R, I. , Eds.; Marcel Dekker, Inc: New York, 1996; Vol. 19, pp 109–335.

[65] Ciancaleoni G., Bertani R., Rocchigiani L., Sgarbossa P., Zuccaccia C., Macchioni A. (2015). Discriminating Halogen-Bonding from
Other Noncovalent Interactions by a Combined NOE NMR/DFT Approach. Chem.- Eur. J..

[66] Sarwar M. G., Dragisic B., Salsberg L. J., Gouliaras C., Taylor M. S. (2010). Thermodynamics of Halogen Bonding in Solution: Substituent,
Structural, and Solvent Effects. J. Am. Chem.
Soc..

[67] Lu H., Zeysing D., Kind M., Terfort A., Zharnikov M. (2013). Structure
of Self-Assembled Monolayers of Partially Fluorinated Alkanethiols
with a Fluorocarbon Part of Variable Length on Gold Substrate. J. Phys. Chem. C.

[68] Zenasni O., Jamison A. C., Lee T. R. (2013). The impact
of fluorination on the
structure and properties of self-assembled monolayer films. Soft Matter.

[69] Wang H., Shen Q. J., Wang W. (2017). Weakening
and Leveling Effect of
Solvent Polarity on Halogen Bond Strength of Diiodoperfluoroalkane
with Halide. J. Solution Chem..

[70] Robertson C. C., Wright J. S., Carrington E. J., Perutz R. N., Hunter C. A., Brammer L. (2017). Hydrogen bonding vs. halogen bonding: the solvent decides. Chem. Sci..

[71] Sardar R., Funston A. M., Mulvaney P., Murray R. W. (2009). Gold Nanoparticles:
Past, Present, and Future. Langmuir.

[72] Erdélyi M. (2012). Halogen bonding
in solution. Chem. Soc. Rev..

[73] Skoog, D. A. W. ; D, M. ; Holler, F. J. ; Crouch, S. R. Fundamentals of Analytical Chemistry, 8th ed.; Thomson Brooks/Cole: Belmont, CA, 2004.

