# Supplementary material for: Design of Nanomaterial-Based Sensors for Enhanced Halogen Bonding
Source: ACS Omega. 2026 Jan 14;11(3):4006–20. doi: 10.1021/acsomega.5c07542 (PMC12854608; doi:10.1021/acsomega.5c07542)
Supplement: Supplementary file 1 [file ao5c07542_si_001.pdf]

Supporting Information: **Design of Nanomaterial-based Sensors for Enhanced Halogen Bonding**

Ben H. Edelman, Charles W. Sheppard, Lucas A. Chuidian, Arielle Vinnikov, Carol A. Parish, Felix Bevc[†], Kevin W. Kittredge[†], Lillian B. Hughes, and **Michael C. Leopold***

**Department of Chemistry, Gottwald Center for the Sciences, University of Richmond, Richmond, Virginia 23173, United States (Corresponding Author)*

[A] Department of Chemistry and Biochemistry, Virginia Wesleyan University, Virginia Beach, VA 23455, United States

Table of Contents:

▪ Electrochemical "sandwich cell" or Bowden-Cell schematic: **Fig. SI-1**

▪ TEM imaging/analysis of as prepared hexanethiolate (C6) monolayer-protected clusters (MPCs): **Fig. SI-2**

▪ UV-Vis analysis of unfunctionalized and functionalized MPCs: **Fig. SI-3**

▪ ^{1}H-NMR spectra of unf-MPCs before/after iodine decomposition: **Fig. SI-4, Fig. SI-5**

▪ TEM imaging/analysis of removed fraction of C6-MPC sample: **Fig. SI-6**

▪ TEM imaging/analysis of a soluble fraction of C6-MPC: **Fig. SI-7**

▪ Histogram analysis from TEM imaging of as prepared vs. fractionated C6-MPCs: **Fig. SI-8**

▪ ^{1}H-NMR and ^{19}F-NMR spectra of Br-L2 functionalized MPCs (f-MPCs) before/after I_2 decomposition: **Fig. SI-9**

▪ ^{19}F-NMR spectra of Iodine-L2 functionalized MPCs (f-MPCs) before/after I_2 decomposition: **SI-10**

▪ Rudimentary gas generating system used to deliver varying amounts of chemical vapor to the sensing interface: Fig. **SI-11**

▪ Schematic of permeation tube instrumentation that delivers specific concentrations of chemical vapor across the sensing interface: **Fig. SI-12**

▪ Capacitance (C_{dl}) measurements of unmodified and SAM-modified (C6, L1 or L2) gold electrodes: **Fig. SI-13**

▪ C_{dl} and FeCN cyclic voltammetry (CV) measurements of bare vs. SAM-modified (L1 and C8) gold electrodes: **Fig. SI-14**

▪ CV overlay showing C_{dl} of bare gold electrode versus SAM-modified (C6, L1, and L2) gold electrodes: **Fig. SI-15**

▪ Additional DFT analysis details and geometry optimized structures for L1 and L2 in the gas and aqueous phases: **Fig. SI-16**

▪ C_{dl} measurements of uniform C6-SAMs before and after exposure to L1 or L2 to create mixed SAMs: **Fig. SI-17**

▪ Capacitance overlay of a bare gold electrode with SAM-modified gold electrodes containing uniform L2 or C6/L2 mixed SAM: **Fig. SI-18**

-continued on next page...

Table of Contents continued:

- Example of linear sweep voltammetry overlay of L1 SAM and C6/L1 mixed SAM: **Fig. SI-19**
- Capacitance (C_{dl}) measurements of a C6-SAM and a C6/L1 mixed SAM before and 12 hours after exposure to DABCO followed by extensive washing with polar solvents: **Fig. SI-20**
- Tracking of capacitance measurements of a C6/L1 mixed SAM before and varying amounts of time (2-3 hours, 12 hours) after exposure to DABCO: **Fig. SI-21**
- C_{dl} voltammetry scan overlay of MUA/C6 mixed SAM before and 12 hours after exposure to DABCO: **Fig. SI-22**
- Representative C_{dl} scans of gold electrodes modified with a uniform L1 SAM, uniform L2 SAM, mixed C6/L1 SAM, and mixed C6/L2 SAM before and after exposure to 1-BP: **Fig. SI-23**
- C_{dl} scan overlay of gold electrodes modified with C6/C10 mixed-SAM before and after exposure to 1-BP: **Fig. SI-24**
- **Table SI-0:** Numerical results for C_{dl} measurements of various systems.
- C_{dl} voltammetry of unmodified and C6-SAM modified gold electrodes prior to MPC film assembly: **Fig. SI-25**
- C_{dl} scan overlay of C6-SAM modified gold electrodes after first and second exposure to UDDT-linked L2-f-MPCs: **Fig. SI-26**
- C_{dl} and CV measurements of C6-SAM modified gold electrodes before and after two exposures to NDT-linked unf-MPCs: **Fig. SI-27**
- C_{dl} scan overlay of gold electrodes modified with a C6-SAM and f-MPCs (L1) before and after exposure to DABCO: **Fig. SI-28**
- C_{dl} scan overlay of gold electrodes modified with a C6-SAM and f-MPCs (L1) before and after exposure to 1-BP: **Fig. SI-29**
- C_{dl} scans of gold electrodes modified with C6-SAM and unf-MPCs before and after exposure to 1-BP: **Fig. SI-30**
- **Table SI-1:** Comparison of results from different levels of DFT theory
- **Table SI-2:** B3LYP/cc-pVDZ (gas)
- **Table SI-3:** M06-2x/cc-pVTZ// M06-2x/cc-pVDZ (gas)
- **Table SI-4:** ωB97X-D/cc-pVTZ//ωB97X-D/cc-pVDZ (gas)
- **Table SI-5:** M06-2x/cc-pVDZ (solvent = water)
- **Table SI-6:** ωB97X-D/def2-STVDP (gas)

- continued on next page…

Table of Contents continued:

▪ Geometry optimizations of the 1-BP - L2 interaction: **Fig. SI-31**

▪ Geometry optimizations of the Cyclohexanone - L2 interaction : **Fig. SI-32**

▪ Geometry optimizations of the DABCO - L2 interaction : **Fig. SI-33**

▪ Geometry optimizations of the DMNB - L2 interaction : **Fig. SI-34**

▪ Geometry optimizations of the RDX – L2 interaction : **Fig. SI-35**

▪ Geometry optimizations of the TNT site1 - L2 interaction : **Fig. SI-36**

▪ Geometry optimizations of the TNT site2 - L2 interaction : **Fig. SI-37**

▪ Electrostatic potentials (ESPs) : **Fig. SI-38**

▪ SEM imaging examples of clean and modified (SWCNT with f-MPC(L2)) IDAs: **Fig. SI-39**

▪ Cyclohexanone sensitivity optimization of mass ratio of f-MPC:SWCNT for PENCIL: **Fig. SI-40**

▪ Sensing response (normalized conductance) of films in Figure 8B: **Fig. SI-41**

▪ Examples of sensing response in terms current and normalized conductance [$\Delta G/G_o\%$] for films of the following:

- SWCNT+f-MPC(L2-I): **Fig. SI-42**
- SWCNT+f-MPC(L2-Br): **Fig. SI-43**
- SWCNT+unf-MPC: **Fig. SI-44**
- SWCNT (control): **Fig. SI-45**

▪ I-t curves collected as three successive pulses of different concentrations of CH vapor flow over an IDA modified with SWCNTs with f-MPC (L2-I): **Fig. SI-46**

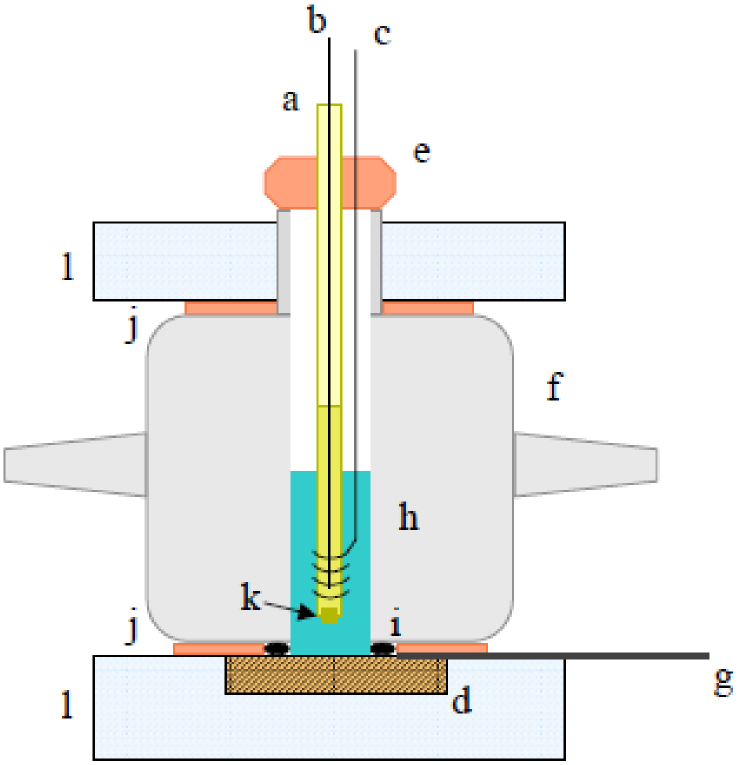

Figure SI-1. Schematic illustration of electrochemical "sandwich" cell (i.e., Bowden electrochemical cell) used in this study and featuring reference electrode comprised of (a) glass barrel with 1 M KCl; (b) Ag wire with AgCl (s); (c) platinum wire counter electrode; (d) gold working electrode; (e) rubber stopper; (f) glass cell body with temperature control jacket; (g) polished brass electrical connection to working electrode; (h) electrolyte solution; (i) o-ring; (j) rubber gaskets; (k) glass frit salt bridge; (l) Lucite retainer plates with threaded rods and wing-nut pressurization.

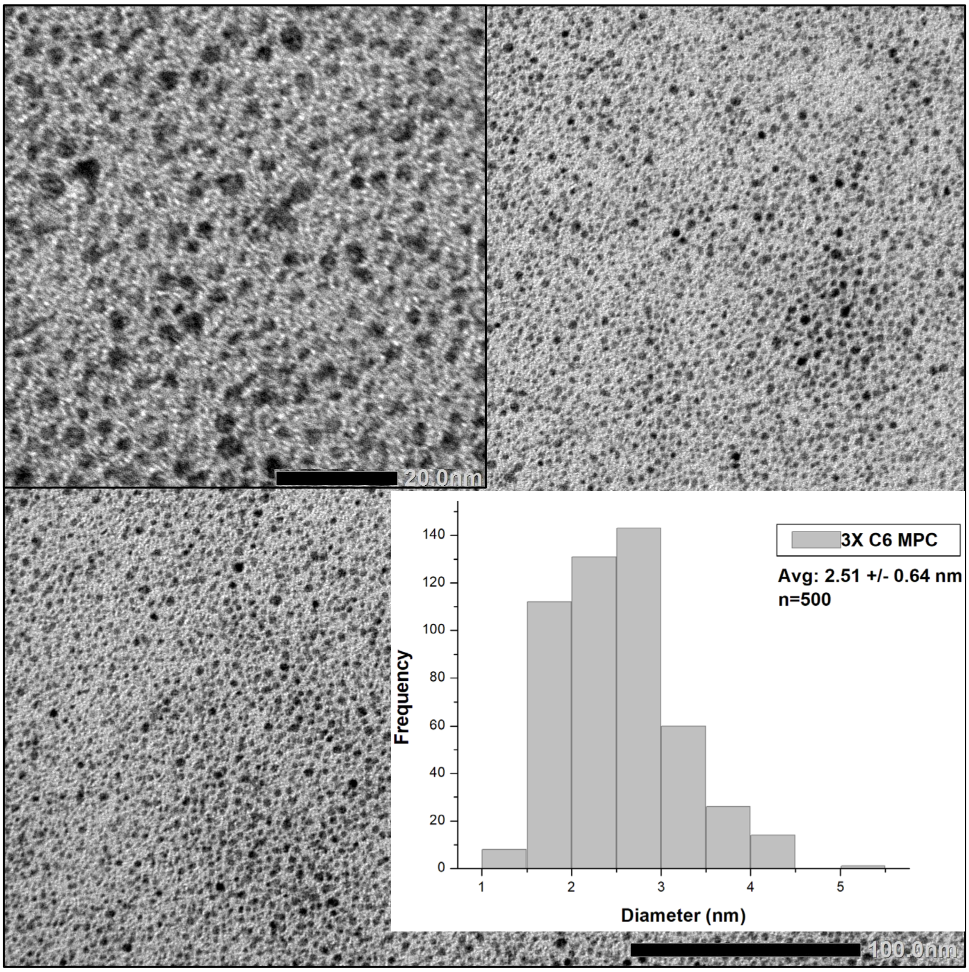

Figure SI-2. TEM imaging and analysis of hexanethiolate-protected C6 MPC including, 80kV scan with insets showing enlarged 200 kV scan (*top, left*) and histogram analysis results for average diameter determination (*bottom right*) where n = 500 particles analyzed.

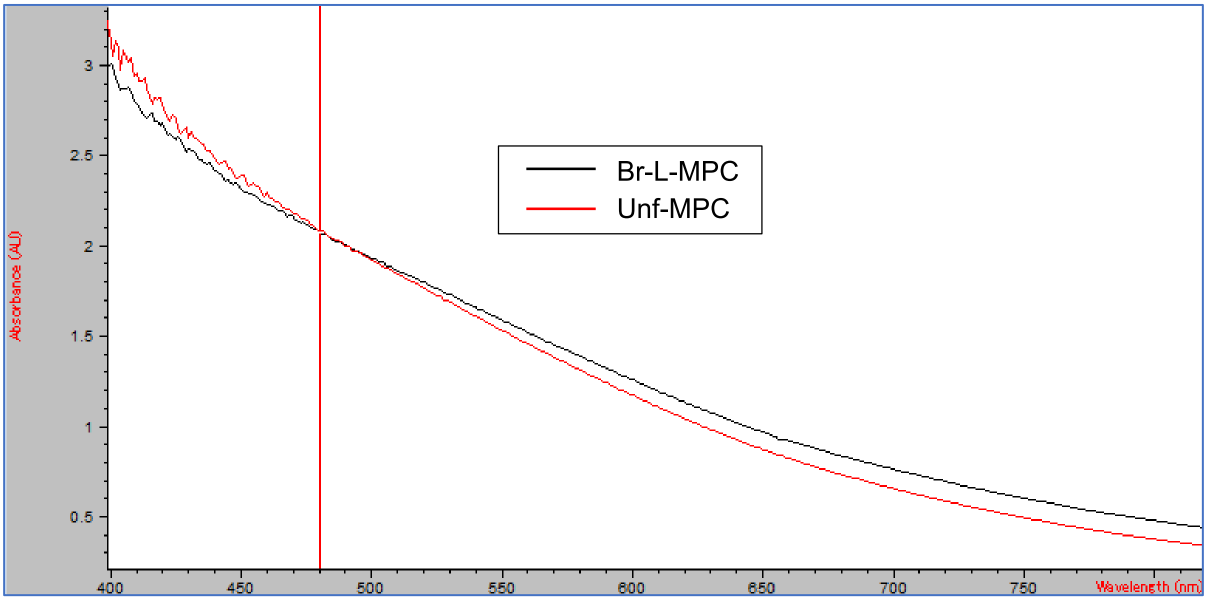

Figure SI-3. Typical representations of UV-Vis spectra of unf-MPCs (C6 MPCs, red) and f-MPCs (e.g., Br-L2 black) in toluene.

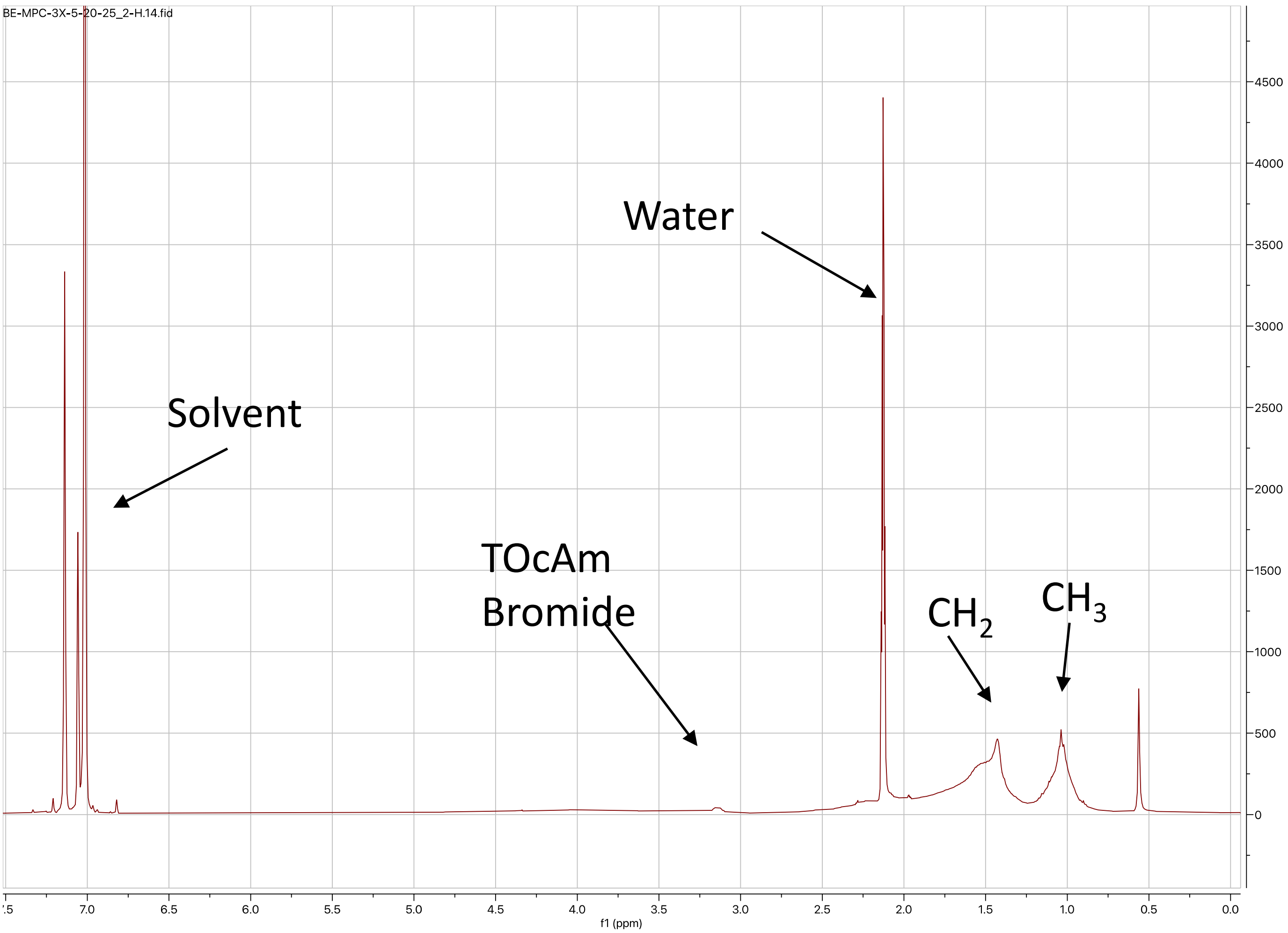

Figure SI-4. ^{1}H NMR depicting C6 MPC (unf) before iodine treatment.

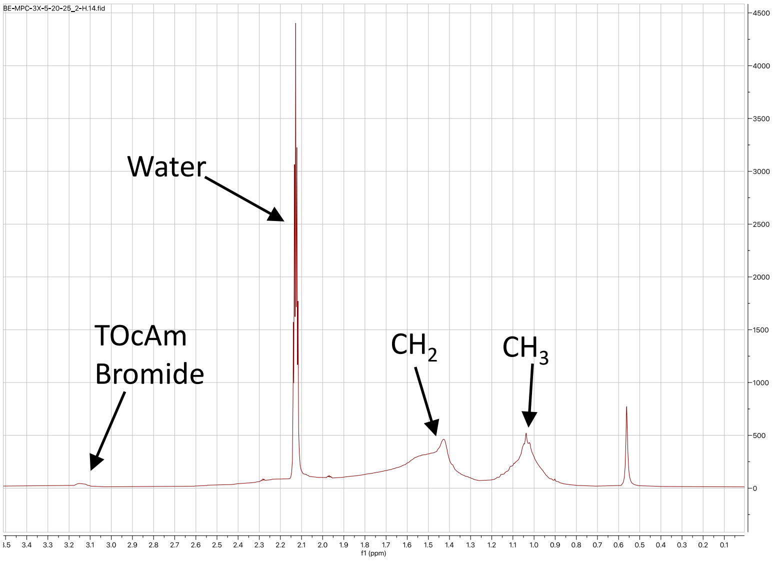

Zoomed in (1.5-0 ppm)

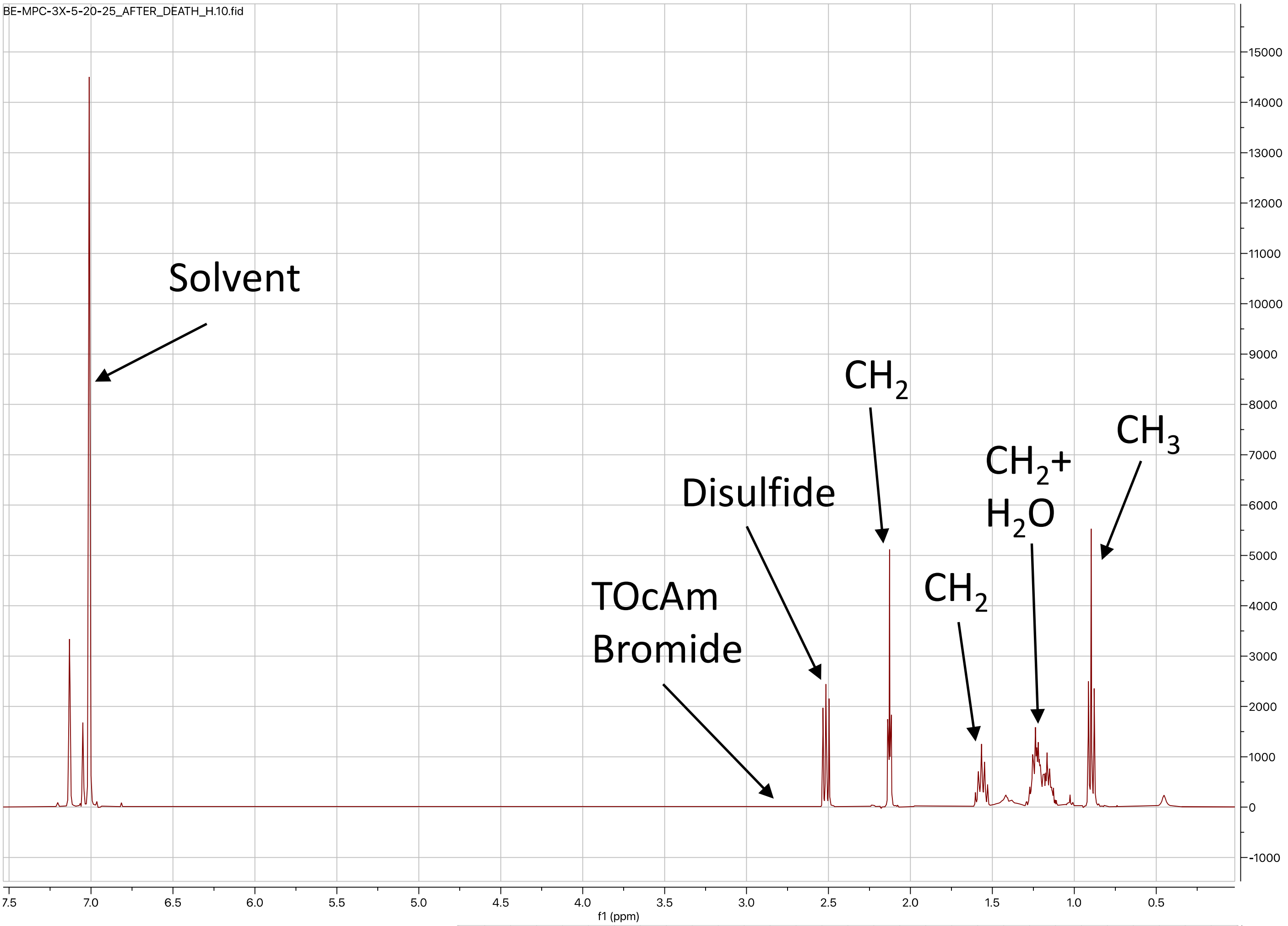

Zoomed in (3.0 -0.0ppm)

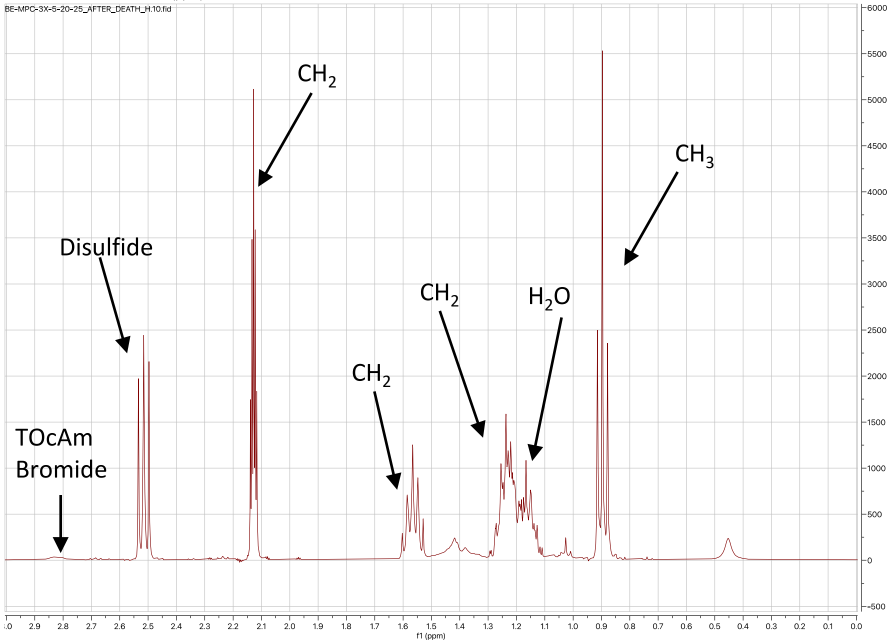

Figure SI-5. ^{1}H NMR depicting C6 MPC (unf) after iodine treatment (decomposition).

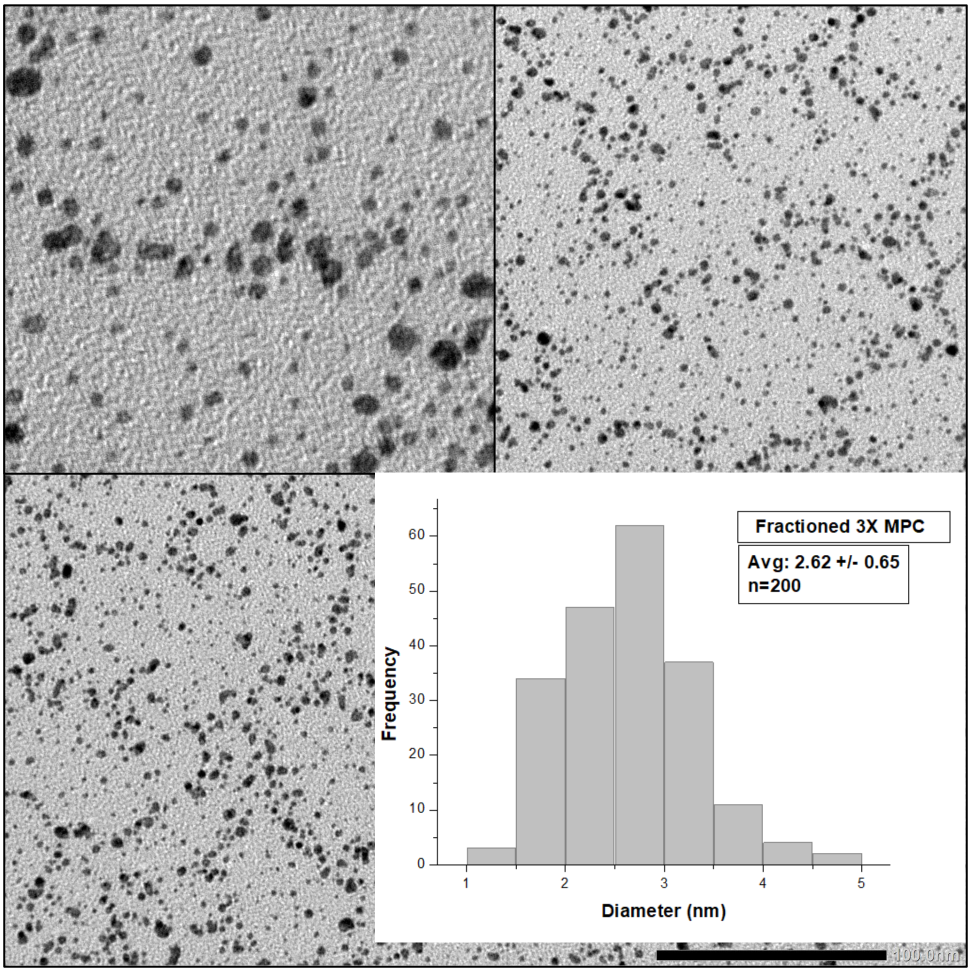

Figure SI-6. TEM TEM imaging and analysis of Fraction-A (precipitated via acetone/toluene mixtures) of C6 MPC including, 80kV scan with insets showing enlarged 200kV scan (*top, left*) and histogram analysis results for average diameter determination (*bottom, right*) where n=200 particles analyzed.

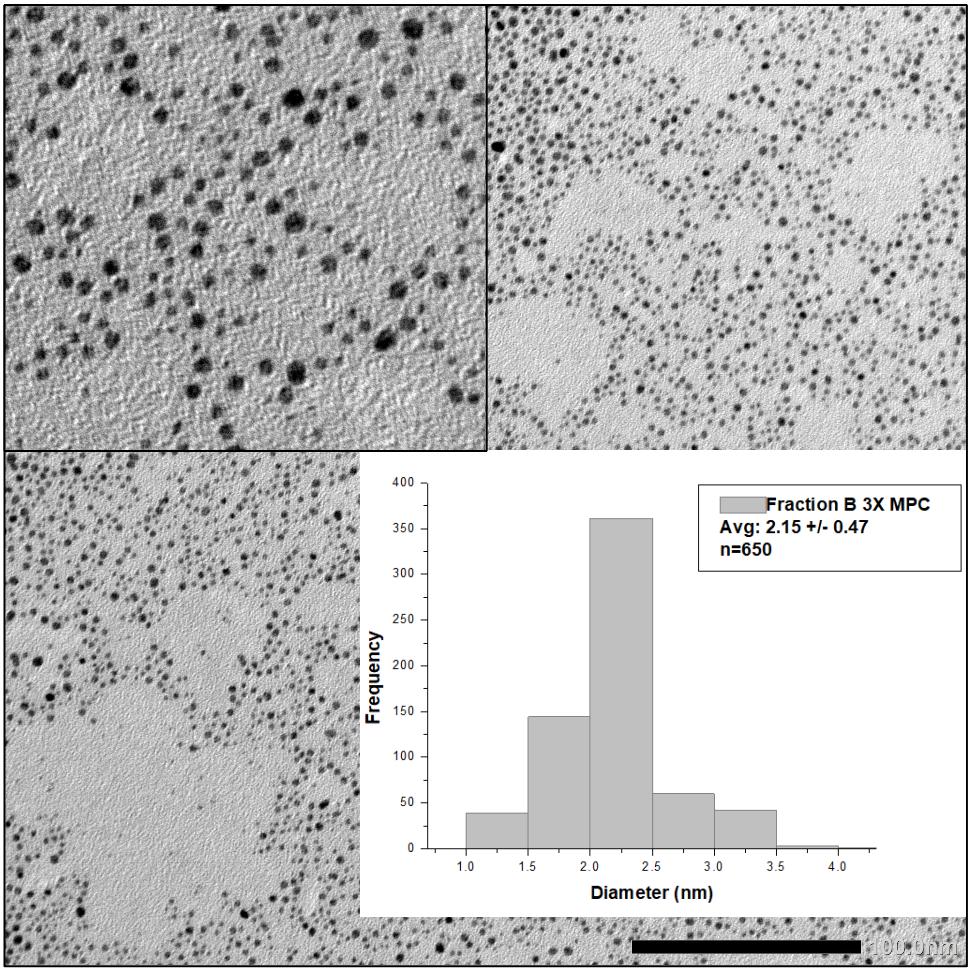

Figure SI-7. TEM imaging and analysis of soluble fraction of hexanethiolate-protected C6 MPC sample (after removing Fractions A and B) including, 80kV scan with insets showing enlarged 200kV scan (*top, left*) and histogram analysis results for average diameter determination (*bottom, right*) where n=650 particles analyzed.

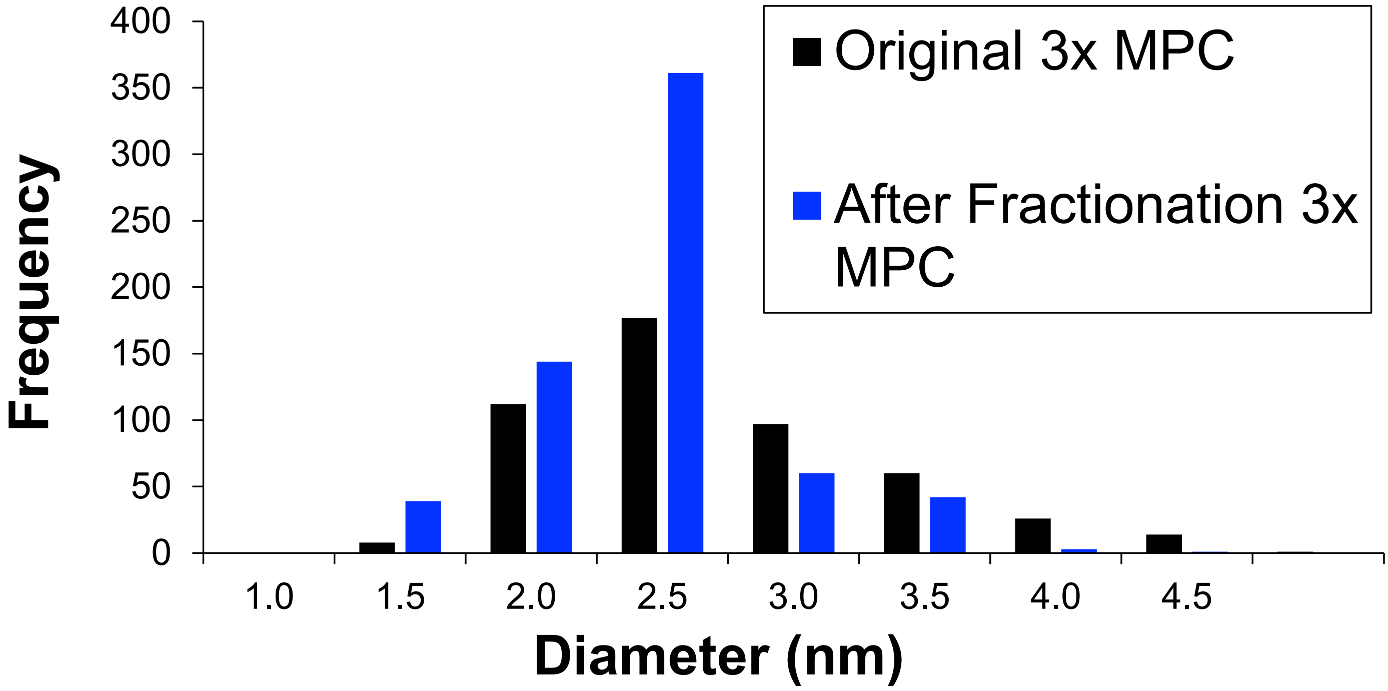

Figure SI-8. Comparison of histogram analysis from TEM imaging of as prepared C6-MPCs versus fractionated C6-MPCs.

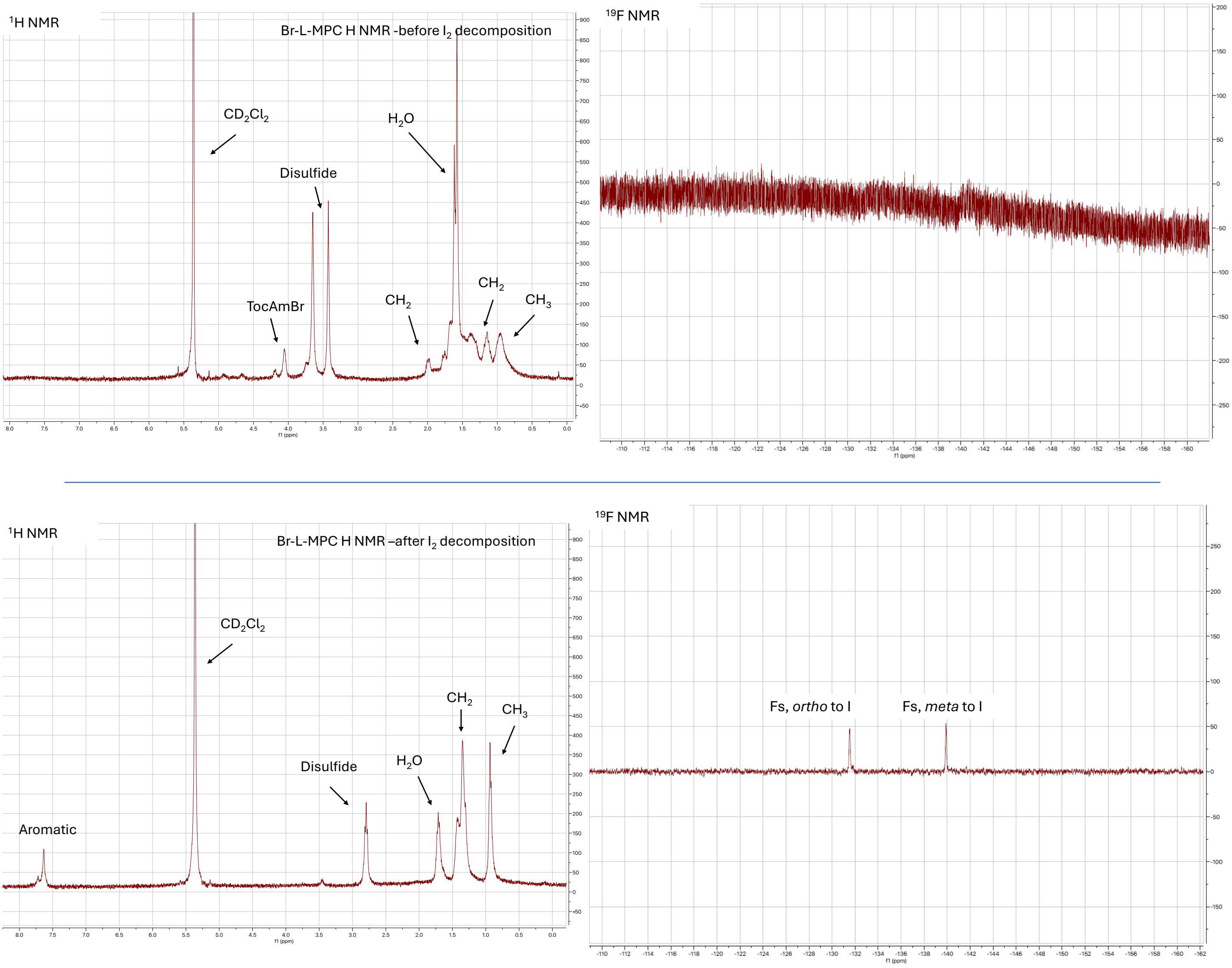

Figure SI-9. ^{1}H-NMR (*left*) and ^{19}F-NMR (*right*) of f-MPC (Br-L2) before (*top*) and after (*bottom*) decomposition with iodine.

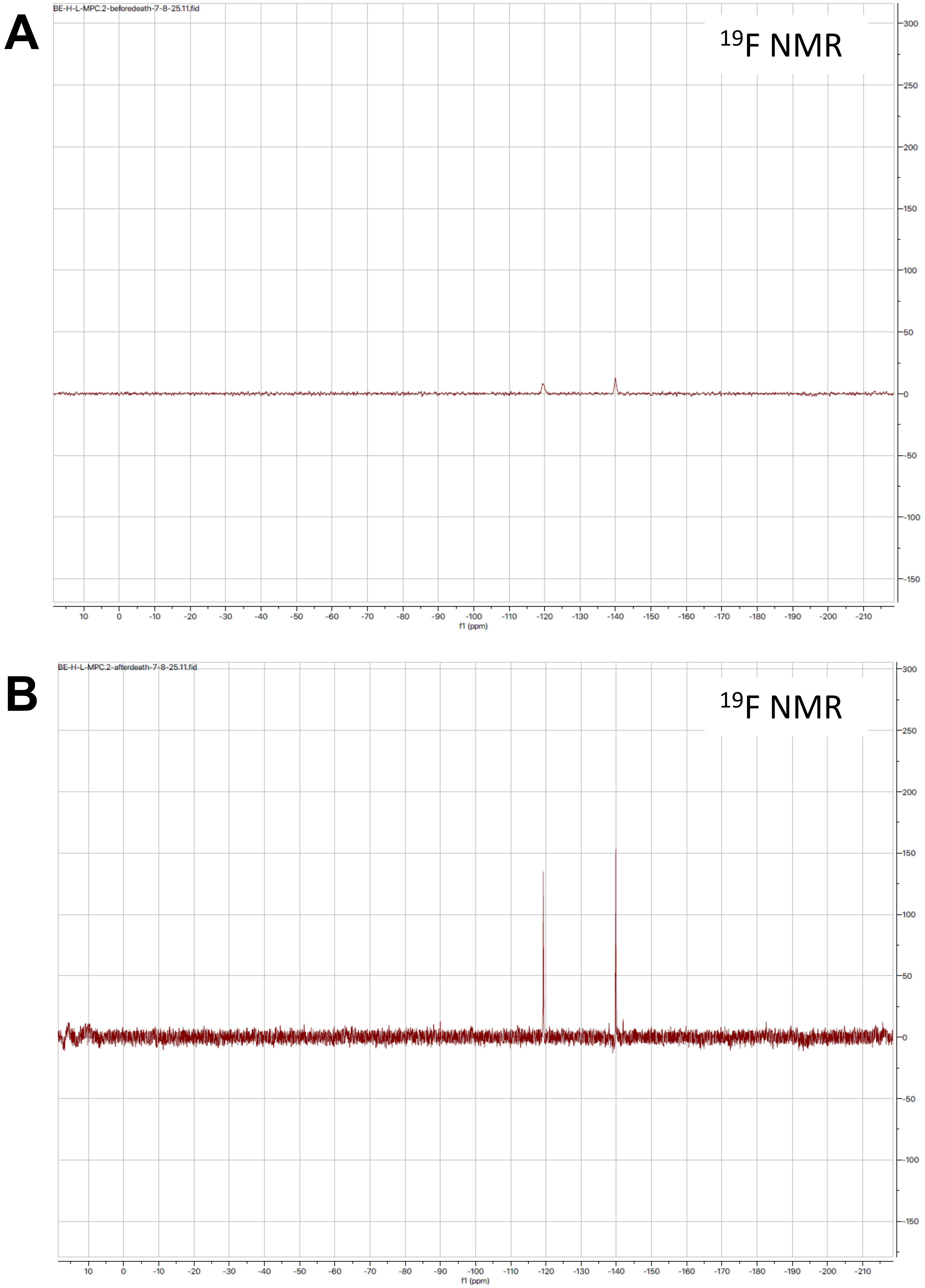

Figure SI-10. ^{19}F-NMR of f-MPC (Iodine-L2) before (A) and after (B) decomposition with iodine.

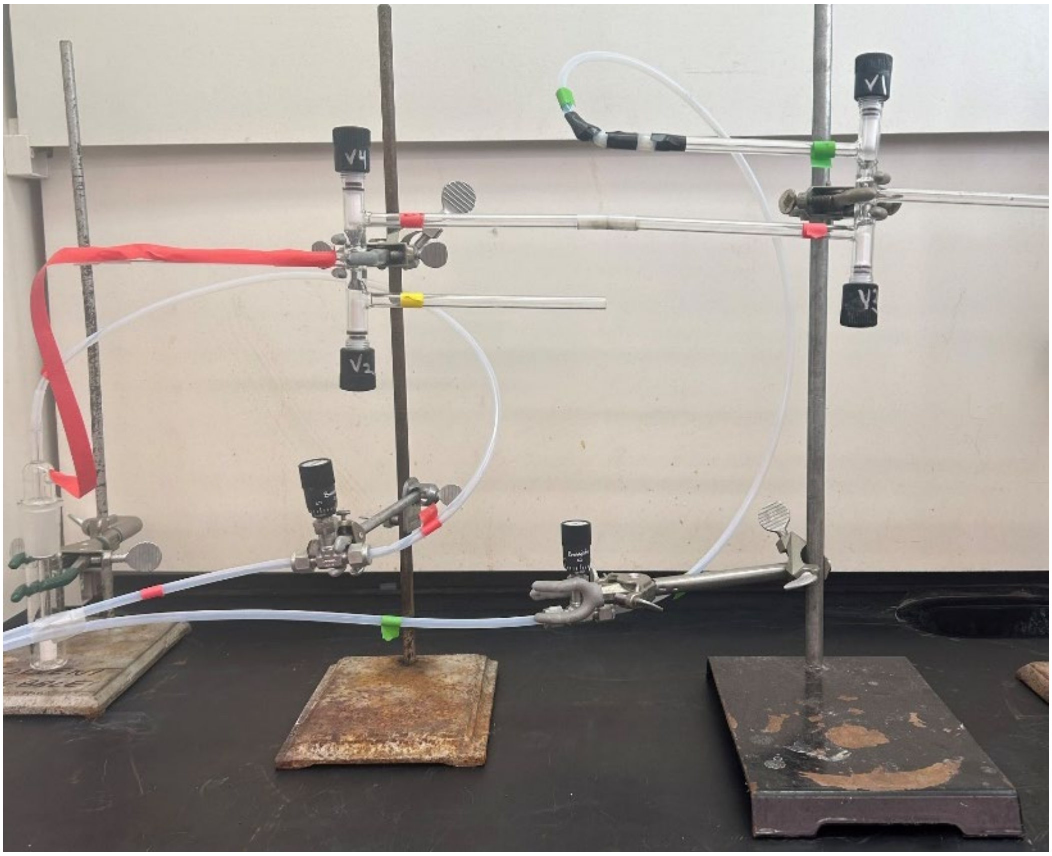

Figure SI-11. Rudimentary chemical vapor system used to mix N_2 with chemical vapor generated from a solvent filled bubbler and ratioed with Swage-valves. System was used to generate chemical vapor estimated in the 100s of ppm.

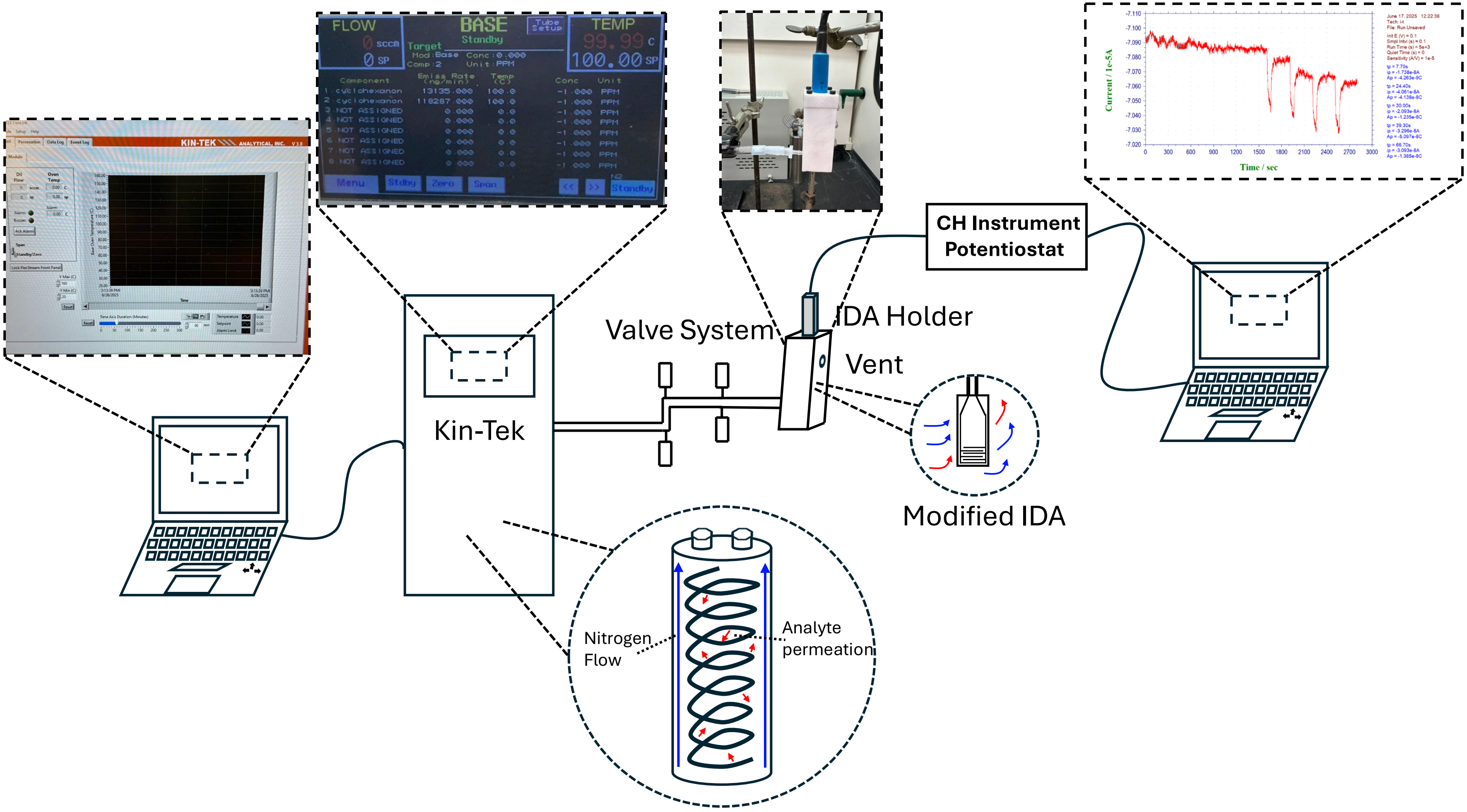

Figure SI-12. Schematic of chemical vapor instrumentation including (*from left to right*) PC with KinTek FlexLink (v3.0), Kin-Tek Permation Tube Gas Generating System, permeation tube with cyclohexanone, valve system, Teflon cell chamber with IDA holder, and CH Instrument potentiostat with PC control for amperometric measurements (I-t curves) during calibrated ppm delivery of gas vapor.

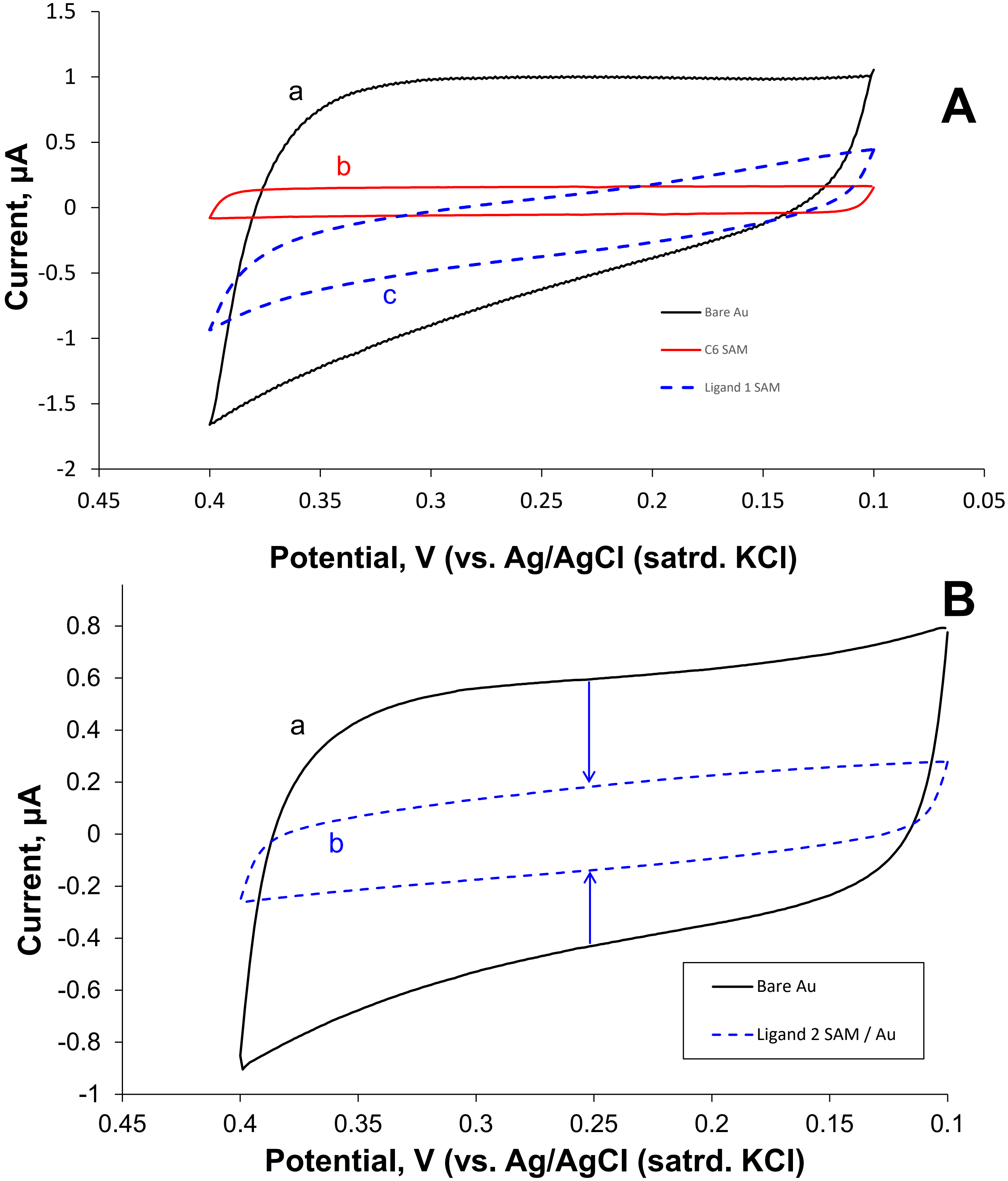

Figure SI-13. **(A)** Capacitance measurements using CV (100 mV/sec; 4.4. mM PBS) of gold electrodes **(a)** cleaned (unmodified) and SAM-modified with either **(b)** hexanethiol (C6) or **(c)** ligand 1 (L1) where the decrease in capacitance is consistent with thiol behavior; **(B)** analogous measurements at bare gold and L2 SAMs showing the same thiol behavior (i.e., both L1 and L2 readily form SAMs at gold).

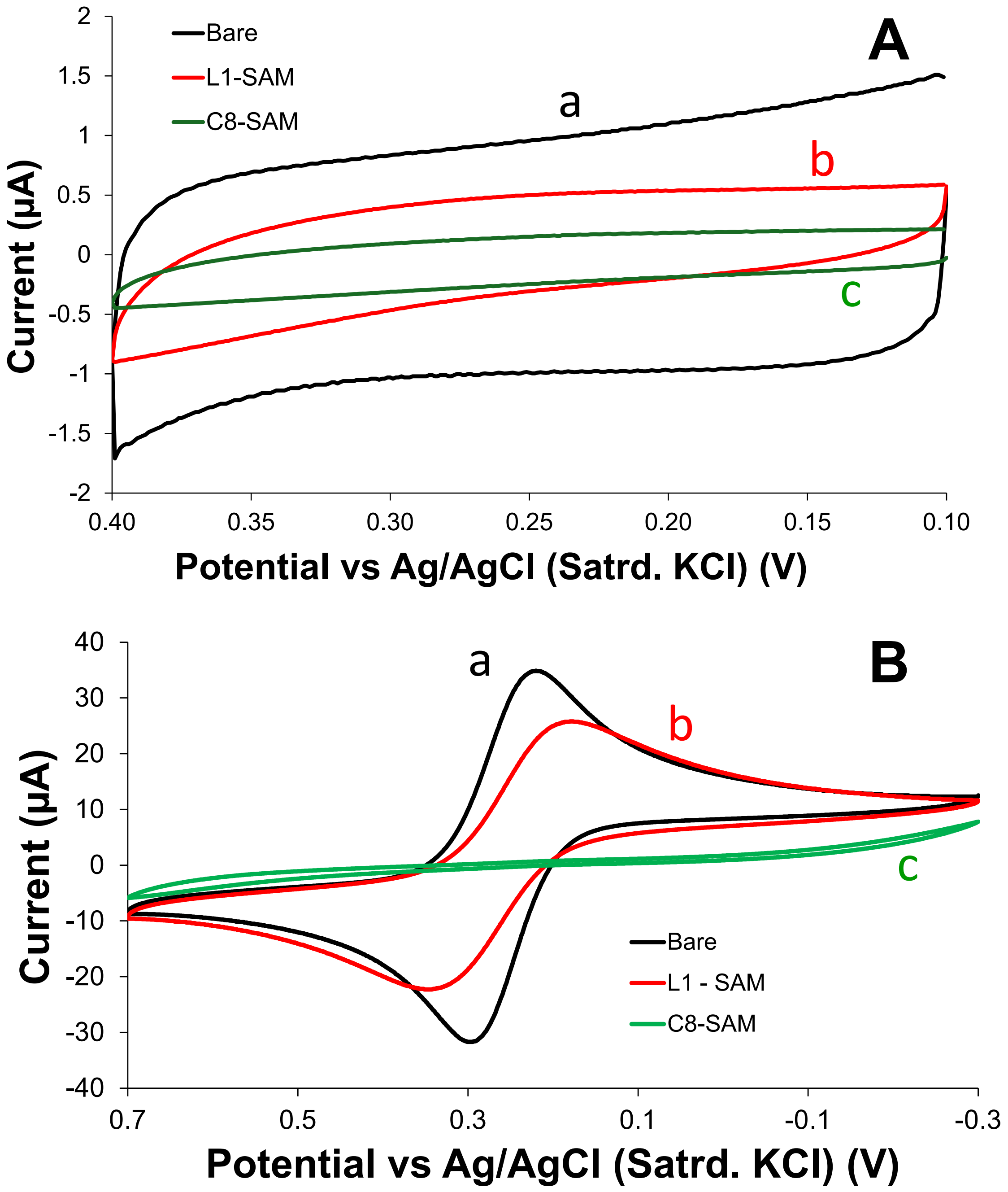

Figure SI-14. (A) Capacitance measurements using CV (4.4. mM PBS) of gold electrodes and (B) cyclic voltammetry of 5 mM FeCN (0.5 M KCl) AT **(a)** cleaned (unmodified) gold, (b) L1 SAM modified gold, and (c) C8 SAM modified gold substrates (included for comparison). Note: 100 mV/sec.

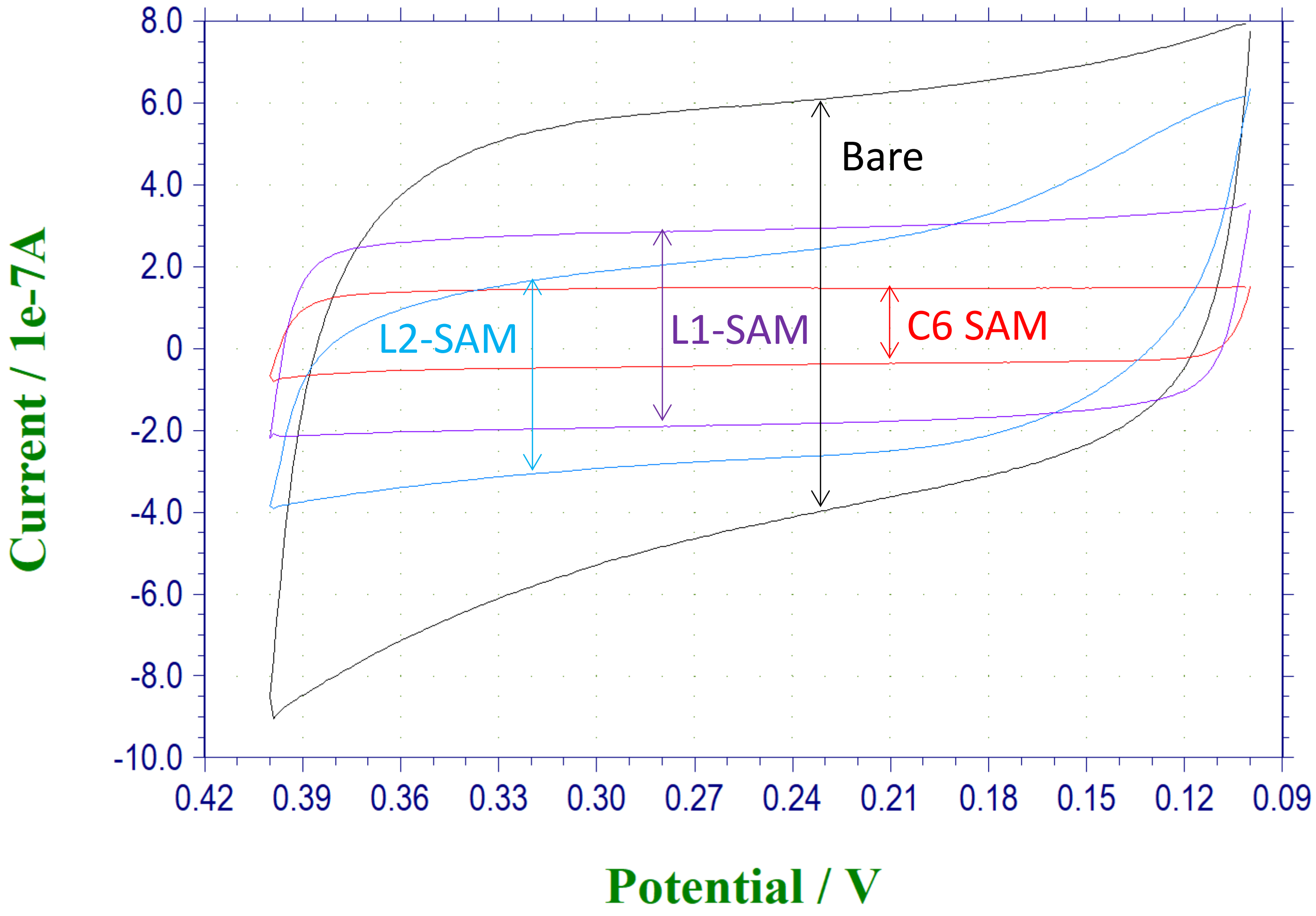

Figure SI-15. Cyclic voltammetry in 4.4 mM PBS (100 mV/sec) comparing C_{dl} magnitudes of SAM-modified gold with hexanethiol (C6), ligand 1 (L1), and ligand 2 (L2-I) versus bare gold.

Additional Details of DFT Analysis:

To assess the methodological dependence of our results, five different levels of theory were used (Table SI-2). to characterize the energies and geometries of dimer complexes of **L2** with each analyte shown in **Schemes 1 and 2**. A comparison of the results of these five different theoretical methods is shown in Supporting Information (Table SI-2). Gas phase results are relatively similar for the various approaches, with the methods M06-2X/cc-pVDZ//M06-2X/cc-pVTZ, wB97-XD/cc-pVDZ//wB97-XD/cc-pVTZ and wB97-XD/def2-TZVDP showing the most consistent results. The effect of solvent on the geometries and interaction energies was also examined and found that for 1-BP, CH, DABCO and DMDNB, solvent reduces the interaction energy between 8-45%. However, in the case of RDX and TNT (site 1 and 2) solvent increases the interaction energy by 11-13%. Interestingly, a decrease in interaction energy is not always accompanied by an increase in XB bond length, reflecting the complex interplay of **L2:analyte** dimer and monomer geometry optimization coupled with electronic and steric/geometric effects (Table SI-2).[6] For the remainder of this discussion, the focus will be the M06-2X/cc-pVDZ//M06-2X/cc-pVTZ results as data in both the gas and solvent (water) phase (**Table 1**) has been obtained. Results using B3LYP/cc-pVDZ, wB97-XD/def2-TZVDP and ωB97XD/cc-pVTZ//ωB97XD/cc-pVDZ can be found in the Supporting Information (Tables SI-3 to SI-7).

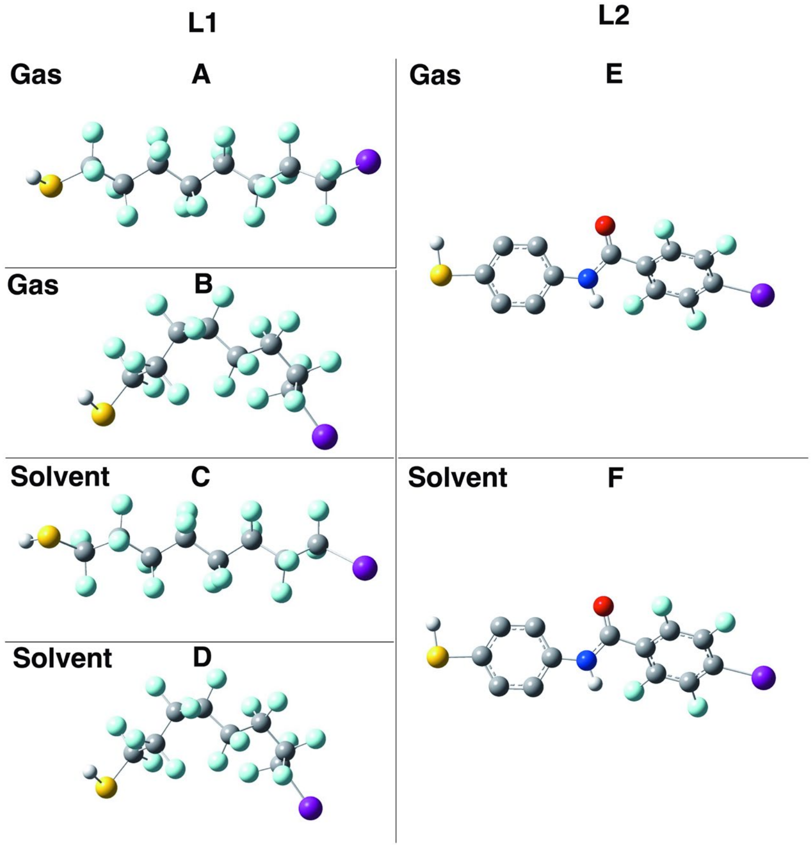

Figure SI-16. B3LYP/6-31G* geometry optimized structures for L1 (left) and L2 (right) in the gas (top) and aqueous phase (bottom). For L1, the bent conformation (B) lies 1.61 and 1.35 kcal/mol higher in energy than the extended structure in gas and solvent (water), respectively. For L2, the extended structure is the only conformation found in both gas and water.

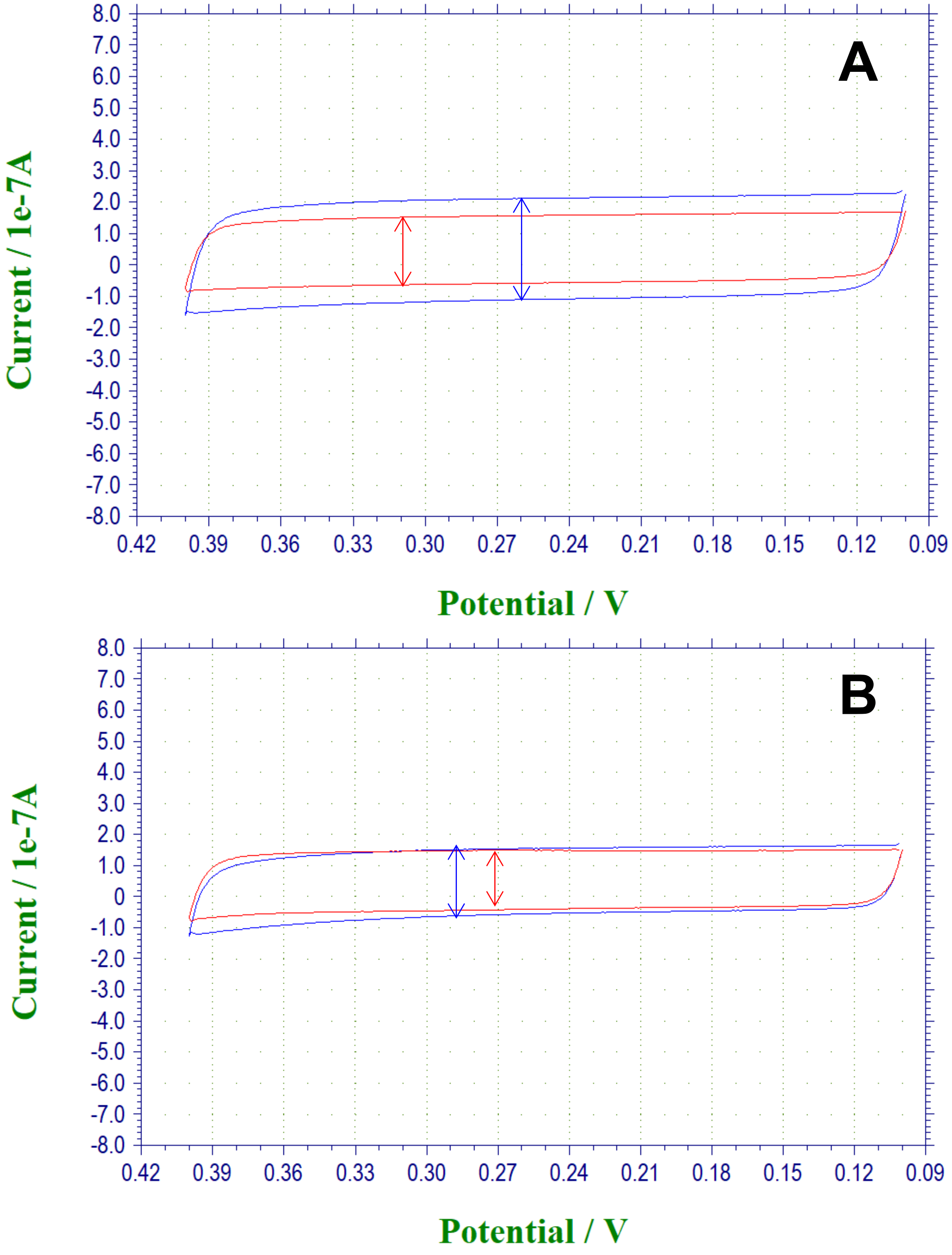

Figure SI-17. CV capacitance scans of C6 SAMs **before** and after exposure to **(A)** L1 and **(B)** L2 to create mixed SAMs. Small increases in the C_{dl} of the SAMs are noticed as a function of time exposed to these ligands as they exchange into the films.

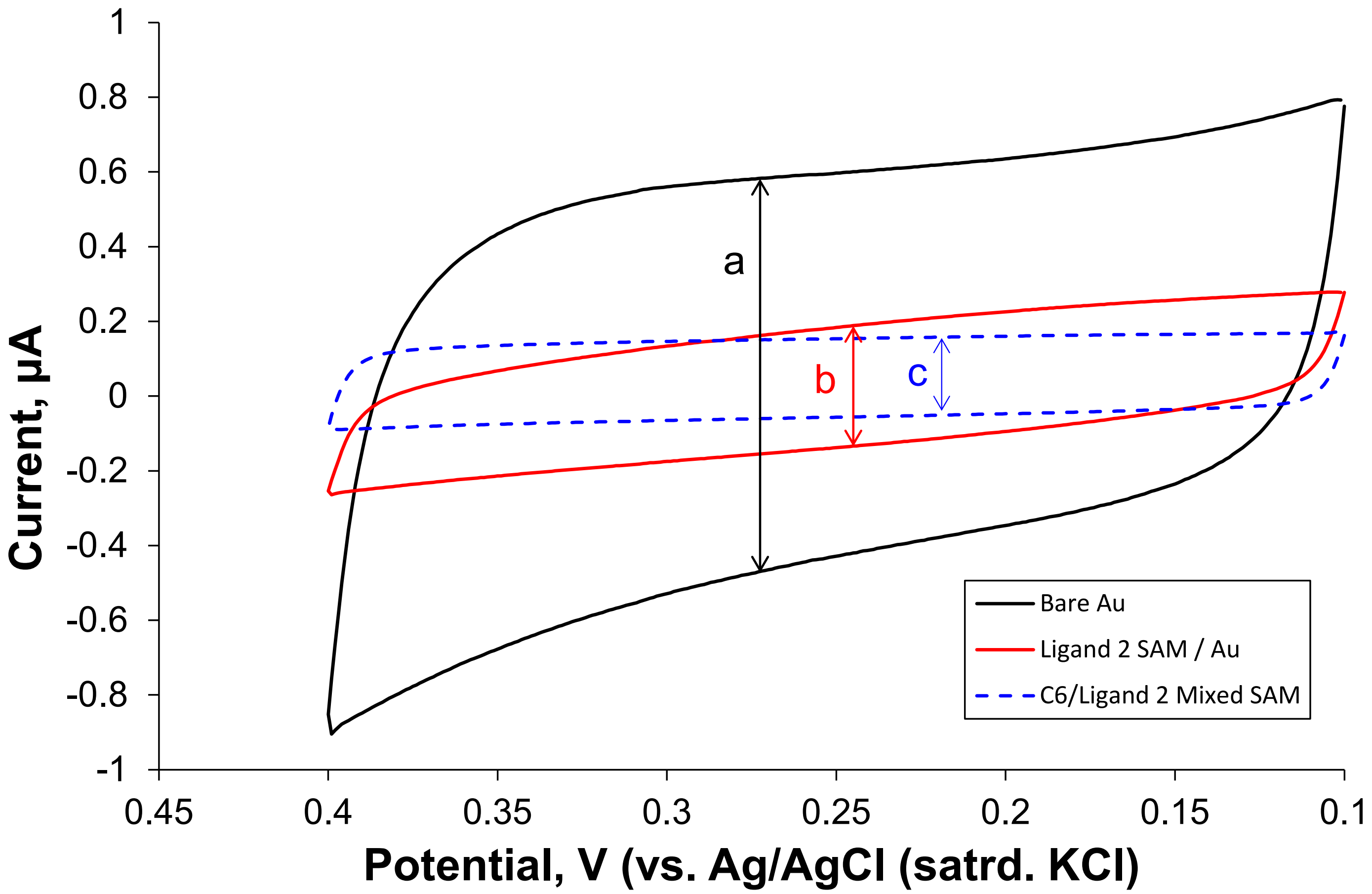

Figure SI-18. Capacitance measurements using CV (100 mV/sec; 4.4. mM PBS) of gold electrodes **(a)** cleaned (unmodified) and SAM-modified with either **(b)** ligand 2 (L2) and **(c)** C6/L2 mixed SAM.

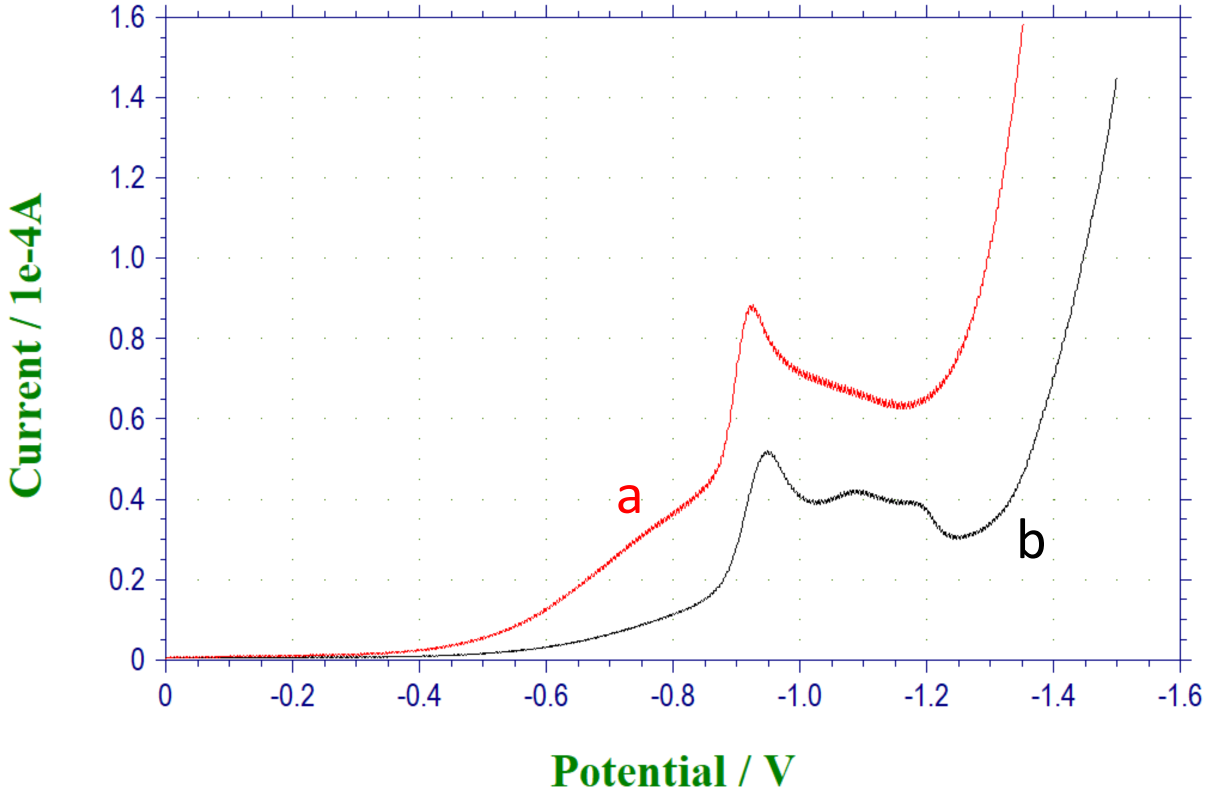

Figure SI-19 Linear sweep voltammetry (LSV) desorption of (a) L1 SAM versus (b) C6/L1 mixed SAM (0.1 M KOH). The emergence of multiple peaks with the mixed SAM suggests a successfully formed mixed SAM.

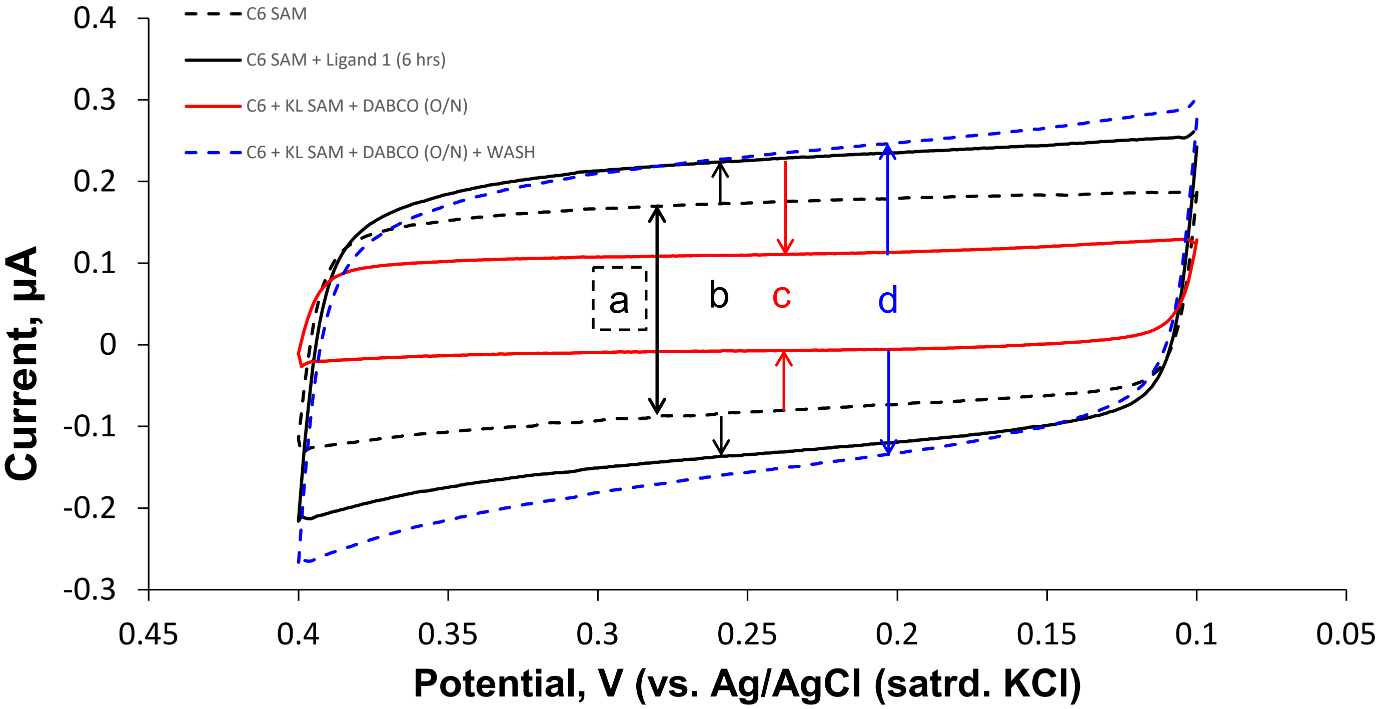

Figure SI-20. CV **c**apacitance measurements (100 mV/sec; 4.4. mM PBS) of a gold electrode **(a)** modified with a C6-SAM, **(b)** C6 SAM after extended exchange time to form a C6/L1 mixed SAM, and **(c)** the same mixed SAM after exposure to **DABCO** (12 hrs); and **(d)** after extensive washing with polar solvents.

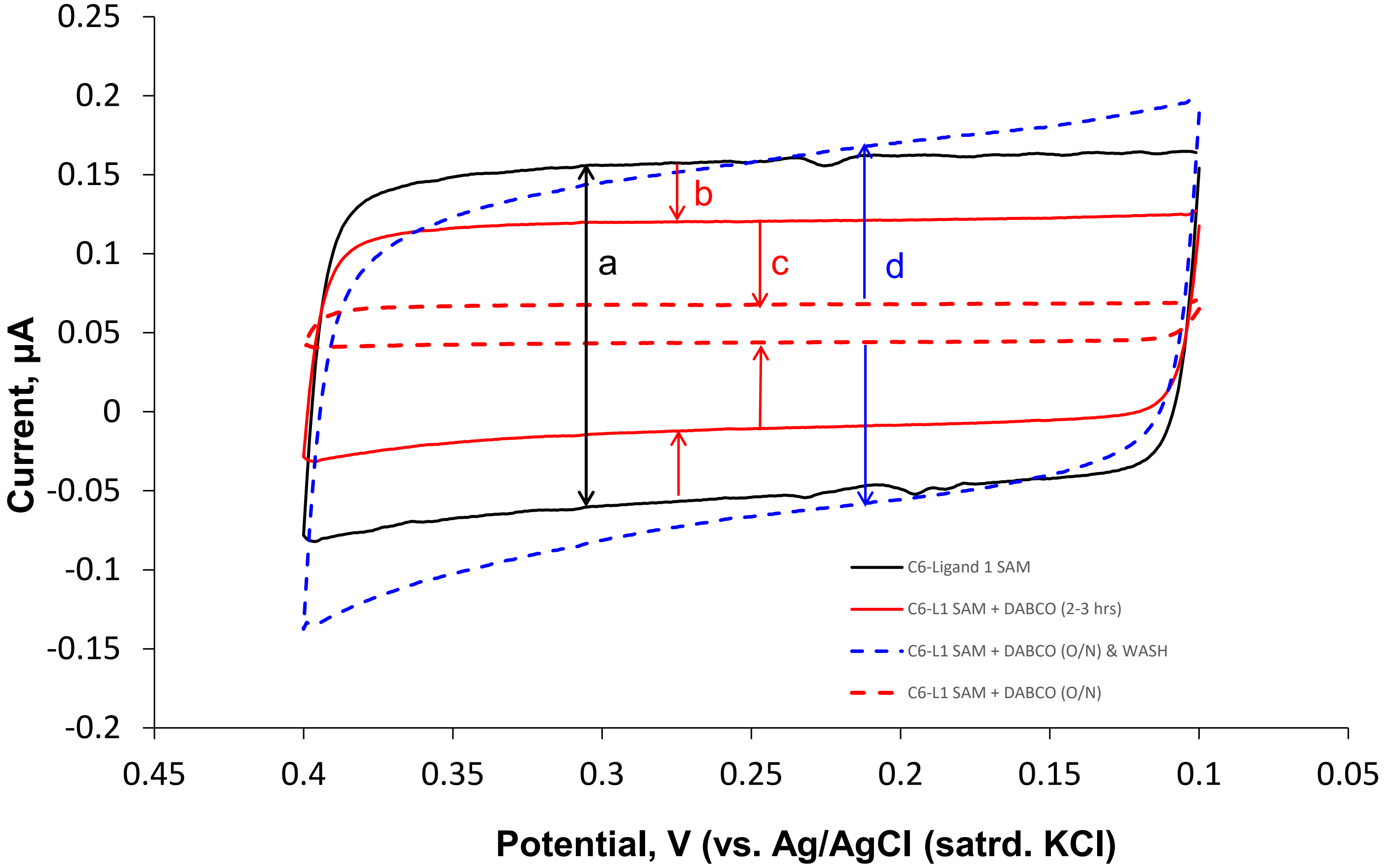

Figure SI-21. CV **c**apacitance measurements (100 mV/sec; 4.4. mM PBS) of a gold electrode **(a)** modified with a C6-SAM, **(b)** C6/L1 mixed SAM, and the same mixed SAM after (b) 2-3 hours and (c) 12 hours of exposure to **DABCO**; and **(d)** after extensive washing with polar solvents.

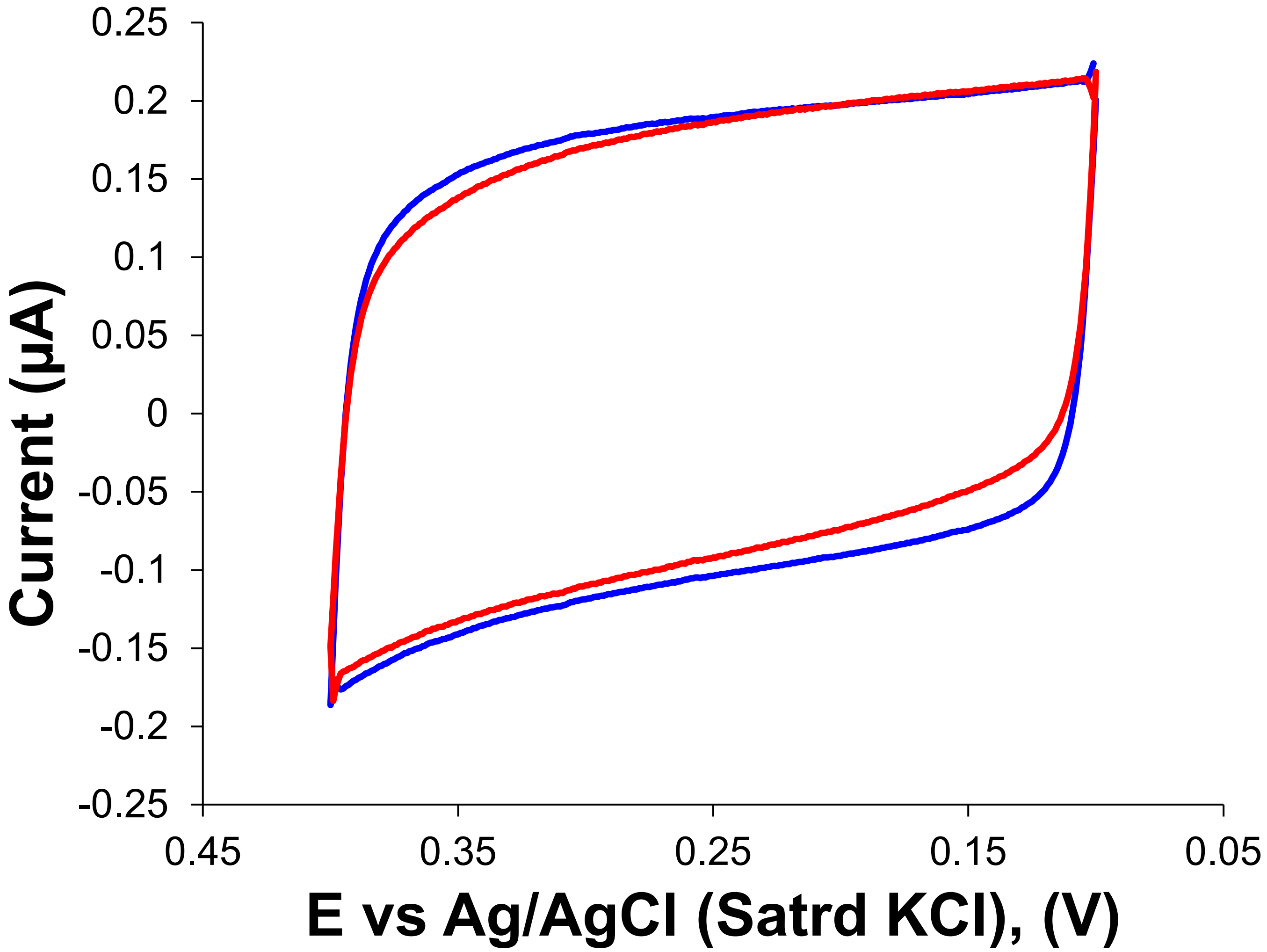

Figure SI-22. Capacitance measurements using CV (100 mV/sec; 4.4. mM PBS) of gold electrodes modified with hexanthiolate (C6) and 11-mercaptoundecanoic acid (MUA) mixed SAM before **(a)** and after **(b)** exposure to **DABCO** (12 hours) and showing very little change in C_{dl}.

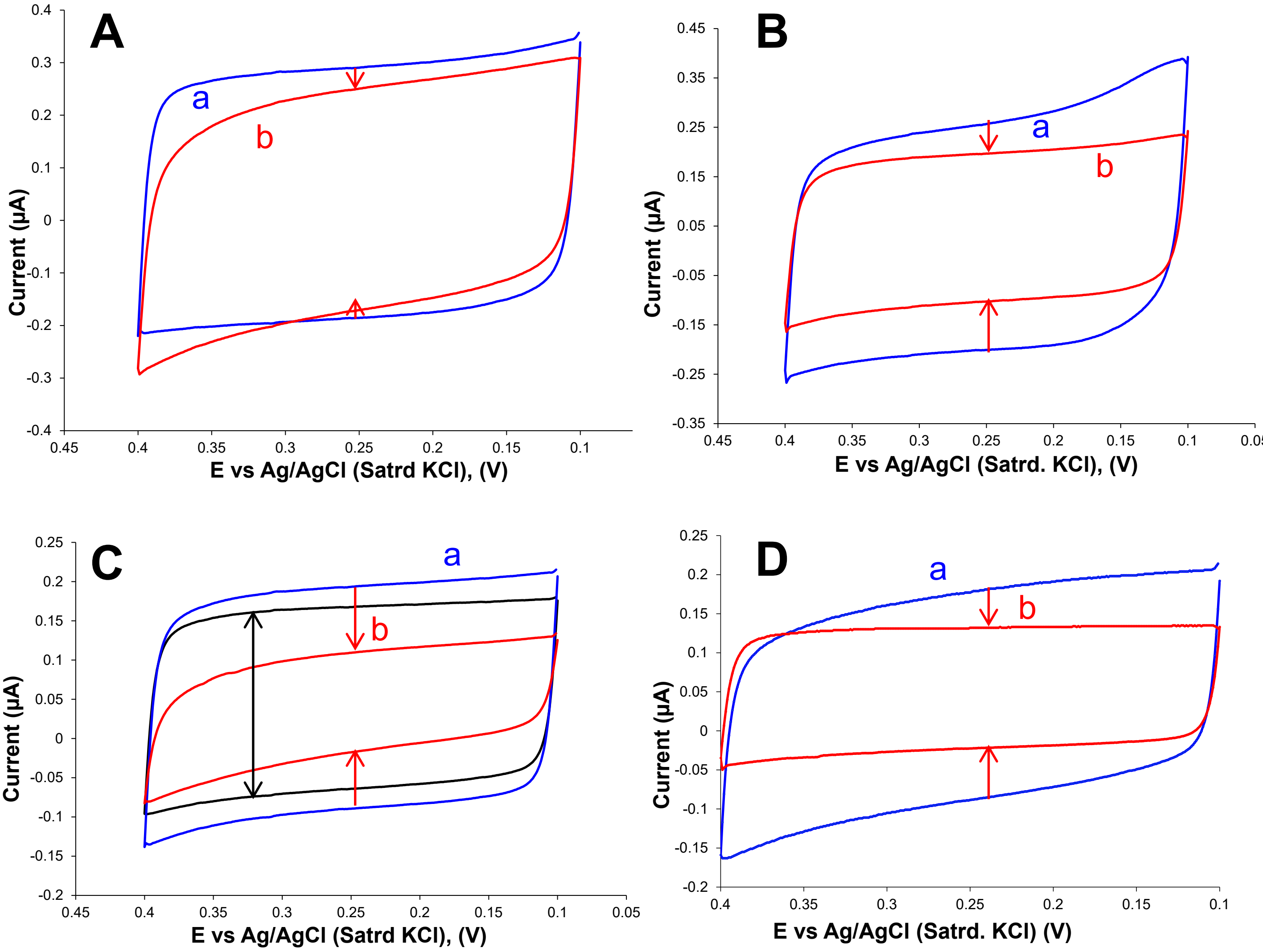

Figure SI-23. Representative CV (100 mV/sec; 4.4. mM PBS) reflecting C_{dl} of gold electrodes modified with **(A)** uniform L1 SAM, **(B)** uniform L2 SAM, **(C)** mixed C6/L1 SAM, and **(D)** mixed C6/L2 SAM before **(a)** and after **(b)** exposure to the XB acceptor **1-BP** (12 hours). Note: Scans in panel C also include the original C6 SAM capacitance prior to incorporating L1 to create the mixed SAM capacitance (blue).

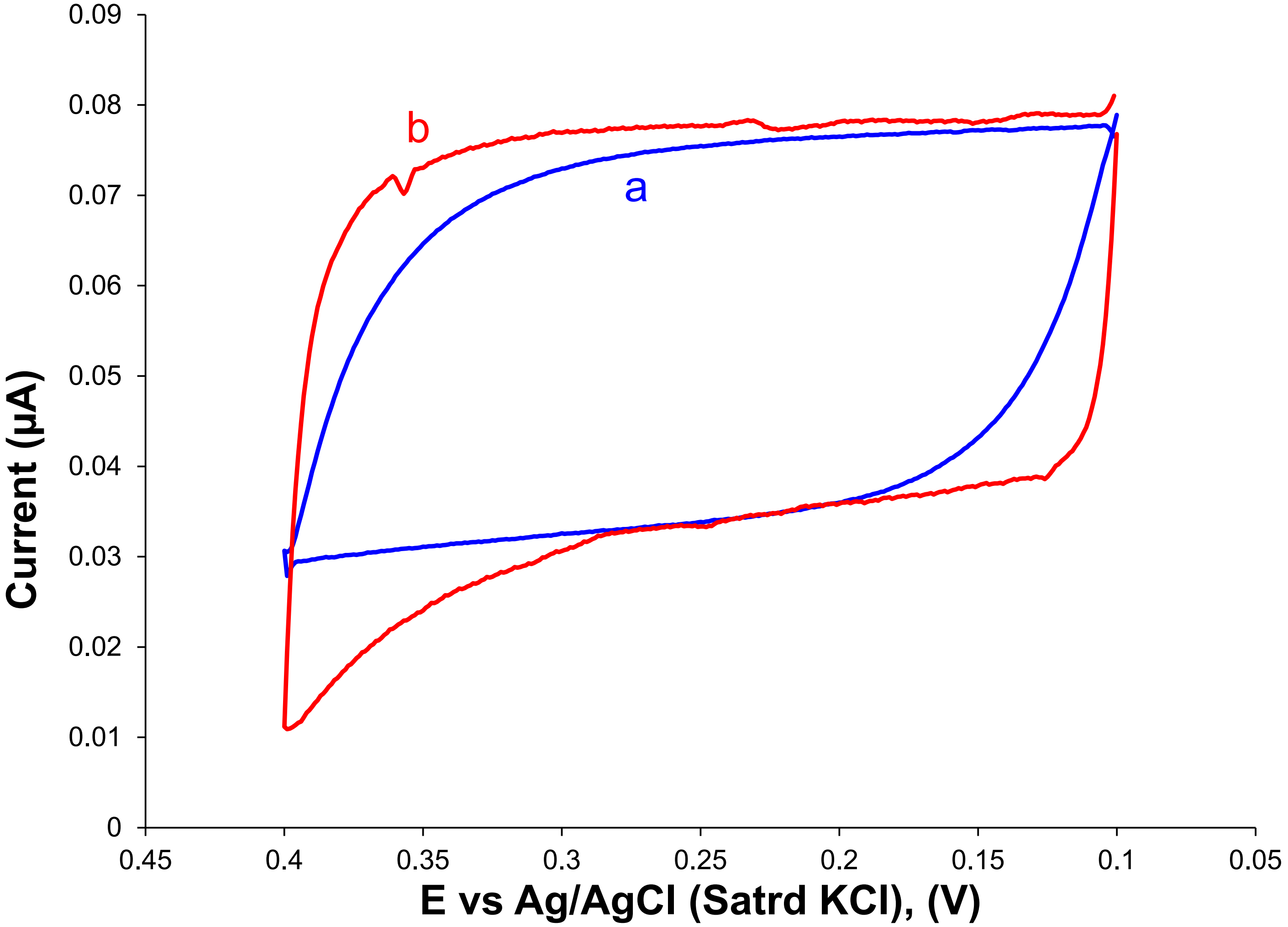

Figure SI-24. Capacitance measurements using CV (100 mV/sec; 4.4. mM PBS) of gold electrodes modified with C6 and decanethiol (C10) mixed SAM before **(a)** and after **(b)** exposure to 1-BP (12 hours) and showing very little change in C_{dl}.

Table SI-0. Double layer capacitance (C_{dl}) values for various modified electrode systems measured before and after exposure to XB acceptors (1-BP and DABCO).

Modified Electrode System	Initial Film (μF/cm²)	After 1-BP (μF/cm²)	After DABCO (μF/cm²)
**L1-SAM**	7.01 (±0.36)	6.37 (±0.20)	5.48 (±0.11)
**L2-SAM**	7.44 (±0.81)	5.03 (±0.81)	5.92 (±0.50)
**C6-L2 Mixed SAM**	3.62 (±0.25)	2.08 (±0.35)	1.95 (±0.23)
**C6-L1 Mixed SAM**	5.65 (±0.14)	3.55 (±0.55)	1.88 (±0.10)
**L2-fMPC**	6.43 (±0.71)	2.39 (±0.13)	2.81 (*±35*)
**L1-xchng MPC**	5.34 (±0.55)	2.86 (±0.70)	1.94 (±0.19)
**Unf-MPC (control)**	3.86 (±1.03)	3.48 (±0.42)	4.40 (±0.61)

NOTES: Uncertainty represents standard error (n = 3-7). C6 is hexanthiol; unf-MPC are MPCs protected only with C6 ligands and without XB donor capability.

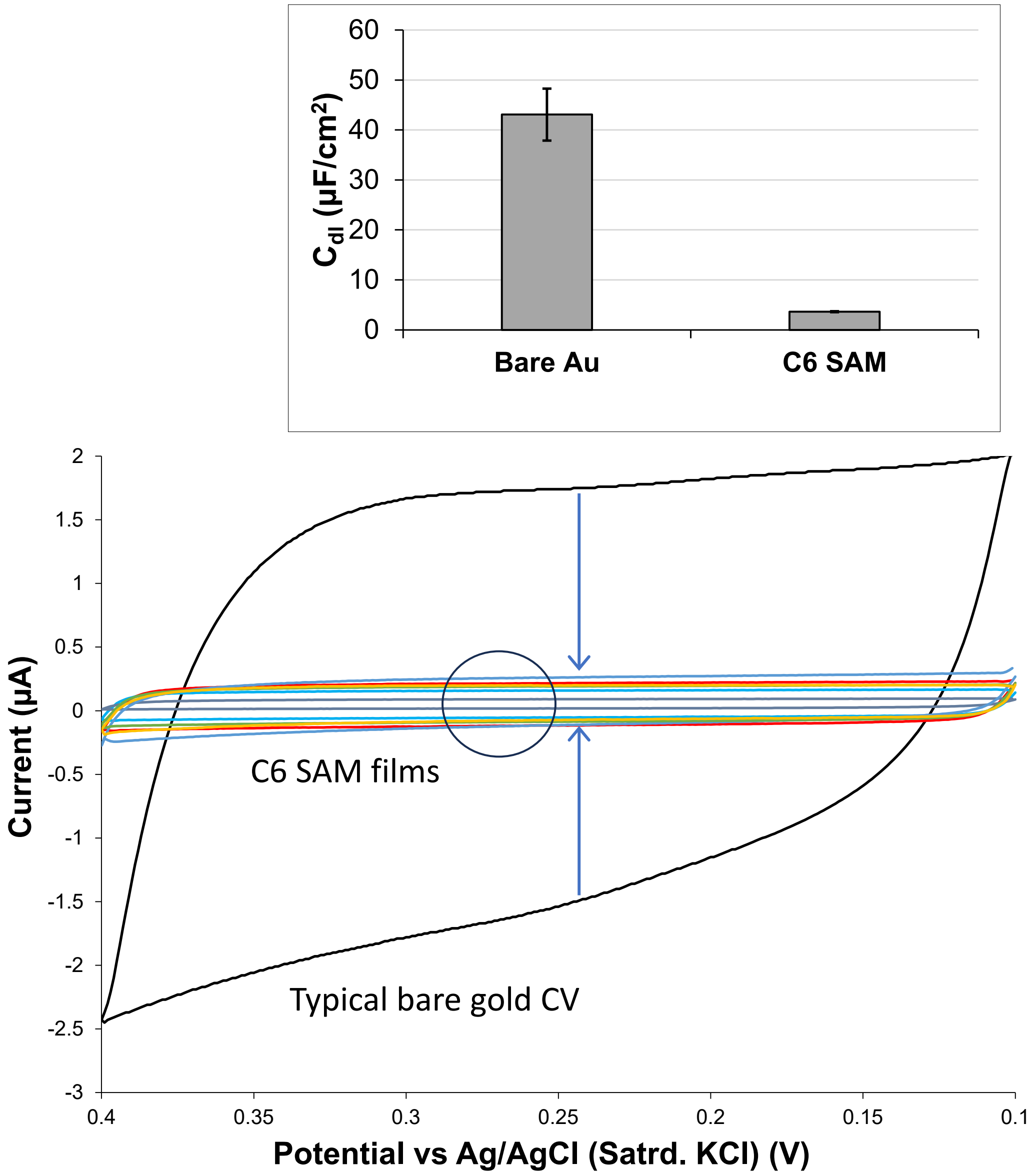

Figure SI-25. Typical CV capacitance measurements(100 mV/sec; 4.4. mM PBS) of gold electrodes **(a)** cleaned (unmodified) and C6 SAM-modified prior to assembling UDDT linked MPC films. In all cases, C6-SAMs were verified as were the assembly of MPC material at the interfaces.

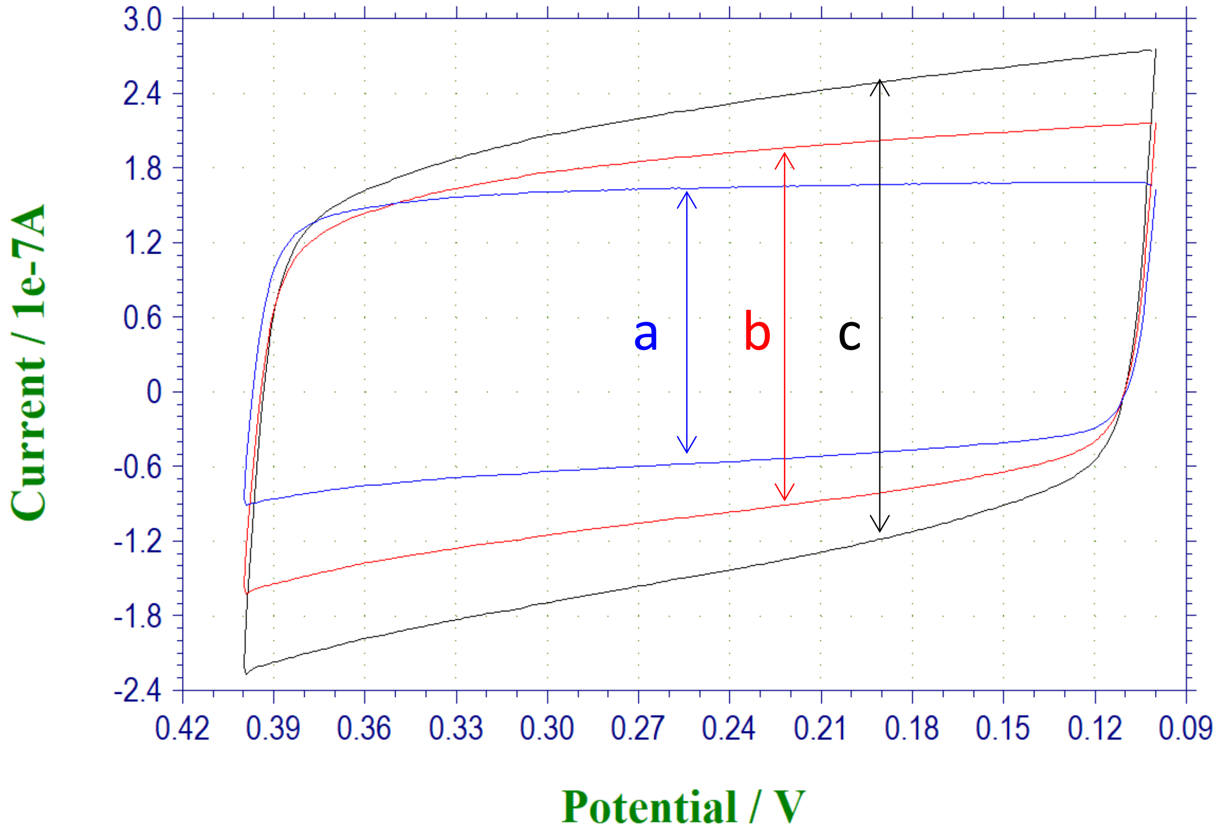

Figure SI-26. Capacitance measurements using CV (100 mV/sec; 4.4. mM PBS) of gold electrodes modified with (a) C6 SAM versus after (b) first exposure and after (c) second exposure of undecane dithiol (UDDT)-linked (15 min) f-MPC (L2) layers (1 hour).

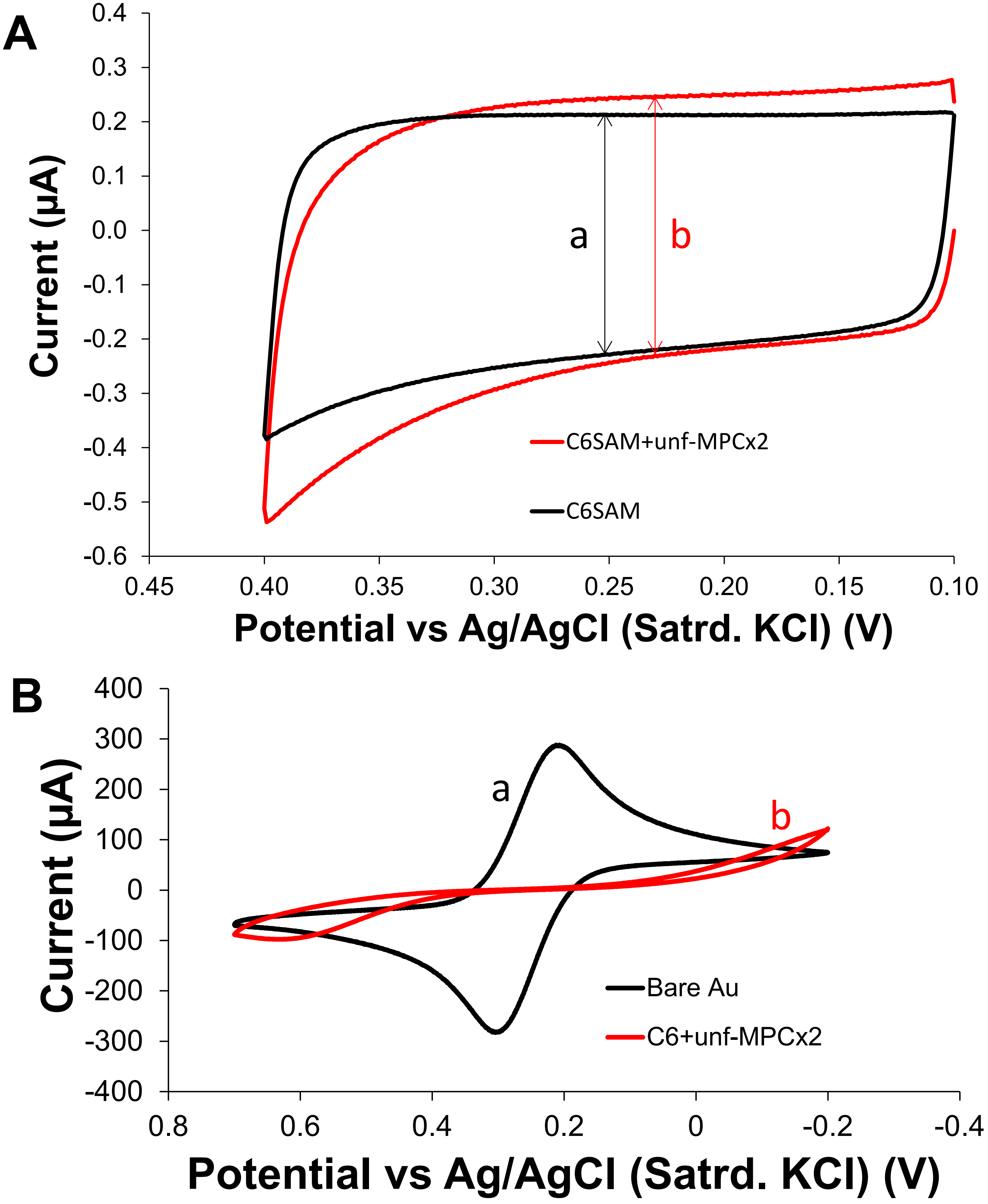

Figure SI-27. (A) Capacitance measurements using CV (100 mV/sec; 4.4. mM PBS) of gold electrodes modified with (a) C6 SAM versus (b) after two exposures to nonane-thiol-linked (15 min) unf-MPC material (1 hour) and; (B) cyclic voltammetry of 5 mM FeCN (0.5 M KCl) at (a) bare gold versus (b) a C6 SAM subsequently modified with two exposures to NDT-linked (15 min) unf-MPC material (1 hour) .

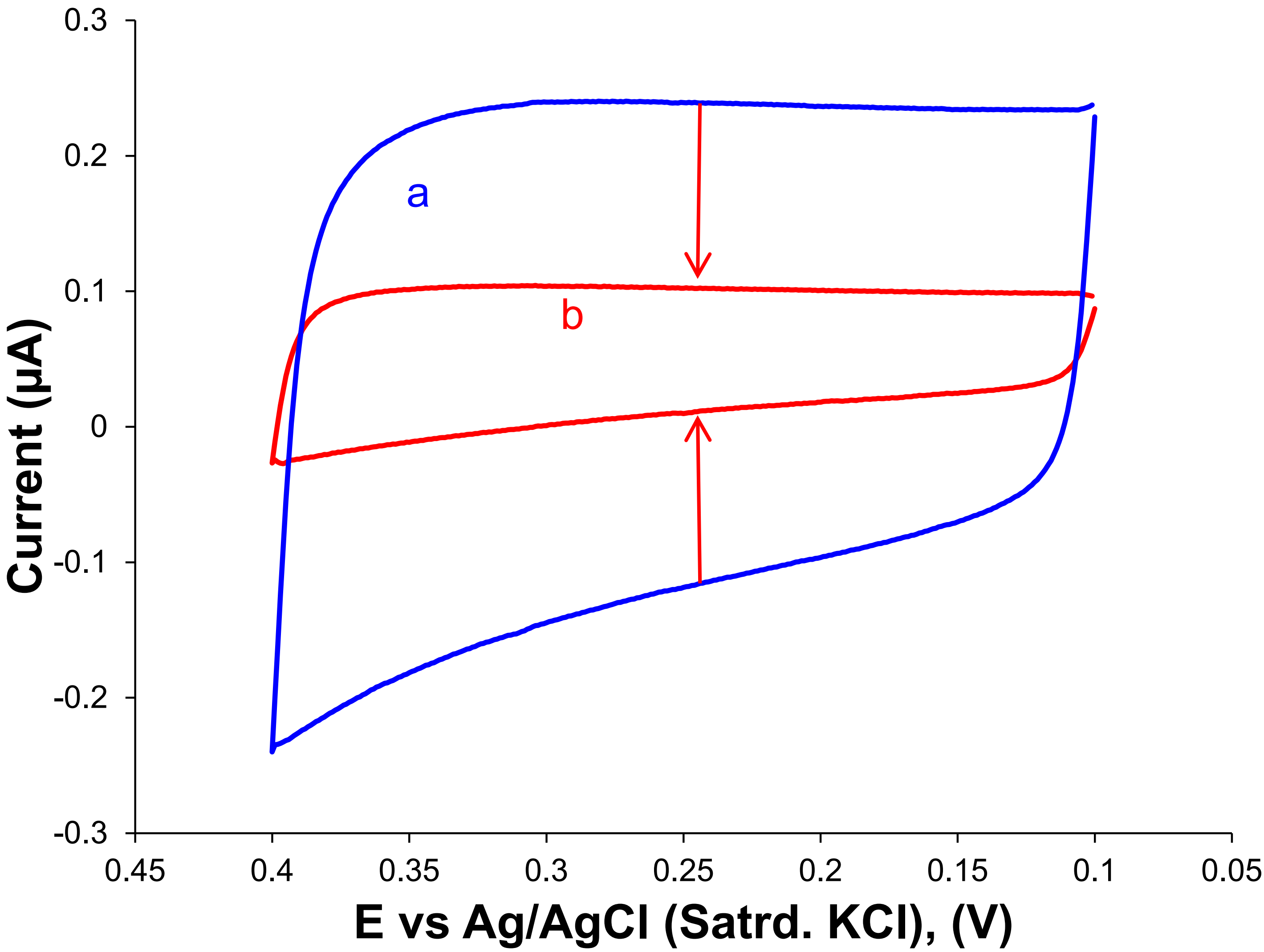

Figure SI-28. Capacitance measurements using CV (100 mV/sec; 4.4. mM PBS) of gold electrodes modified with C6 SAM and layers of f-MPCs (L1) before **(a)** and after **(b)** exposure to DABCO (12 hours).

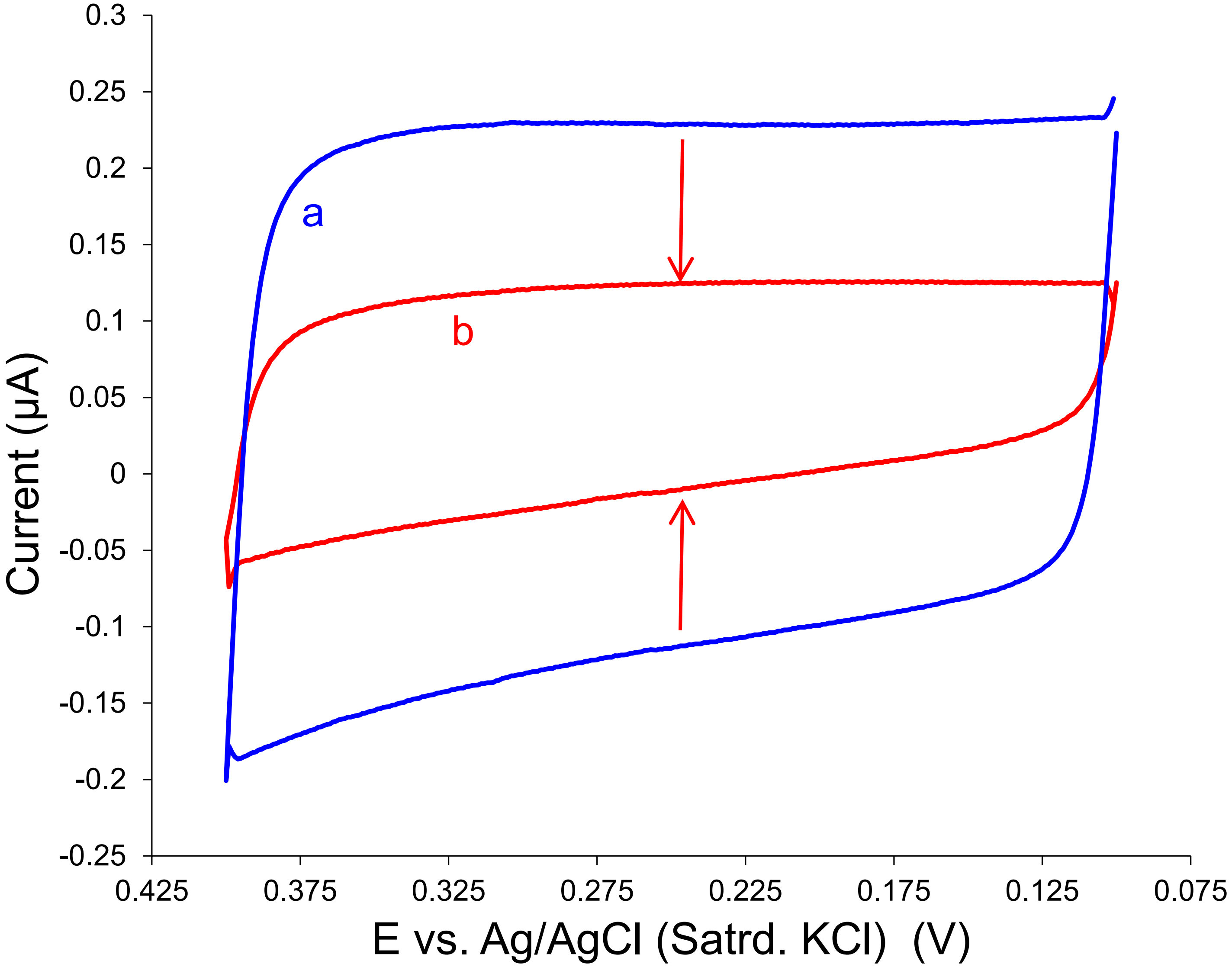

Figure SI-29. Capacitance measurements using CV (100 mV/sec; 4.4. mM PBS) of gold electrodes modified with C6 SAM and layers of f-MPCs (L1) before **(a)** and after **(b)** exposure to 1-BP (12 hours).

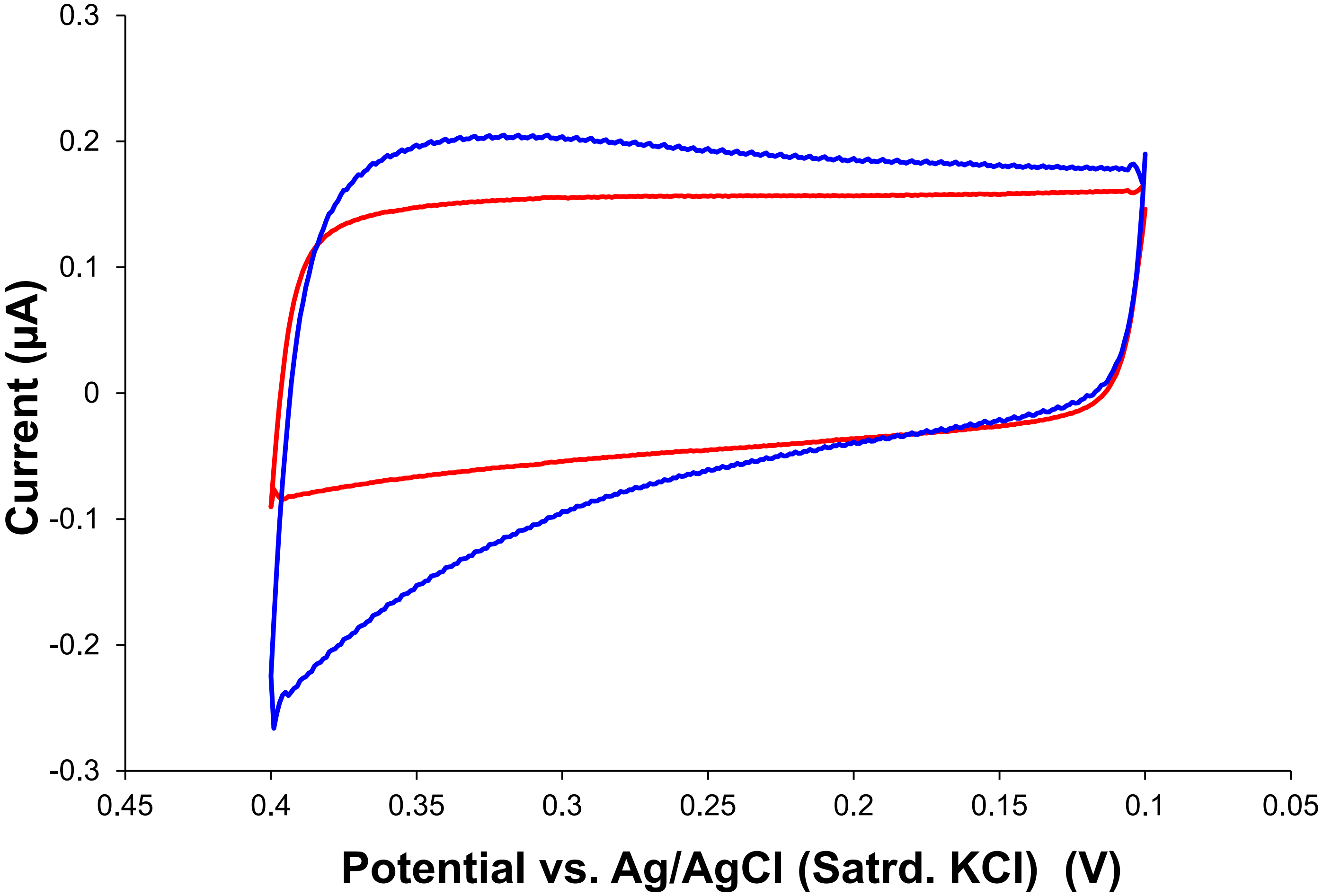

Figure SI-30. Capacitance measurements using CV (100 mV/sec; 4.4. mM PBS) of gold electrodes modified with C6 SAM and layers of unf-MPCs before **(a)** and after **(b)** exposure to 1-BP (12 hours) and showing very little change in C_{dl}.

Table SI-2. Interaction Energies (ΔE_{int}) and geometries between L2 and various analytes.

Method	BE (kcal/mol)	X---B distance (A)	R-X---B angle (°)
**1-BP**			
B3LYP/cc-pVDZ (gas)	-4.63	2.94	175.57
MO6-2X/cc-pVDZ//MO6-2X/cc-pVTZ (gas)	-8.72	2.84	179.09
ωB97X-D/cc-pVDZ//wB97X-D/cc-pVTZ (gas)	-8.46	2.89	177.48
ωB97X-D/def2-TZVDP (gas)	-8.39	2.95	174.99
MO6-2X/cc-pVDZ (water)	-5.81	2.74	177.90
**Cyclohexanone**			
B3LYP/cc-pVDZ (gas)	-5.87	2.89	174.30
MO6-2X/cc-pVDZ//MO6-2X/cc-pVTZ (gas)	-6.68	2.87	179.30
ωB97X-D/cc-pVDZ//wB97X-D/cc-pVTZ (gas)	-9.88	2.87	178.67
ωB97X-D/def2-TZVDP (gas)	-9.57	2.94	178.30
ωB97-MV/def2-TZVDP (gas)	-10.13	2.90	178.77
MO6-2X/cc-pVDZ (water)	-4.22	2.88	176.39
**DABCO**			
B3LYP/cc-pVDZ (gas)	-8.33	2.83	179.75
MO6-2X/cc-pVDZ//MO6-2X/cc-pVTZ (gas)	-10.00	2.77	179.77
ωB97X-D/cc-pVDZ//wB97X-D/cc-pVTZ (gas)	-9.66	2.81	179.79
ωB97X-D/def2-TZVDP (gas)	-9.17	2.84	179.63
MO6-2X/cc-pVDZ (water)	-5.49	2.68	179.70
**DMNB**			
B3LYP/cc-pVDZ (gas)	-4.58	3.04	174.30
MO6-2X/cc-pVDZ//MO6-2X/cc-pVTZ (gas)	-5.16	2.97	171.77
ωB97X-D/cc-pVDZ//wB97X-D/cc-pVTZ (gas)	-4.51	3.02	166.59
ωB97X-D/def2-TZVDP (gas)	-4.08	3.16	163.61
MO6-2X/cc-pVDZ (water)	-4.73	2.98	173.65
**RDX**			
B3LYP/cc-pVDZ (gas)	-3.27	3.09	175.00
MO6-2X/cc-pVDZ//MO6-2X/cc-pVTZ (gas)	-3.76	3.02	174.79
ωB97X-D/cc-pVDZ//wB97X-D/cc-pVTZ (gas)	-2.87	3.07	174.89
ωB97X-D/def2-TZVDP (gas)	N/A	N/A	N/A
MO6-2X/cc-pVDZ (water)	-4.24	3.00	174.63
**TNT site 1**			
B3LYP/cc-pVDZ (gas)	-3.74	3.09	174.38
MO6-2X/cc-pVDZ//MO6-2X/cc-pVTZ (gas)	-4.03	3.03	175.10
ωB97X-D/cc-pVDZ//wB97X-D/cc-pVTZ (gas)	-3.20	3.06	174.83
ωB97X-D/def2-TZVDP (gas)	N/A	N/A	N/A
MO6-2X/cc-pVDZ (water)	-4.51	3.01	173.65
**TNT site 2**			
B3LYP/cc-pVDZ (gas)	-3.81	3.08	175.02
MO6-2X/cc-pVDZ//MO6-2X/cc-pVTZ (gas)	-4.10	3.02	175.33
ωB97X-D/cc-pVDZ//wB97X-D/cc-pVTZ (gas)	-3.13	3.07	175.27
ωB97X-D/def2-TZVDP (gas)	-2.60	3.19	175.76
MO6-2X/cc-pVDZ (water)	-4.53	3.02	173.87

Table SI-3. B3LYP/cc-pVDZ Interaction Energies (ΔE_{int}), Bond Distances (X---B), and Bond Angles of XB Adducts. The sum of The Van Der Waal Radii for Iodine and Oxygen is 3.5 Å [b]

XB Acceptors	ΔE_{int} (kcal/mol)[a]	X---B distance (Å)	R-X---B angle (degrees)
1-BP	-4.63	2.94	175.57
DABCO	-8.33	2.83	179.75
Dimethlydinitrobutane	-4.58	3.04	174.30
RDX	-3.27	3.09	175.00
TNT site 1	-3.74	3.09	174.38
TNT site 2	-3.81	3.08	175.02
Cyclohexanone	-5.87	2.89	174.30

Notes: [a] ΔE_{int} = E(complex) – [E(XB donor) + E(XB acceptor)]. [b] (Iodine $radius_{VDW}$ + Oxygen $radius_{VDW}$).

Table SI-4. M06-2x/cc-pVTZ// M06-2x/cc-pVDZ Interaction Energies (ΔE_{int}), Bond Distances (X---B), and Bond Angles of XB Adducts. The sum of The Van Der Waal Radii for Iodine and Oxygen is 3.5 Å [b]

XB Acceptors	ΔE_{int} (kcal/mol)[a]	X---B distance (Å)	R-X---B angle (degrees)
1-BP	-8.72	2.84	179.09
DABCO	-10.00	2.77	179.77
Dimethlydinitrobutane	-5.16	2.97	171.77
RDX	-3.76	3.02	174.79
TNT site 1	-4.03	3.03	175.10
TNT site 2	-4.10	3.02	175.33
Cyclohexanone	-6.68	2.87	179.30

Notes: [a] ΔE_{int} = E(complex) – [E(XB donor) + E(XB acceptor)]. [b] (Iodine $radius_{VDW}$ + Oxygen $radius_{VDW}$).

Table SI-5. ωB97X-D/cc-pVTZ//ωB97X-D/cc-pVDZ Interaction Energies (ΔE_{int}), Bond Distances (X-B), and Bond Angles of XB Adducts. The Sum of the Van Der Waal Radii for Iodine and Oxygen is 3.5 Å [b].

XB Acceptors	ΔE_{int} (kcal/mol)[a]	X---B distance (Å)	R-X---B angle (degrees)
1-BP	-8.46	2.88	177.48
DABCO	-9.66	2.81	179.79
Dimethlydinitrobutane	-4.51	3.02	166.59
RDX	-2.87	3.07	174.89
TNT site 1	-3.20	3.07	174.83
TNT site 2	-3.13	3.07	175.27
Cyclohexanone	-9.88	2.87	178.67

Notes: [a] ΔE_{int} = E(complex) – [E(XB donor) + E(XB acceptor)]. [b] (Iodine $radius_{VDW}$ + Oxygen $radius_{VDW}$).

Table SI-6. M06-2x/cc-pVDZ Interaction Energies (ΔE_{int}), Bond Distances (X---B), and Bond Angles of XB Adducts calculated in solvent water. The sum of The Van Der Waal Radii for Iodine and Oxygen is 3.5 Å [b].

XB Acceptors	ΔE_{int} (kcal/mol)[a]	X---B distance (Å)	R-X---B angle (degrees)
1-BP	-5.81	2.74	177.90
DABCO	-5.49	2.68	179.70
Dimethlydinitrobutane	-4.73	2.98	173.65
RDX	-4.24	3.00	174.63
TNT site 1	-4.51	3.01	173.65
TNT site 2	-4.53	3.03	173.87
Cyclohexanone	-4.22	2.88	176.39

Notes: [a] ΔE_{int} = E(complex) – [E(XB donor) + E(XB acceptor)]. [b] (Iodine $radius_{VDW}$ + Oxygen $radius_{VDW}$).

Table SI-7. ωB97X-D/def2-STVDP interaction Energies (ΔE_{int}), Bond Distances (X---B), and Bond Angles of XB Adducts calculated in solvent water. The sum of The Van Der Waal Radii for Iodine and Oxygen is 3.5 Å [b].

XB Acceptors	ΔE_{int} (kcal/mol)[a]	X---B distance (Å)	R-X---B angle (degrees)
1-BP	-8.39	2.95	174.99
DABCO	-9.17	2.84	179.63
Dimethlydinitrobutane	-4.08	3.16	163.61
TNT site 2	-2.61	3.19	175.76
Cyclohexanone	-9.57	2.94	178.30

Notes: [a] ΔE_{int} = E(complex) – [E(XB donor) + E(XB acceptor)]. [b] (Iodine $radius_{VDW}$ + Oxygen $radius_{VDW}$).

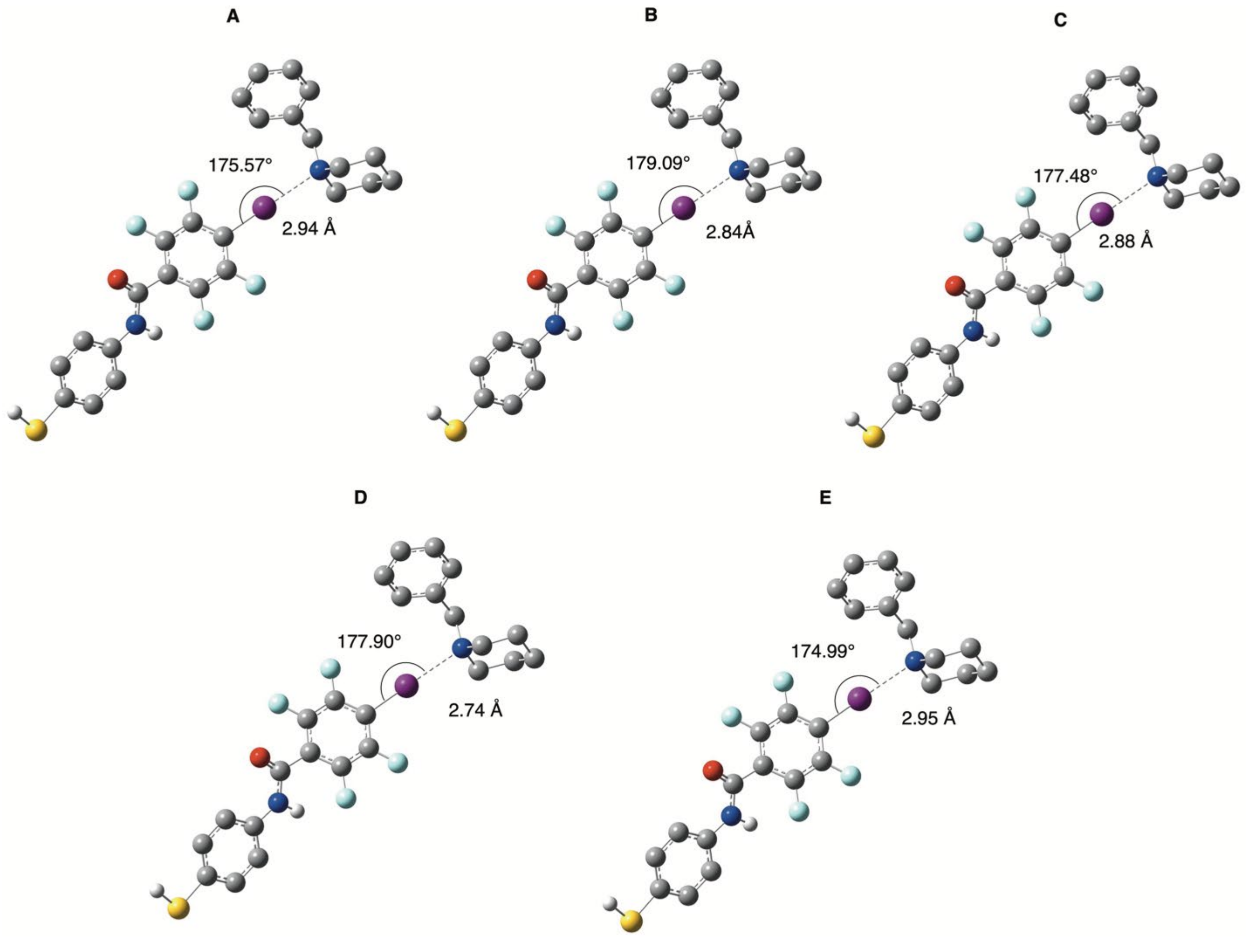

Figure SI-31. Geometry optimizations of the 1-BP - L2 interaction complex at three levels of theory A) B3LYP/cc-pVDZ (Gas), B) M06-2x/cc-pVDZ//M06-2x/cc-pVTZ (Gas), C) ωB97X-D/cc-pVDZ// ωB97X-D/cc-pVTZ (Gas), D) M06-2x/cc-pVDZ//M06-2x/cc-pVTZ (Solvent = water), E) ωB97X-D/def2-STVDP (Gas).

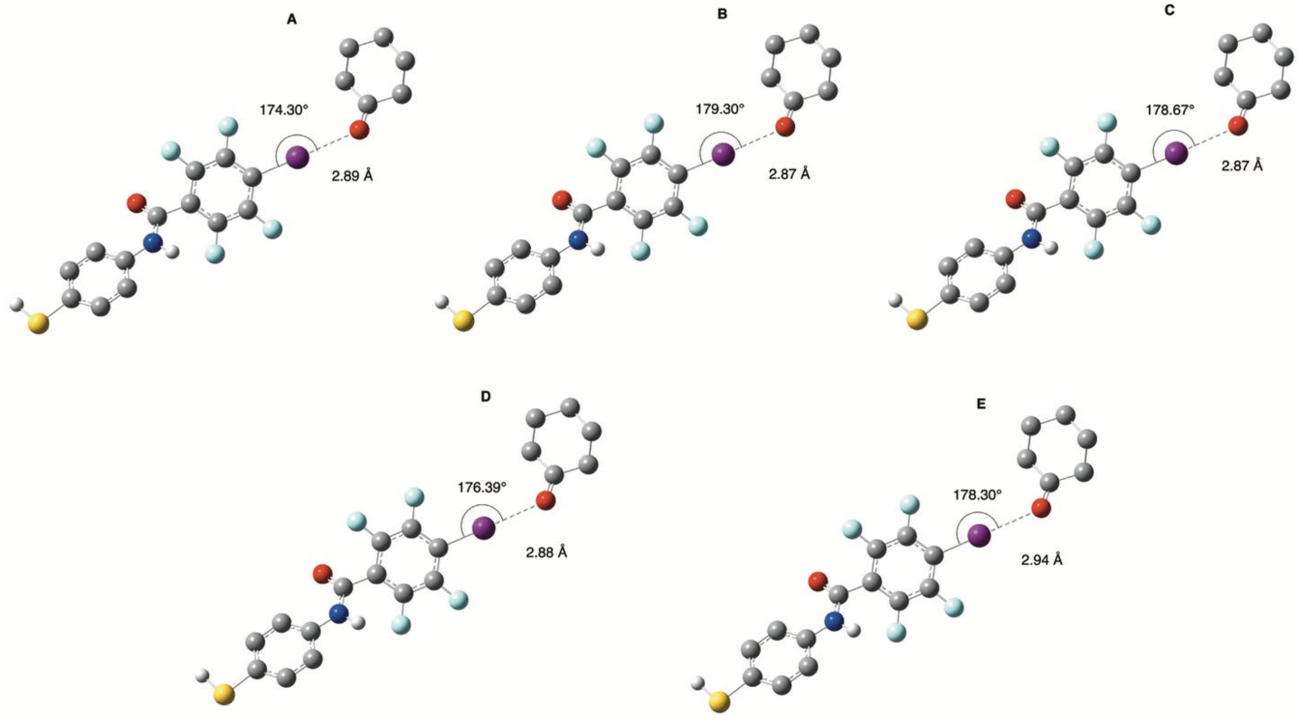

Figure SI-32. Geometry optimizations of the Cyclohexanone - L2 interaction complex at three levels of theory A) B3LYP/cc-pVDZ (Gas), B) M06-2x/cc-pVDZ//M06-2x/cc-pVTZ (Gas), C) ωB97X-D/cc-pVDZ//ωB97X-D/cc-pVTZ (Gas), D) M06-2x/cc-pVDZ//M06-2x/cc-pVTZ (Solvent = water), E) ωB97X-D/def2-STVDP (Gas).

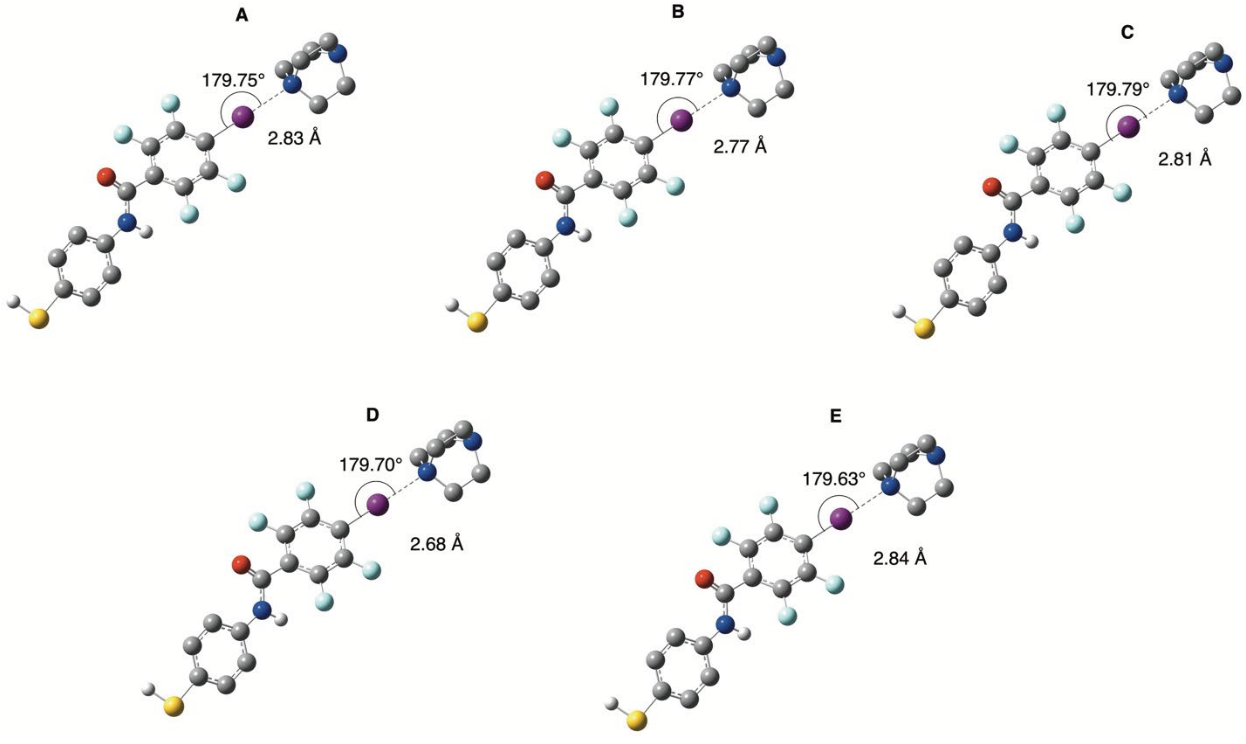

Figure SI-33. Geometry optimizations of the DABCO - L2 interaction complex at three levels of theory A) B3LYP/cc-pVDZ (Gas), B) M06-2x/cc-pVDZ//M06-2x/cc-pVTZ (Gas), C) ωB97X-D/cc-pVDZ//ωB97X-D/cc-pVTZ (Gas), D) M06-2x/cc-pVDZ//M06-2x/cc-pVTZ (Solvent = water), E) ωB97X-D/def2-STVDP (Gas).

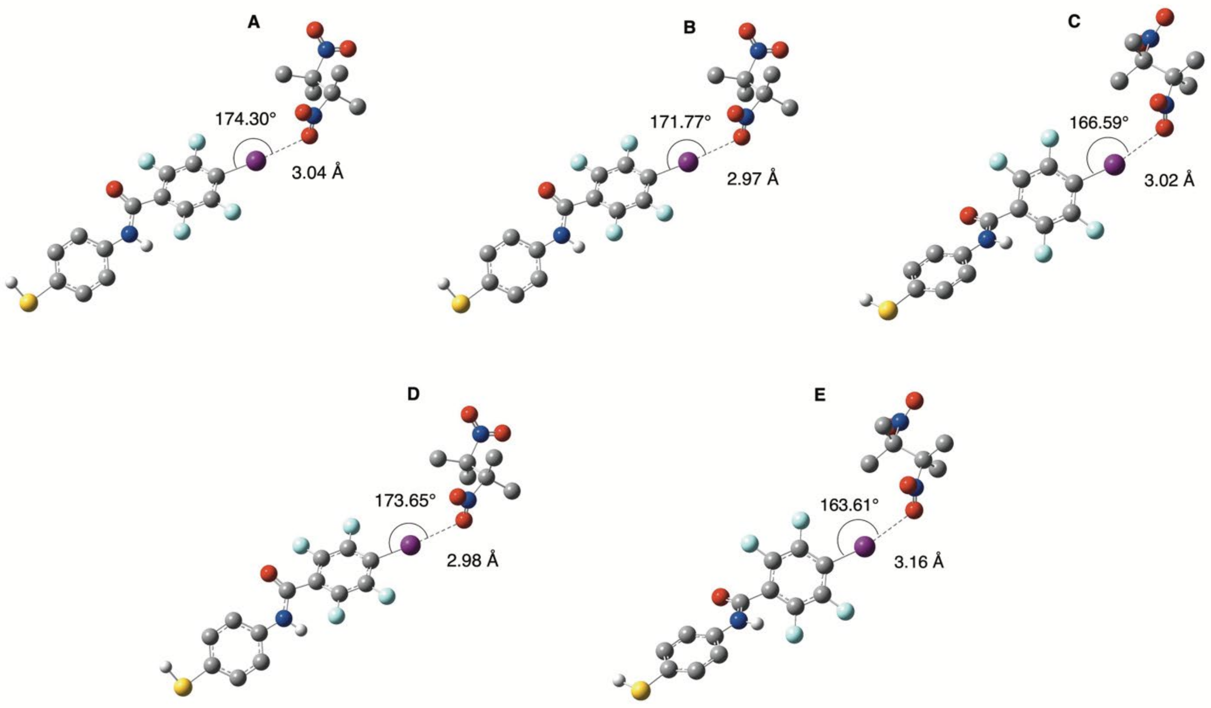

Figure SI-34. Geometry optimizations of the DMNB - L2 interaction complex at three levels of theory A) B3LYP/cc-pVDZ (Gas), B) M06-2x/cc-pVDZ//M06-2x/cc-pVTZ (Gas), C) wB97-XD/cc-pVDZ// ωB97X-D/cc-pVTZ (Gas), D) M06-2x/cc-pVDZ//M06-2x/cc-pVTZ (Solvent = water), E) ωB97X-D/def2-STVDP (Gas).

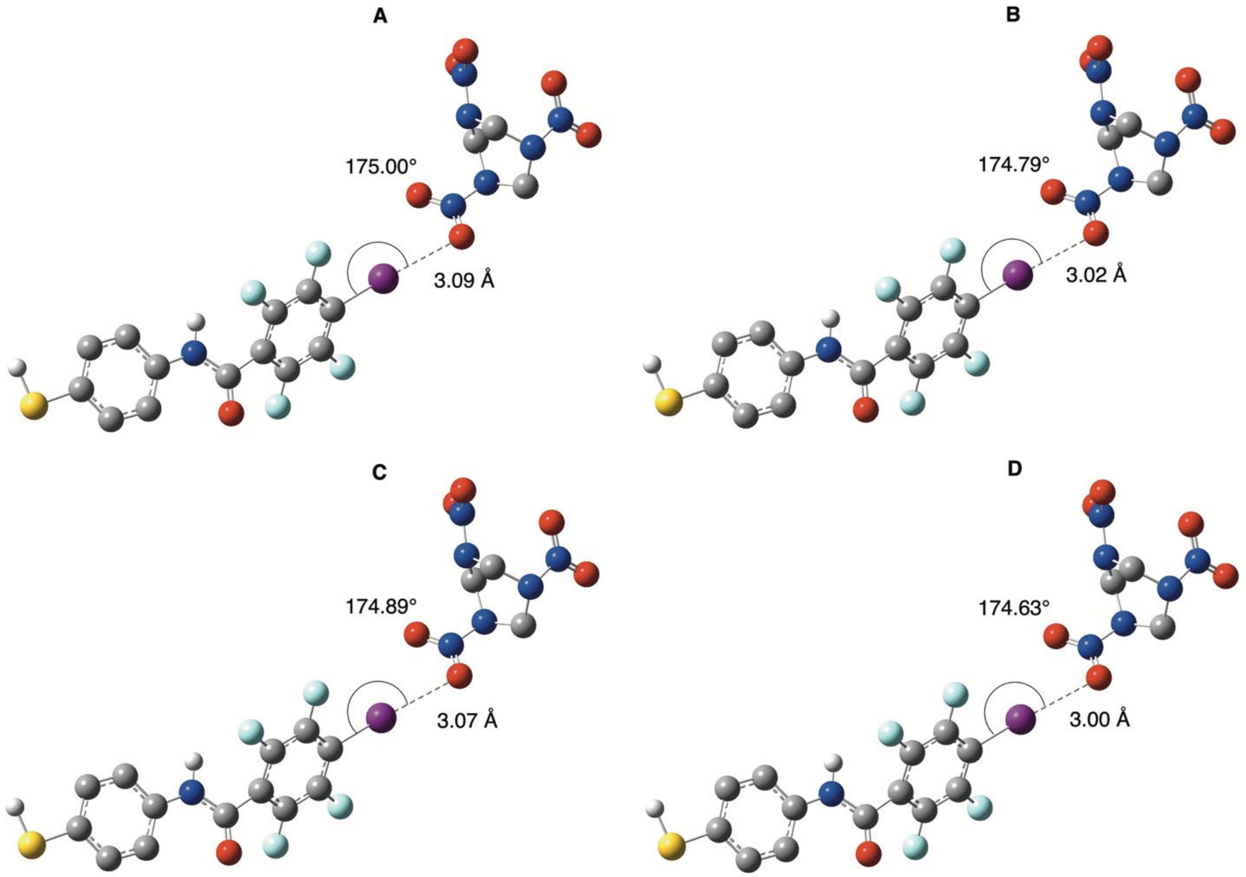

Figure SI-35. Geometry optimizations of the RDX - L2 interaction complex at three levels of theory A) B3LYP/cc-pVDZ (Gas), B) M06-2x/cc-pVDZ//M06-2x/cc-pVTZ (Gas), C) wB97X-D/cc-pVDZ//ωB97X-D/cc-pVTZ (Gas), D) M06-2x/cc-pVDZ//M06-2x/cc-pVTZ (Solvent = water).

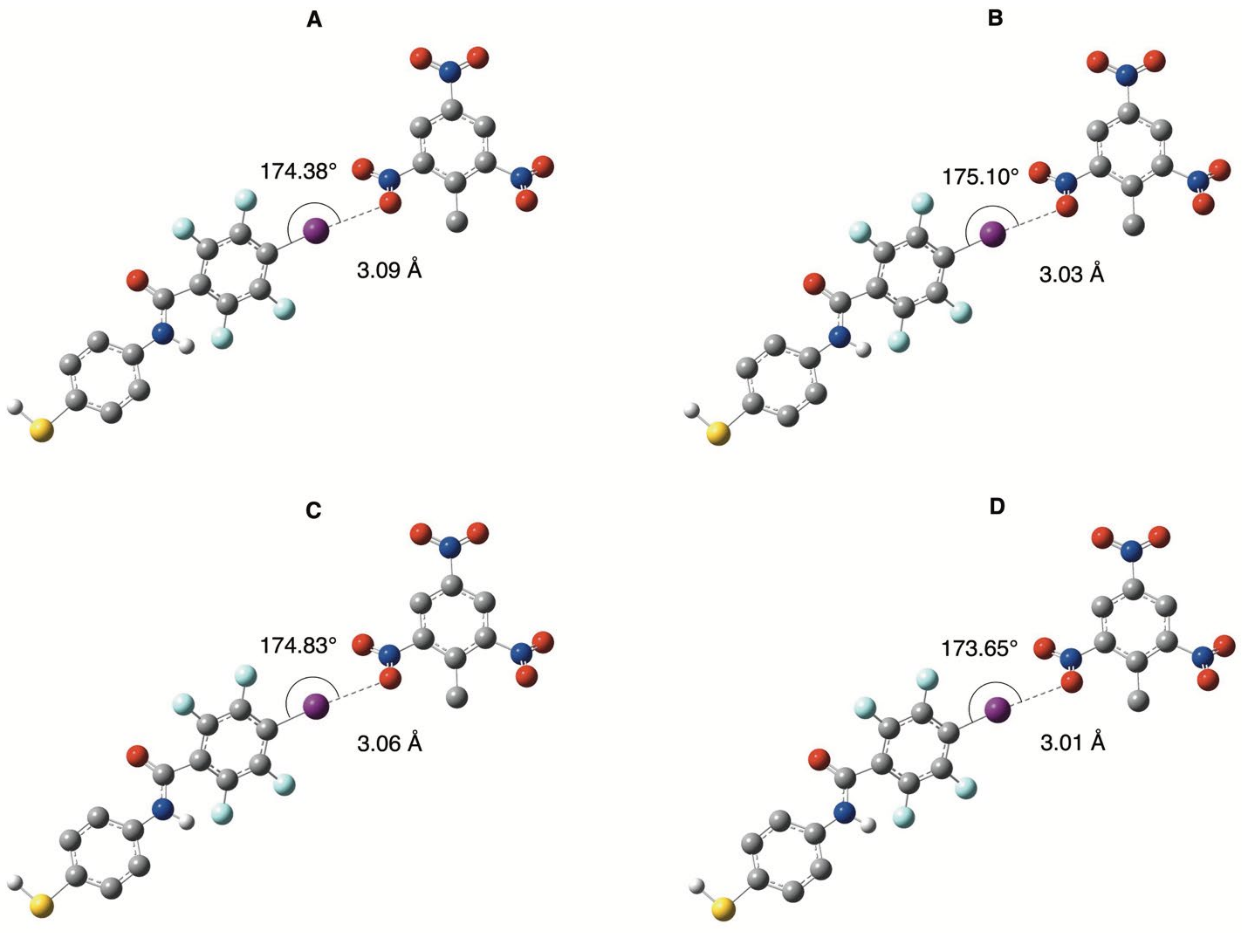

Figure SI-36. Geometry optimizations of the TNT site1 - L2 interaction complex at three levels of theory A) B3LYP/cc-pVDZ (Gas), B) M06-2x/cc-pVDZ//M06-2x/cc-pVTZ (Gas), C) ω B97X-D /cc-pVDZ//wB97X-D/cc-pVTZ (Gas), D) M06-2x/cc-pVDZ//M06-2x/cc-pVTZ (Solvent = water).

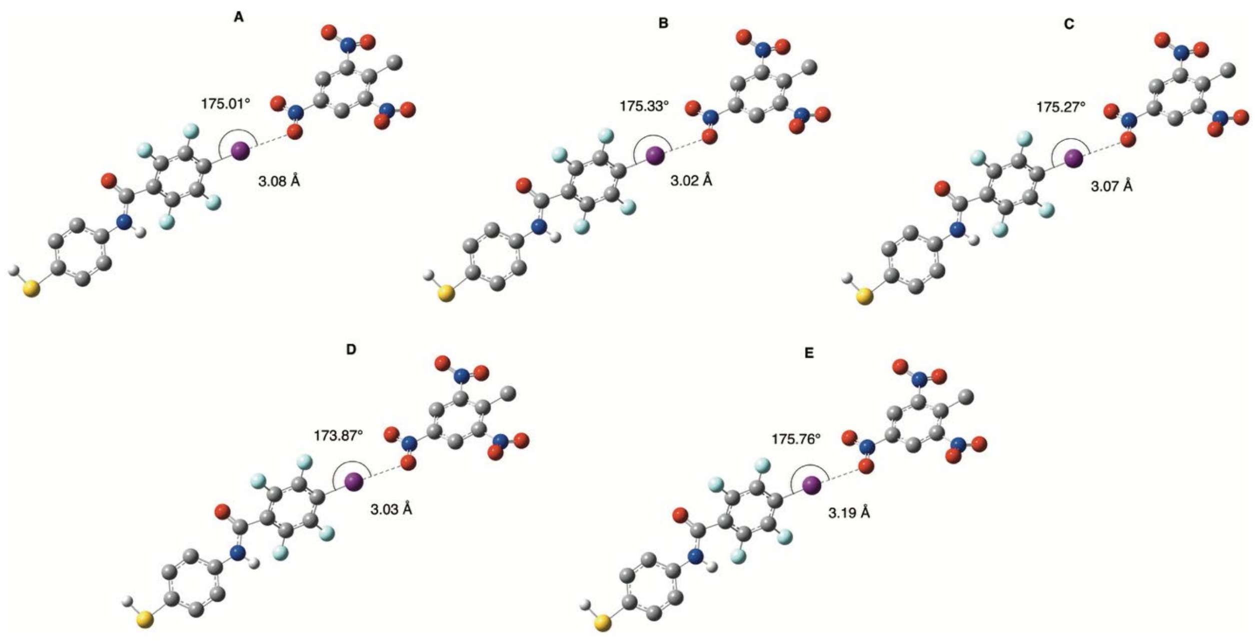

Figure SI-37. Geometry optimizations of the TNT site2 - L2 interaction complex at three levels of theory A) B3LYP/cc-pVDZ (Gas), B) M06-2x/cc-pVDZ//M06-2x/cc-pVTZ (Gas), C) ωB97X-D/cc-pVDZ// ωB97X-D/cc-pVTZ (Gas), D) M06-2x/cc-pVDZ//M06-2x/cc-pVTZ (Solvent = water), E) ω B97X-D/def2-STVDP (Gas).

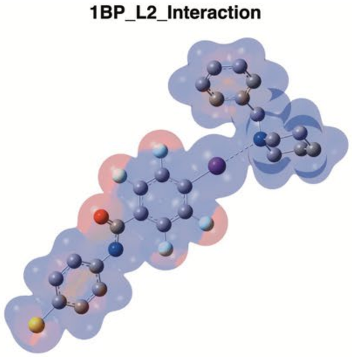

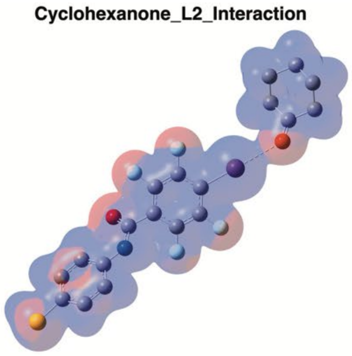

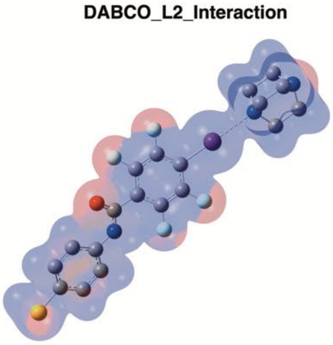

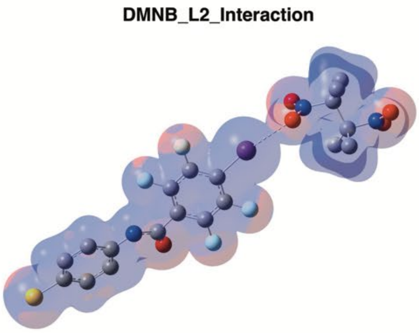

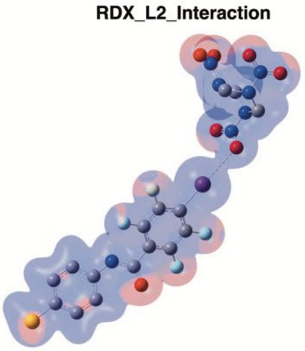

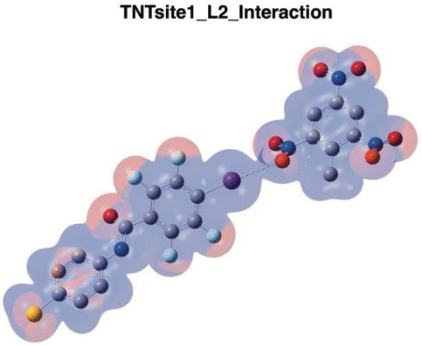

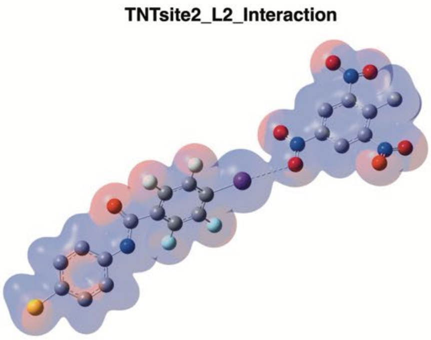

Figure SI-38. Electrostatic potentials (ESPs) in atomic units on a 0.01 electrons/bohr^3 isodensity surface for the dimer interactions between the analytes and L2. ESP values range from -2.0×10^{-5} au (red) to $+2.0\times10^{-5}$ au (blue). Taken at the M06-2x/cc-pVTZ//M06-2x/cc-pVDZ level of theory.

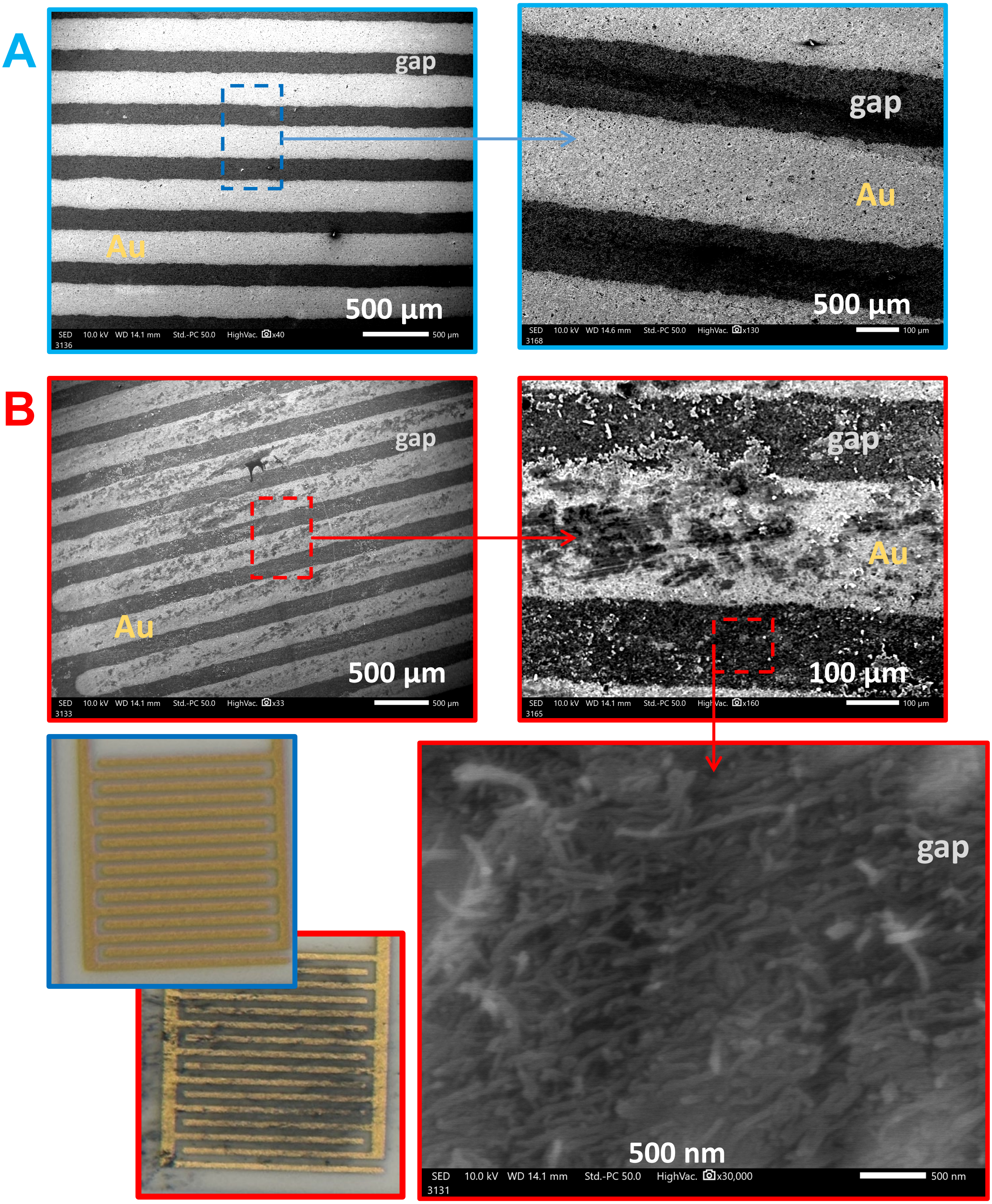

Figure SI-39. Pictures (lower left) and SEM imaging examples of **(A)** clean and **(B)** SWCNT/f-MPC(L2) modified IDAs at various magnifications.

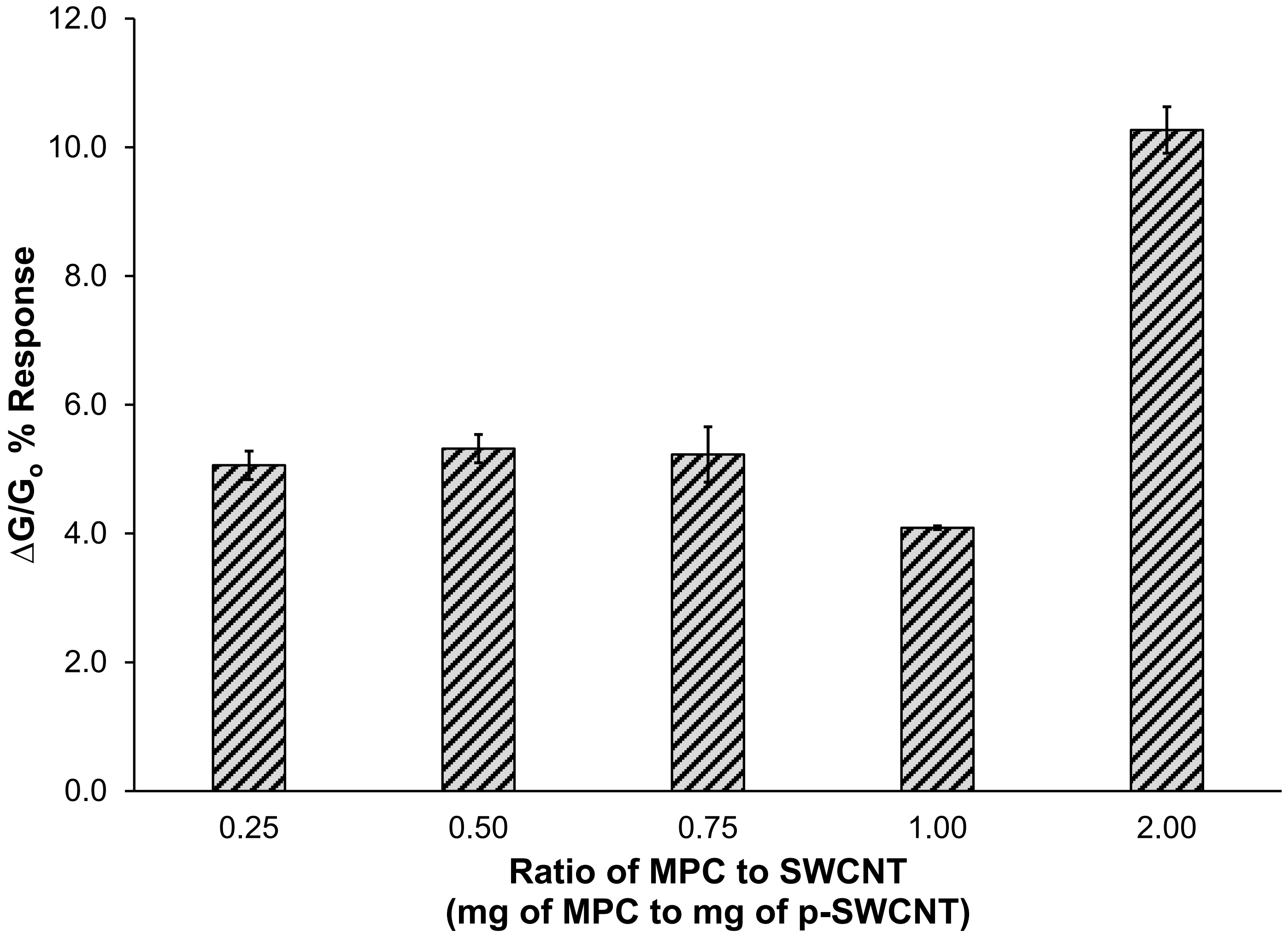

Figure SI-40. Films of different mass ratios of f-MPC to SWCNT to determine optimal mass ratio for material coated on the IDA and exposed to 50% CH vapor. From these results (n=3-5), a 2:1 ratio appears to have increase sensitivity.

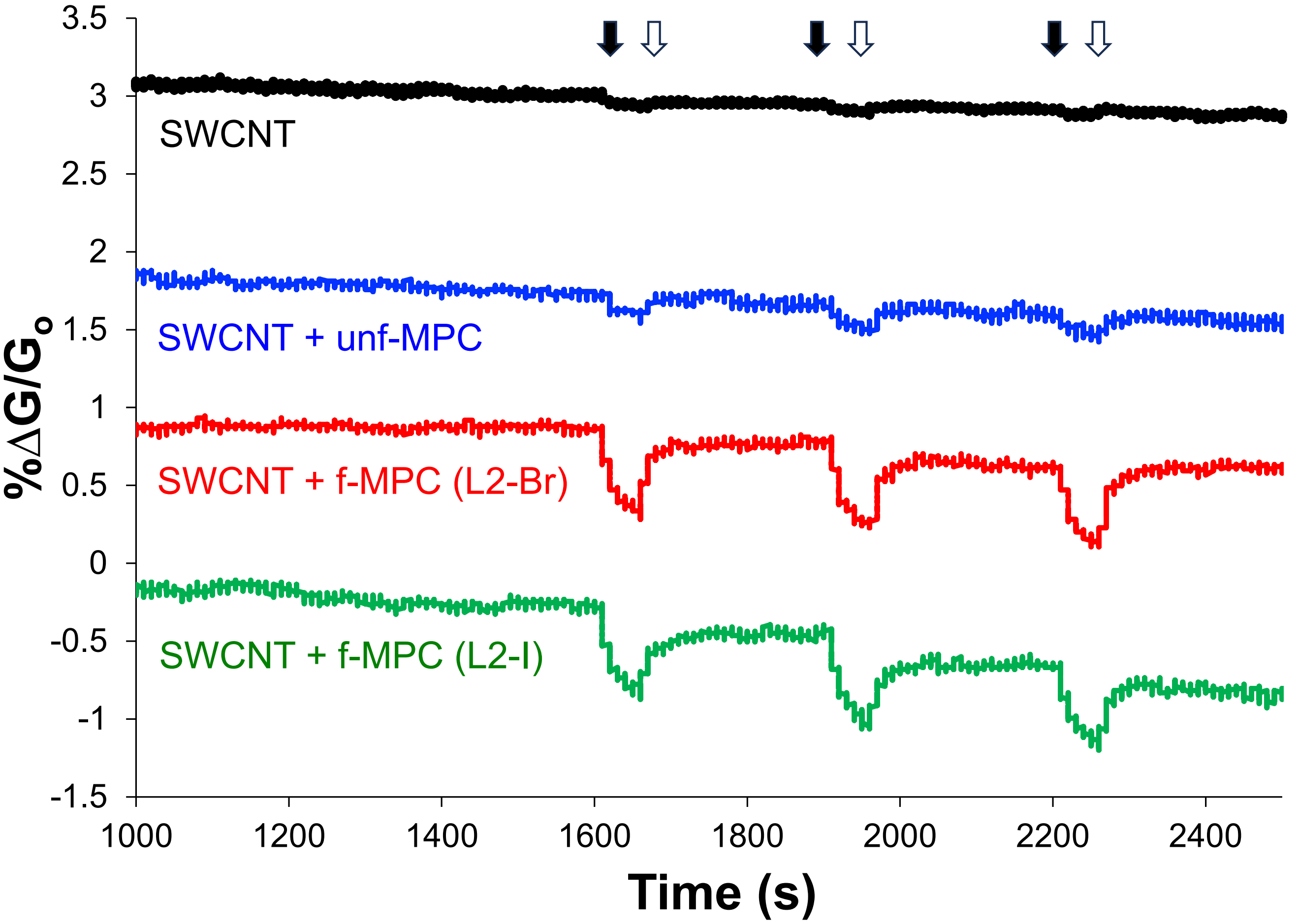

Figure SI-41. Sensing response of normalized conductance [$\Delta G/G_o\%$] of SWCNT-based films featuring SWCNTs only (control), SWCNT with unf-MPCs, SWCNTS with f-MPCs (L2-Br), and f-MPCs (L2-I). Notes: For visual clarity, each result is displayed with offset of 1 $\Delta G/G_o\%$ unit; normalization/baseline correction to conductance described in Materials and Methods section.

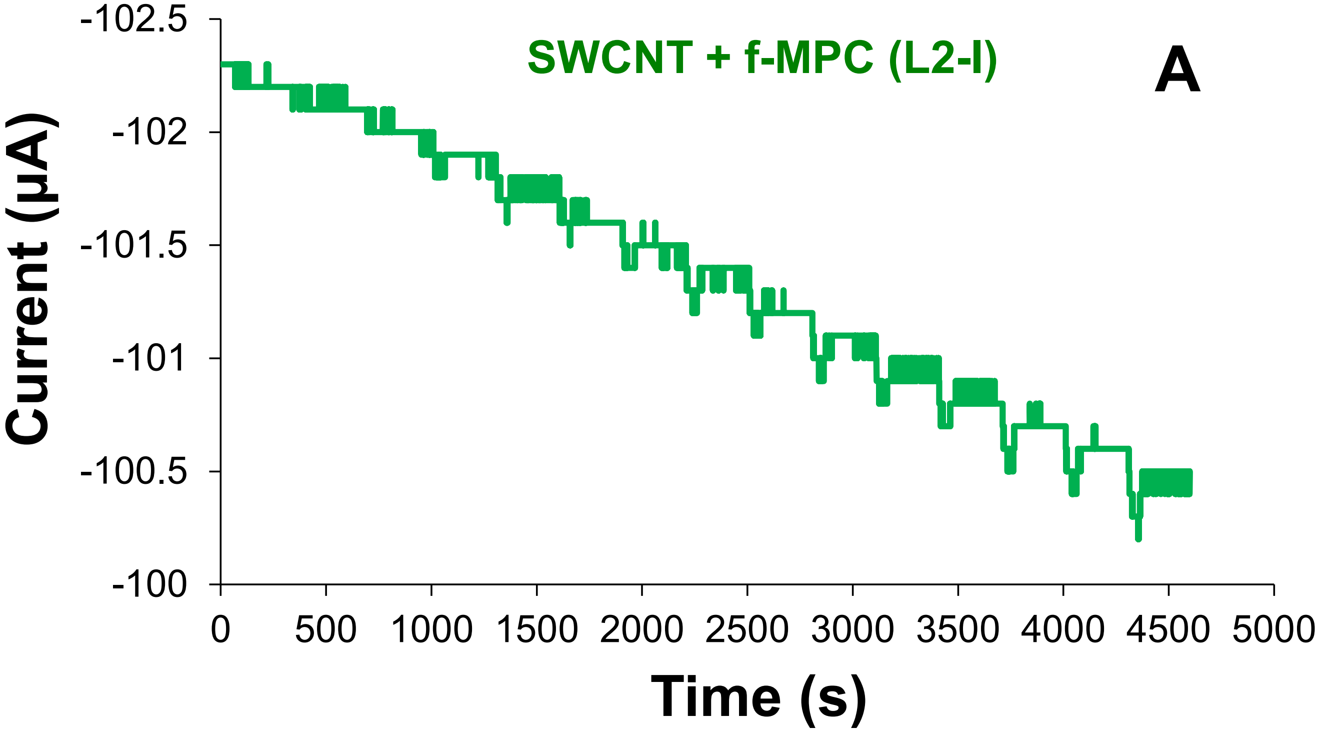

Figure SI-42. Example of sensing response in terms **(A)** current and **(B)** normalized conductance [$\Delta G/G_o\%$] of film of **SWCNTs with f-MPC (L2-I)** during exposure to increasing concentrations of CH vapor (0, 10, 20, 30, 40, 50 ppm with 3 pulses at each concentration).

A

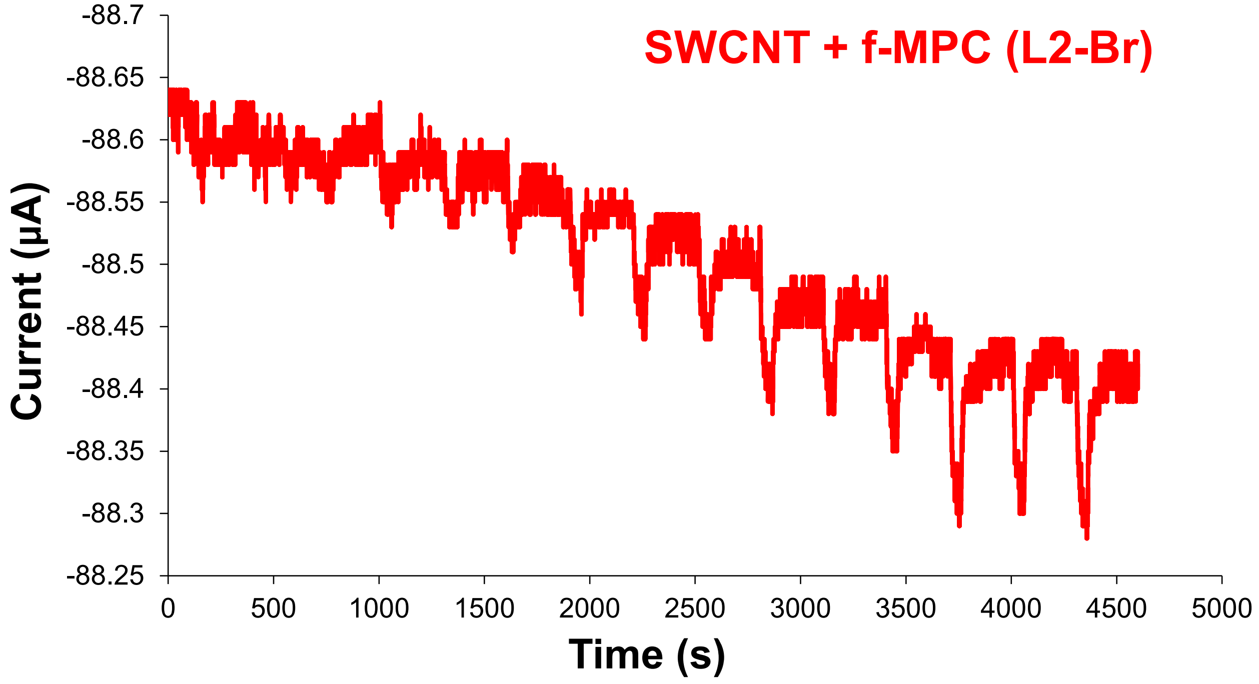

B

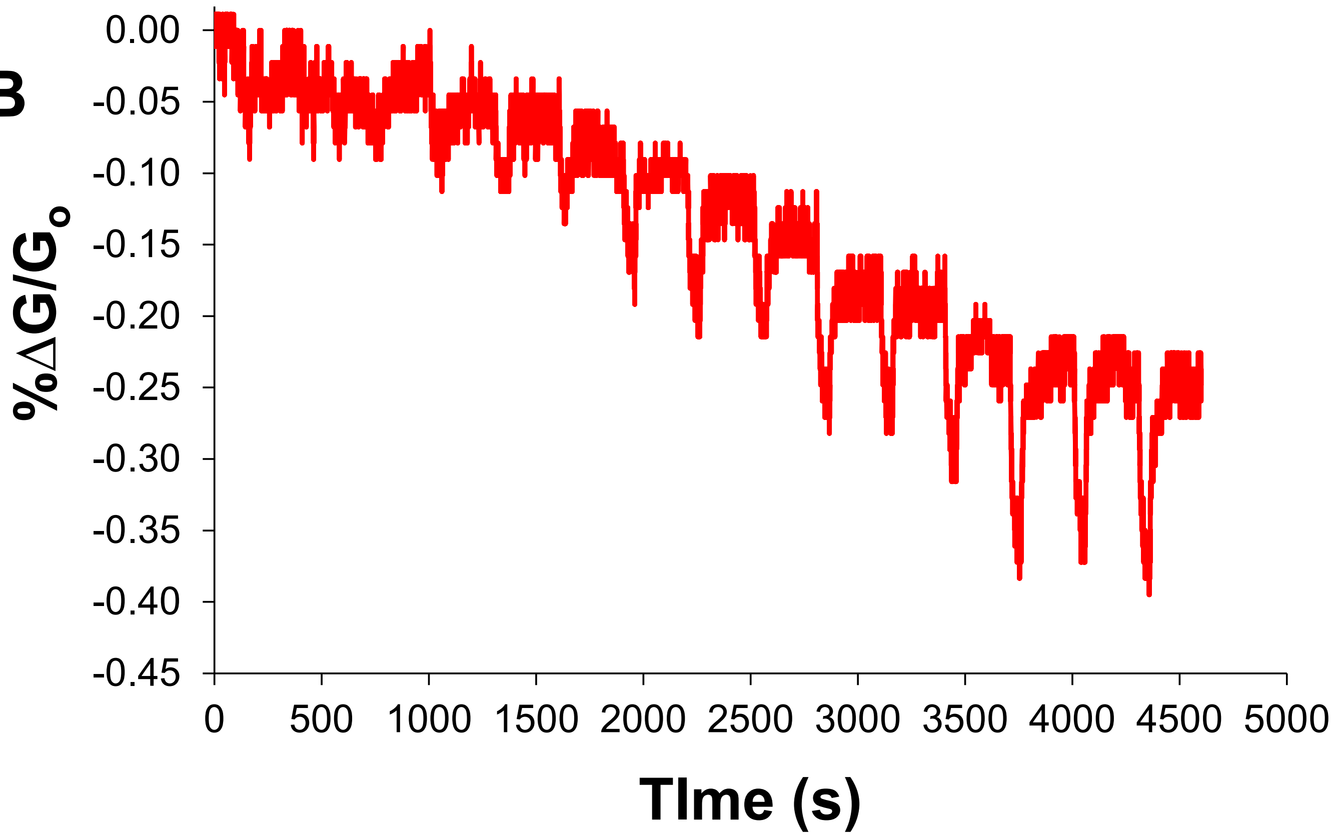

Figure SI-43. Example of sensing response in terms **(A)** current and **(B)** normalized conductance [$\Delta G/G_o$%] of film of **SWCNTs with f-MPC (L2-Br)** during exposure to increasing concentrations of CH vapor (0, 10, 20, 30, 40, 50 ppm with 3 pulses at each concentration).

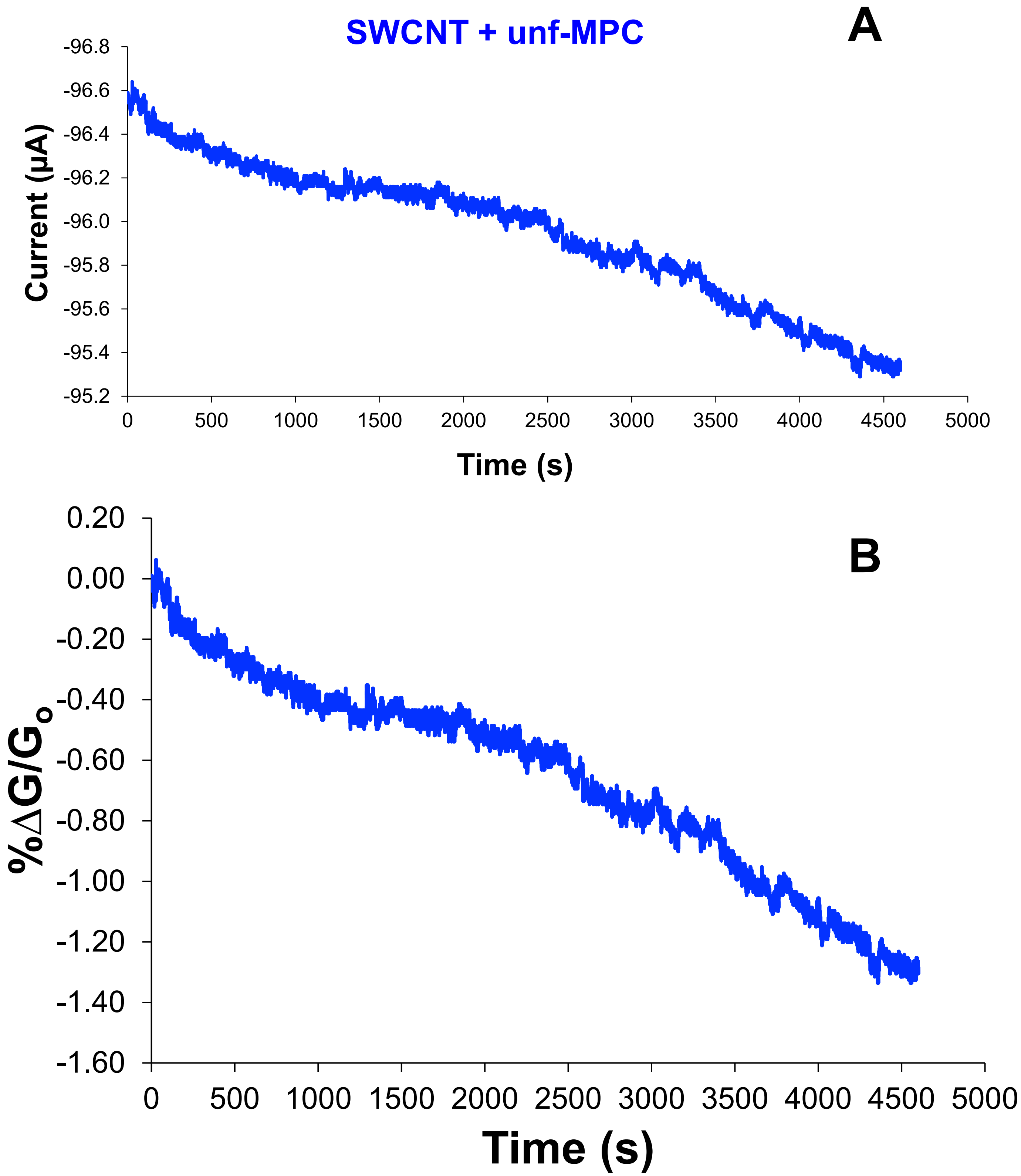

Figure SI-44. Example of sensing response in terms **(A)** current and **(B)** normalized conductance [$\Delta G/G_o\%$] of film of **SWCNTs with unf-MPC** during exposure to increasing concentrations of CH vapor (0, 10, 20, 30, 40, 50 ppm with 3 pulses at each concentration).

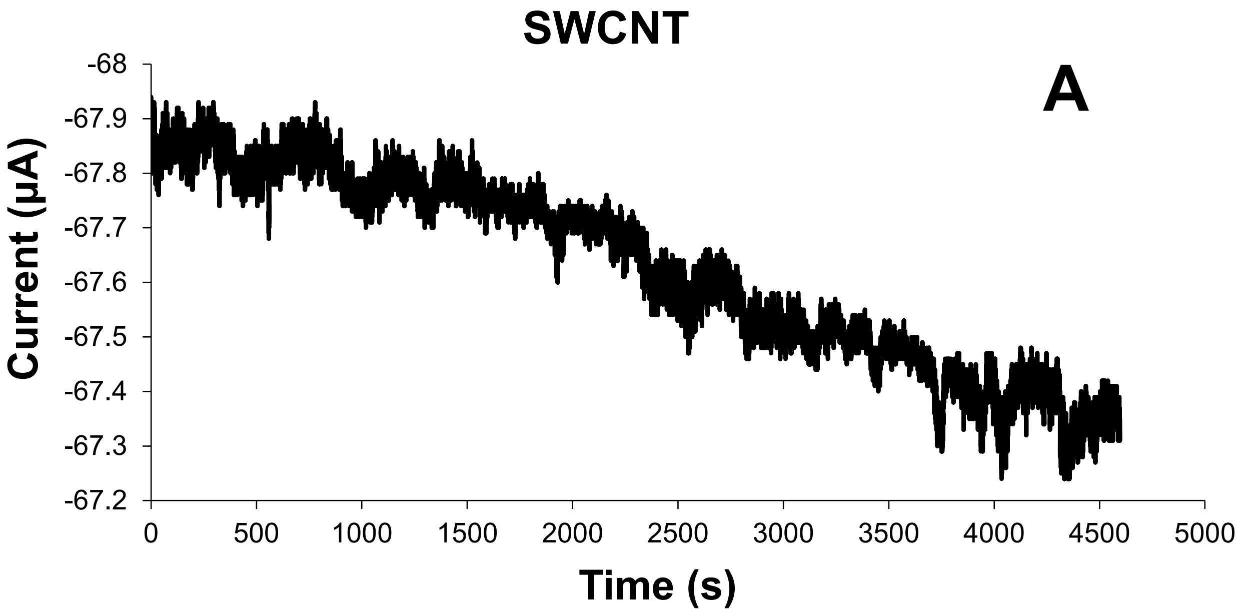

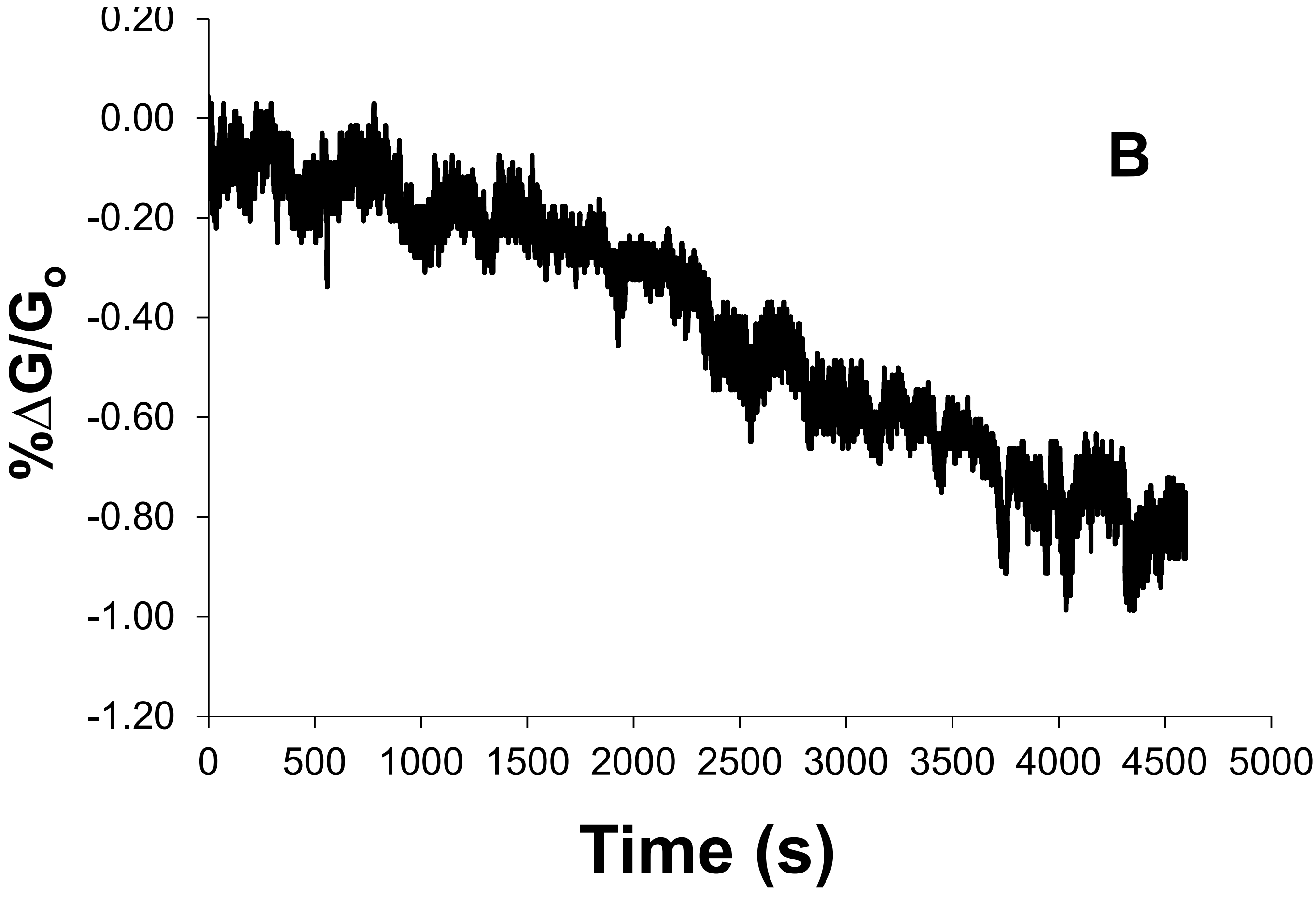

Figure SI-45. Example of sensing response in terms **(A)** current and **(B)** normalized conductance [$\Delta G/G_o$%] of film of **SWCNTs (control)** during exposure to increasing concentrations of CH vapor (0, 10, 20, 30, 40, 50 ppm with 3 pulses at each concentration).

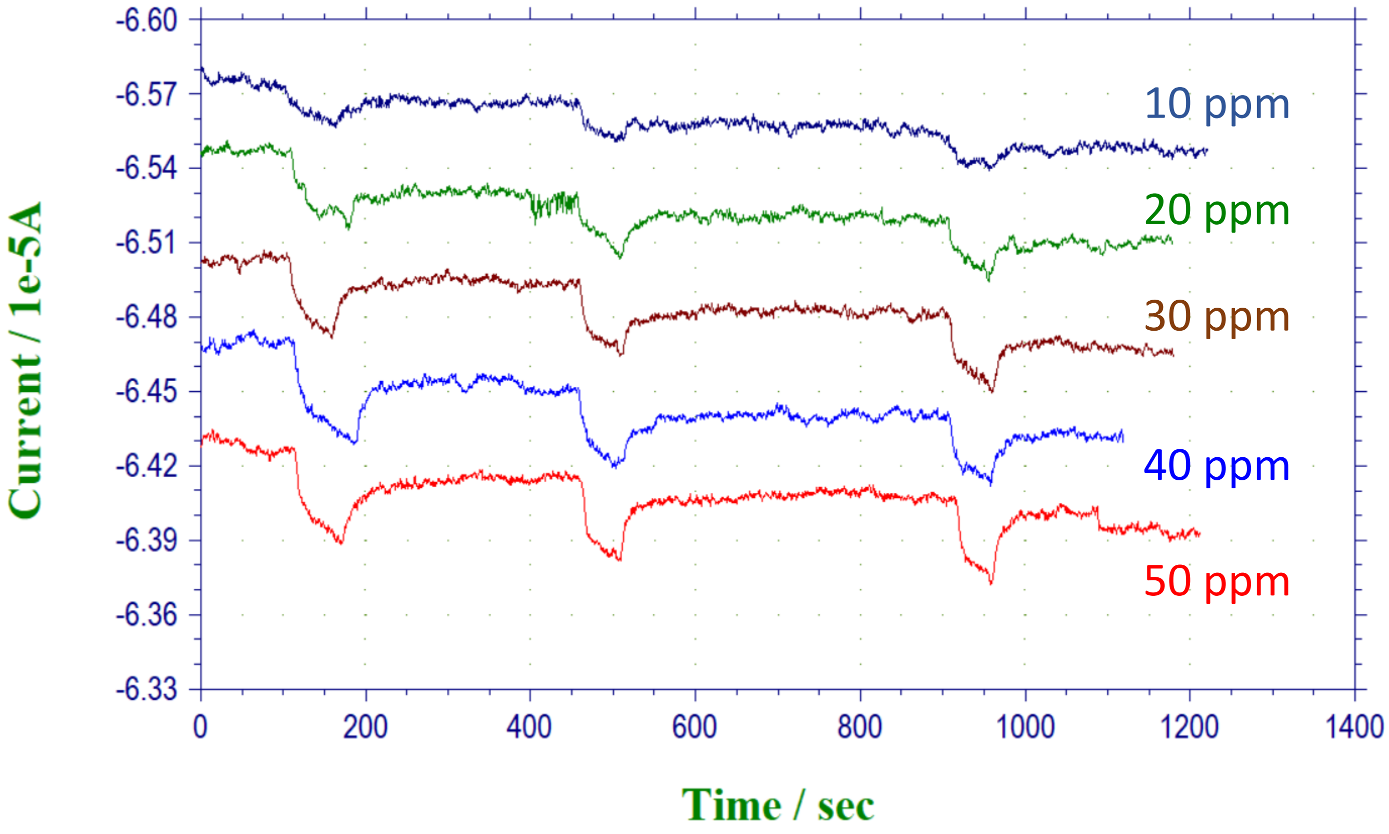

Figure SI-46. Example of I-t curves collected as three successive pulses of different concentrations of CH vapor flow over an IDA modified with SWCNTs with f-MPC (L2-I). Analogous results for films of SWCNTs with f-MPCs (L2-Br) are also available (not shown).
